# Preconception exposures of female mice to a panel of metabolic disruptors induce sexually dimorphic metabolic perturbations in their offspring

**DOI:** 10.3389/fendo.2026.1787973

**Published:** 2026-04-02

**Authors:** Carlos Diaz-Castillo, Stephanie R. Aguiar, Raquel Chamorro-Garcia

**Affiliations:** 1 Department of Microbiology and Environmental Toxicology, University of California, Santa Cruz, Santa Cruz, CA, United States; 2 Department of Molecular, Cell and Developmental Biology, University of California, Santa Cruz, Santa Cruz, CA, United States

**Keywords:** A*norexia nervosa*, chromatin organization, epigenetics, high-fat diet, inorganic arsenic, multigenerational metabolism-disrupting agents, total Western diet, tributyltin

## Abstract

In recent years, there has been a growing emphasis on research investigating the transmission across generations of the effects of exposure to environmental factors, which may predispose to chronic diseases. It has been hypothesized that the propagation of such effects is mediated by alterations in gene regulatory elements, such as DNA methylation, histone modifications, and non-coding RNAs. Studies in Drosophila and mice have demonstrated that the compartmentalization of eukaryotic genomes into heterochromatin and euchromatin can mediate multigenerational metabolism-disrupting effects elicited by exposure to various metabolism disruptors. These findings suggest that eukaryotic nuclear genomes may possess the capacity to integrate the impact of environmental cues in a metastable manner that is phenotypically relevant. Here, we present the results of a murine model to assess whether preconception exposure to three metabolism disruptors of distinct nature, including dietary factors and environmental toxicants, intended to emulate the complexity of human exposures, results in metabolic alterations in the offspring of exposed individuals. Our findings align with our central hypothesis but also open an unexpected avenue to explore whether preconception exposure to metabolism disruptors predisposes the offspring of exposed individuals to not only typical metabolic diseases such as obesity but also to complex metabolic-psychiatric conditions such as anorexia.

## Introduction

Over the past two decades, substantial evidence has emerged demonstrating the significant role of environmental agents, including chemical, biological, physical, or psychosocial factors, in determining the prevalence of chronic noncommunicable diseases within human populations (1). Obesity is an example of a chronic metabolic condition whose prevalence has dramatically increased in the last 50 years, and only ~2.7% of cases can be attributed to genetic factors (2). Exposure to metabolism disruptors, including pesticides, stress, and microorganisms, increases the susceptibility to developing chronic metabolic conditions, such as obesity, which can be propagated across multiple generations (3). These exposures can alter lipid metabolism, energy balance, appetite regulation, and glucose homeostasis by interfering with hormone signaling pathways, including those involving insulin, leptin, and nuclear receptors (4).

Animal model research has been instrumental in assessing the significance of the impact of environmental exposures on health outcomes for exposed individuals and their subsequent generations, as well as determining their underlying mechanisms (5). Beginning in 2013, we demonstrated that exposure to the biocide tributyltin (TBT) in female mice one week prior to conception and during the *in utero* development and lactation of their offspring resulted in sexually dimorphic metabolic alterations in at least four offspring generations (F1, F2, F3, and F4) (6–9). To elucidate the underlying mechanism mediating the transgenerational metabolic disruption induced by TBT, we devised a series of integrative analyses of several traits related to the epigenomic function (7, 8). We compared epigenetic marks such as DNA methylation at a genomic scale and other structural and genomic traits known to be regulated by epigenetic modifications, including chromatin accessibility and gene expression, in the somatic tissues and mature male gametes of the F3 and F4 descendants of F0 females that had been exposed to TBT and controls (7, 8). In most of our analyses, we detected a dichotomy for TBT-dependent alterations with regard to the heterochromatic and euchromatic compartments of the mouse genome; changes in a particular trait were significantly biased in one direction in heterochromatin and the opposite direction in euchromatin (7, 8). In conjunction with our investigation of the distribution within the mouse genome of genes regulating metabolism and chromatin organization, we proposed that the most plausible mechanism underlying the TBT-dependent multigenerational effects is that TBT triggered a perturbation of chromatin organization with the ability to self-reconstruct through development and across generations and predispose the progeny of exposed females to metabolic disorders (7, 8). Although our analyses did not directly address which layers of chromatin organization were altered upon TBT exposure, our findings were consistent with the possibility that such perturbation encompassed an alteration of the heterochromatin/euchromatin compartmentalization (HEC) (7, 8).

To the best of our knowledge, the ability of HEC to function as an epigenetic mechanism of genome regulation capable of mediating multigenerational metabolic disruptions has only been demonstrated in a single study on *Drosophila melanogaster*. In 2014, Öst et al. observed a similar dichotomy in gene expression alterations for heterochromatic and euchromatic genes in the offspring of male *Drosophila melanogaster* subjected to diets with varying sugar content (10). Exposure to these diets also led to alterations in metabolic traits and the expression of heterochromatin markers (10). The association between the dichotomy of perturbations in epigenomic function traits and metabolic disorders in the descendants of individuals exposed to environmental modulators of metabolism in two independent studies could suggest that eukaryotic HEC is generally susceptible to modulation by environmental exposure to metabolism disruptors in a metastable manner, resulting in metabolic effects in the descendants of exposed individuals (7, 8, 10). Notably, these two studies differ in the nature, duration, and sex of exposure, the number of generations separating exposed and descendants, and even the epigenetic characteristics of the species under study. *Drosophila melanogaster* has very low levels of DNA methylation when compared to other species such as *Mus musculus (*11).

In this study, we aimed to determine whether preconception exposure of female mice to three distinct metabolism disruptors alters the metabolism of their F1 offspring through chromatin organization modifications, particularly in HEC. Historically, research on the effects of multigenerational effects resulting from female environmental exposures has predominantly focused on exposure paradigms that encompassed the pre- and perinatal development of the offspring of exposed females (12). Consequently, there has been limited attention given to the effects of female exposures that occurred solely before conception (12). However, existing knowledge regarding the developmental establishment of HEC and the relevance of human preconception exposures that elicit multigenerational chronic metabolic diseases suggests that the preconception exposure window can be a relevant window of susceptibility for environmental exposures disrupting the early establishment of HEC in the offspring of exposed individuals.

On the one hand, research conducted across multiple eukaryotic species employing diverse methodologies has demonstrated that the earliest indications of genome compartmentalization and heterochromatin formation emerge very early in metazoan development, predating the full activation of the zygotic genome that characterizes the maternal-to-zygotic transition (MZT) (13, 14). Furthermore, the study of reporters of heterochromatic function in *Drosophila melanogaster* has suggested that adult heterochromatin structures are established very early in development and are faithfully propagated throughout development (15). Recent theoretical modeling for the propagation of heterochromatin-related epigenetic marks indicates that the spatial compartmentalization of heterochromatin and the limited availability of enzymes essential for its maintenance are necessary for the faithful propagation through cell differentiation and development of heterochromatin-related structures (16). Given that during the MZT transition, the zygote is largely transcriptionally silent (17), all processes occurring along MZT, including the establishment of heterochromatin, occur at the expense of the limited amounts of material deposited in the oocyte and to a lesser extent in the sperm. Considering these factors, it appears plausible that any environmental exposure resulting in alterations of the gamete material required for the establishment of the zygotic heterochromatin subsequently led to perturbations of heterochromatin that are propagated throughout development, thereby mediating phenotypes that reflect the biased localization of genes with specific functionalities in relation to heterochromatin (8, 10).

On the other hand, although human multigenerational studies are limited, the few that exist support the hypothesis that exposure to environmental stressors before conception contributes to chronic conditions in their offspring of at least two generations. The Dutch Famine stands as a pivotal human study that elucidates the significance of identifying critical windows of susceptibility to environmental stressors and chronic disease prevalence in subsequent generations (18). Numerous analyses of this cohort have demonstrated that low calorie intake of women during early, mid, and late gestation can result in chronic metabolic, psychological, and immunological disorders in their progeny (18). Notably, the descendants of the early exposure group exhibited a substantial increase in obesity, coronary heart disease, and elevated blood cholesterol levels compared to those whose mothers were exposed to famine during mid and late gestation (18). Given that the early exposure to famine spanned 10 weeks before and 10 weeks after conception, the higher disease incidence in the descendants of this group could support the hypothesis that the preconception window is particularly susceptible to the action of environmental exposures with effects in the next generation. Other human studies have shown that alterations in food availability or tobacco use during puberty have an effect on their grand offspring (19, 20). Altogether, these findings underscore the profound impact of preconception and early gestation events on transgenerational health and disease outcomes.

The selection of the metabolism disruptors for our study was based on the recognition that humans are subjected to a multitude of environmental stressors of varying nature from the moment of conception until death. This concept is encapsulated within the exposome framework (21), which encompasses the intricate interplay between life experiences and exposures that contribute to modulating susceptibility to disease. We selected three metabolism disruptors, TBT, inorganic arsenic (iAs), and diet, to model real-life human exposures of very different nature. TBT is an example of a human-made metabolism-disrupting chemical commonly found in soil, dust, and water and in human samples from liver, placenta, and blood, that has been associated with multigenerational obesity (6–9, 22). iAs is a naturally occurring element ubiquitously found in soils, sediment, and groundwater, and it poses major threats to global public health as around 200 million people worldwide are exposed to high concentrations (23). Prenatal exposure to arsenic has been associated with increased adiposity and increased risk of type-2 diabetes in human populations (24). Maternal preconception exposure to environmentally relevant doses of iAs in mice led to transgenerational metabolic perturbations, but little is known about potential epigenetic alterations associated with such transgenerational phenotypes (25). Lastly, total Western diet (TWD), whose micro- and macro-nutrient content represents the diet 50% of the U.S. population takes, is an example of a lifestyle metabolism disruptor (26). Although the separate exposure to these three metabolism disruptors will not conveniently model the complexity of the exposome, it can serve as a stepping stone in our understanding of whether environmental factors humans are exposed to and that have been associated with metabolic disorders do so through similar mechanisms of action.

## Methods

### Mouse work

We conducted all mouse procedures at the University of California, Santa Cruz (UCSC) Vivarium. These procedures were reviewed and approved by the UCSC Institutional Animal Care and Use Committee (UCSC IACUC) as part of the animal protocol Chamr1908.

We purchased 160 female and 80 male C57BL/6J mice from Jackson Laboratory (strain #000664). To acclimate mice to the UCSC Vivarium, we scheduled their arrival a week before the start of the experiment. Upon arrival, we ear-punched and weighed the female mice and housed them in four-mouse cages. To minimize initial body weight disparities between the experimental groups, we randomly assigned the cages to five groups. We ranked the cages based on the cumulative body weights of the mice within each cage, from highest to lowest. Then, we divided the ranked list into eight subgroups of five cages each and randomly assigned each cage within each subgroup to one of the experimental groups.

We exposed 5-week-old female mice in each group to the following combinations of treated drinking water and diet for 3.5 weeks:

- DMSO (negative control) group: 0.1% dimethylsulfoxide (DMSO) in water and control diet (CD; Envigo Teklad Diets TD.140148).
- 5TBT group: 5 nM tributyltin (TBT) and 0.1% DMSO in water and CD.
- 50TBT group: 50 nM tributyltin and 0.1% DMSO in water and CD.
- IAS group: 10 µg/L sodium meta(arsenite) (inorganic arsenic, iAs) and 0.1% DMSO in water and CD.
- TWD group: 0.1% DMSO in water and Total Western Diet (TWD; Envigo Teklad Diets TD.110919).

The Agency for Toxic Substances and Disease Registry (ATSDR) determined that the no-observed adverse effect level (NOAEL) for TBT is 0.025 mg/Kg/day (27). 50 nM TBT in drinking water is equivalent to 0.005 mg/Kg/day, assuming an average mouse weight of 30 g and an average of 10 mL of water taken per day. Thus, 5 and 50 nM TBT represent 50 and 5 times lower than the NOAEL, respectively. We used 10 µg/L of inorganic arsenic in the drinking water, as that is the allowable level established by the U.S. Environmental Protection Agency (28). Low-fat CD and high-fat TWD have energy densities of 3.8 and 4.4 Kcal/g, respectively, and 17.2 and 34.5% of their energy content is provided by fat, respectively. TWD represents the diet 50% of U.S. people eat on a regular basis (26).

Immediately before starting the experiment and weekly thereafter, we weighed the mice before and after a four-hour fast. To protect female mice from any adverse effects of fasting on reproduction, we did not fast them at the end of the exposure period and before mating. We replenished the mouse feed weekly and water bottles semiweekly to maintain freshness and control consumption.

After 3.5 weeks of exposure at 8 weeks of age, we mated exposed female mice with unexposed same-age male mice for a week to produce F1 offspring. We checked females daily for successful matings by observing copulatory plugs. Mice with plugs were returned to their same cage mates from the exposure period for the rest of the pregnancy. Two days before birth, we relocated pregnant females to individual cages with ample bedding for nest-building. We monitored births and litter welfare using minimally invasive methods until the pups were old enough for further manipulations.

At 8–11 days of age, we toeclipped F1 mice. At 3 weeks of age, we weaned and selected at least 10 F1 mice per sex and exposure group. We housed mice of the same sex and exposure group together. We weighed F1 mice at weaning and weekly thereafter until the end of the experiment. We fed F1 mice CD between 3 and 7 weeks of age and TWD between 7 weeks of age and the end of the experiment. Before changing diets at 7 weeks of age, we weighed F1 mice before and after fasting for 12 hours. We measured fasting glucose using a Contour^®^ blood glucose meter and strips (Ascensia Diabetes Care) with blood drawn from the mouse tails. At 12 weeks of age, we fasted F1 mice for 12 hours and euthanized them using an overdose of isoflurane followed by cardiac exsanguination. We determined fasting glucose levels from peripheral blood as previously indicated and harvested gonadal and inguinal white adipose tissue (gWAT and iWAT, respectively) depots and liver. We drew blood from the heart using EDTA-flushed syringes to minimize coagulation and collected cardiac blood samples in microcentrifuge tubes with 6 µL of a 100X protease inhibitor cocktail (Sigma Aldrich NC2042678). We centrifuged blood samples at 5,000 x g for 10 minutes at 4°C to separate plasma. We weighed gWAT, iWAT, and liver samples and snap-frozen them in dry ice. We maintained plasma, gWAT, iWAT, and liver samples frozen for downstream analyses. Dissections were performed by trained Chamorro-Garcia group members. To minimize daily workload, we divided F1 mice into five groups with equal representation of each exposure group and sex. Each group underwent dissection over five consecutive days. The lead experimenter (Diaz-Castillo) randomly assigned mice to dissectors who were unaware of the group or sex of the mice.

### Metabolic analyses

We determined body weight by placing each mouse in an empty beaker atop a standard laboratory balance (**Supplementary Data 6**, **7**, **12**, **13**, **15**). To minimize age-related differences, especially in younger F1 mice, we corrected body weight data by the days of age of each mouse. We assessed the significance of body weight differences between exposure groups (5TBT, 50TBT, IAS, and TWD) and the control group (DMSO) using unmatched-measures Monte Carlo-Wilcoxon (uMCW) tests (see Statistics section) (**Supplementary Data 21**). We assessed the significance of body weight changes associated with fasting challenges between exposure and control groups using matched-bivariate Monte Carlo-Wilcoxon (mbMCW) tests (see Statistics section) (**Supplementary Data 22**).

To determine water consumption, we filled water bottles in each cage with 200 mL of treated water (entry water) and measured the volume of the remaining water after three or four days (exit water) (**Supplementary Data 8**). To determine food consumption, we weighed the food provided in each cage (entry food) and the remaining food after one week (exit food) (**Supplementary Data 9**, **10**). We used mbMCW tests to assess the significance of differences in entry and exit food and water between exposure and control groups (**Supplementary Data 22**).

We corrected plasma glucose levels of fasted mice by body weight and the days of age of each mouse (**Supplementary Data 14**, **15**). We used uMCW tests to assess the significance of fasting glucose differences between exposure and control groups (**Supplementary Data 21**).

We weighed gWAT, iWAT, and liver samples harvested from F1 mice using a precision balance (**Supplementary Data 15**). We corrected tissue weights by body weight and the days of age of each mouse. We used uMCW tests to assess the significance of tissue weight differences between exposure and control groups (**Supplementary Data 22**).

We submitted 50 µL of F1 plasma samples from euthanized mice to Eve Technologies (Calgary, Canada) for analysis of 11 metabolites in the Mouse, Rat Metabolic Array (MRDMET): amylin, gastric inhibitory peptide (GIP), ghrelin, glucagon-like peptide 1 (GLP-1), insulin, leptin, peptide YY (PYY), glucagon, pancreatic peptide (PP), resistin, and connecting peptide (C-Peptide) (**Supplementary Data 16**). We set to zero any data outside the standard curve range or that could not be mathematically extrapolated. We corrected plasma levels of each metabolite by body weight and the days of age of each mouse. We used uMCW tests to assess the significance of differences in plasma levels between exposure and control groups (**Supplementary Data 21**). We used the *prcomp* function from the R package *Stats* (version 4.5) (29) to perform Principal Component Analysis (PCA) on metabolites with valid data for all female or male mice to determine the similarities between experimental groups. We used the *fviz_eig* and *fviz_pca* functions from the R package *Factoextra* (version 1.0) (30) to visualize PCA results.

### Transcriptomic analyses

We extracted RNA from F1 gWAT and liver using a VWR^®^ Micro Homogenizer (catalog number #10032-328) and Qiagen RNeasy Plus kits (catalog number #74034), following the manufacturer’s protocols. We submitted the RNA samples to the University of California, Irvine (UCI) Genomics High Throughput Facility for sequencing using Illumina TruSeq Stranded mRNA kits and an Illumina NovaSeq 6000 sequencer. Libraries obtained from 100 RNA samples (2 sexes x 2 tissues x 5 experimental groups x 5 replicates per group) were sequenced up to three times to yield approximately 30 million 150-bp paired reads per sample. We used the following accessory files and three sets of informatics tools to analyze the sequencing results.

#### Accessory files for RNA-seq analyses

We downloaded FASTA files containing sequences of all major autosomes and the *X* chromosome, and the file “mm39.ncbiRefSeq.gtf.gz” with primary gene transcript annotation for the June 2020 GRCm39/mm39 mouse genome assembly from the UCSC Genome Browser (https://hgdownload.soe.ucsc.edu/downloads.html#mouse) (31). We retrieved the most recent core Gene Ontology from the Gene Ontology website (https://geneontology.org/) (32, 33).

#### Linux/Python tools

We used *isoSegmenter* (https://github.com/bunop/isoSegmenter) (34) to define isochores and isochore classes for the mouse genome. We executed *isoSegmenter* with the FASTA files for individual chromosomes downloaded from the UCSC Genome Browser. *isoSegmenter* operates in three phases: segmenting the provided sequence into non-overlapping 100 kb windows, assigning windows to five classes based on GC content, and defining isochores by concatenating juxtaposed windows of the same class. The five isochore classes and their respective GC contents are: L1 (below 37%), L2 (37%-41%), H1 (41%-46%), H2 (46%-53%), and H3 (exceeding 53%).

#### Galaxy Platform tools

We uploaded the FASTQ files obtained from the UCI Genomics High Throughput Facility to the Galaxy Platform website (usegalaxy.org) (35). We used *FastQC* (version 0.12) to assess read quality, *multiQC* (version 1.27) (36) to generate multi-sample reports, *Cutadapt* (version 5.0) (37) to remove adapter sequences, *RNA Star* (version 2.7) (38) to map reads to mouse genes, *featureCounts* (version 2.0) (39) to count reads, and *Intersect* (version 1.0) to determine the overlap of genes and isochores in the mouse genome. We used the annotation file “mm39.ncbiRefSeq.gtf.gz” to map reads to splice junctions. After completing all Galaxy Platform operations, we downloaded a table with RNA-seq gene-wise read counts for each gWAT and liver sample and isochore class associations (**Supplementary Data 17**).

#### R tools

We conducted all transcriptomic analyses using R (version 4.5) (29) packages in RStudio (Version 2024.12.1 + 563) (40). We used base R functions and the *data.table* package (version 1.17) (41) for data operations unless stated otherwise.

We used the R package *ComplexHeatmap* (version 2.24) (42) to visualize expression patterns across samples of the 100,000 most expressed genes. We aggregated raw counts for each gene across all samples and identified genes with the top 100,000 cumulative counts. We normalized gene read counts to counts per million (cpm) by dividing raw read counts for each gene by the sum of raw read counts for all genes in each sample and multiplying the result by 1 million. To rescale normalized counts to the same 0–1 range, we used the formula: (*x* - min(*x*))/(max(*x*) - min(*x*)), where *x* represents cpm values for each gene and sample, and min(*x*) and max(*x*) denote the minimum and maximum cpm values for each gene across samples, respectively.

We used uMCW tests to assess the significance of gene expression differences between exposure and control groups (see Statistics section) (**Supplementary Data 23**). We extracted read count tables for each of the 16 exposure *versus* control contrast combinations (2 sexes x 2 tissues x 4 exposure groups). We considered a gene to be expressed if at least 2 sequencing reads mapped it in at least 2 samples in each contrast and discarded non-expressed genes. We normalized gene read counts to cpm as previously indicated.

To determine the association of genes with extreme expression differences between exposure and control groups with specific functionalities, we performed pre-ranked Gene Set Enrichment Analyses (GSEA) (43) using the R package *fgsea* (version 1.34) (44) and the most updated mouse Gene Ontology (GO) annotation obtained from the R package *msigdbr* (version 10.0) (45). We restricted our analyses to GO sets of the Biological Process (BP) category with at least 15 and less than 500 genes (**Supplementary Data 24**). For each contrast, we ranked genes using the uMCW bias indexes (BIs) from highest to lowest, with the top genes being more expressed in the exposure group, and the bottom genes being more expressed in controls. GSEA calculates an enrichment score (ES) measuring the overrepresentation of a GO-BP set at either extreme of the pre-ranked list of genes, a normalized enrichment score (NES) to account for set size, and a *P* value calculated by comparing observed ES with a null distribution of ES obtained by permuting gene ranks (43).

To assess the significance of concerted changes in gene expression across the entire transcriptome and specific genomic regions such as chromosomes, individual isochores, and isochore classes, we performed biased-measures Monte Carlo-Wilcoxon (bMCW) tests (see Statistics section) (**Supplementary Data 25**, **27**). We restricted these analyses to genes on the main chromosomes of the nucleus for both sexes by filtering out genes in the mitochondrial genome, *Y* chromosome, and unassembled chromosome segments. We also filtered out non-expressed genes and normalized read counts for each sample as previously indicated.

We determined the relative fraction of genes overlapping isochores of each class for genes in each autosome and the *X* chromosome, and for genes associated with specific GO-BP terms, for each exposure *versus* control group contrast (**Supplementary Data 26**). For each chromosome, GO-BP term, and isochore class, we calculated relative gene fractions as log_10_((*x*/*n*)/(*X*/*N*)), where *x* and *X* represent the number of genes in each chromosome or associated with each GO-BP term overlapping isochores of each class or any class, respectively, and *n* and *N* represent the number of genes for the whole transcriptome overlapping isochores of each class or any class, respectively.

We used the R package *smplot2* (version 0.2) (46) to visualize the outcomes of Pearson correlation analyses between leptin plasma levels and *Lep* gene expression in gWAT, and between *Lep* and *Lnc-Lep* gene expressions in gWAT.

### Other informatic tools

We used R packages *data.table* (version 1.17) (41), *ggplot2* (version 3.5) (47), *ggplotify* (version 0.1) (48), *ggrepel* (version 0.9) (49), *ggtext* (version 0.1) (50), *patchwork* (version 1.3) (51), *RColorBrewer* (version 1.1) (52), *scales* (version 1.3) (53), and *svglite* (version 2.1) (54). The Supplementary Code file includes R code to reproduce our analyses and figures.

### Statistics

To facilitate the integrative assessment of statistical significance in the differences between exposure and control groups for litter, metabolic, and transcriptomic traits, we conducted most of our analyses using Monte Carlo-Wilcoxon (MCW) tests. Initially, we devised MCW tests to ascertain whether a set of matched-paired quantitative measures exhibited a statistically significant bias in the same direction when compared with what would be expected by chance (7, 8, 55–57). Recently, we have reformulated MCW tests to interrogate four distinct data structures and developed the R package *MCWtests (*58) to publicly distribute the functions that facilitate conducting such tests. A comprehensive description of MCW testing rationale and specific MCW tests can be found at https://diazcastillo.github.io/MCWtests/index.html. Briefly, all MCW tests follow two basic steps. Firstly, to quantitatively determine the magnitude and direction of the bias between two conditions for the measure being analyzed, MCW tests compute a bias index (BI) that spans the range of 1 to -1, indicating that the measure is completely biased in each conceivable direction. Subsequently, to ascertain the statistical significance of the BIs computed for the user-provided dataset (observed BIs), a series of expected-by-chance BIs are generated by repeatedly rearranging the original dataset and computing BIs for each iteration. *P_upper_* and *P_lower_* values are subsequently calculated as the proportions of expected-by-chance BIs that exhibit values equal to or higher than and equal to or less than the observed BIs, respectively.

MCW tests are particularly suitable for highly integrative studies like this one. Like other non-parametric approaches, MCW tests do not require original data to follow a specific distribution or undergo mathematical transformations. Also, since BI values always range from -1 to 1, comparing results from MCW tests conducted with data from different ranges, scales, or even traits becomes straightforward.

In our study, we used three of the four MCW tests offered by the R package MCWtests: unmatched-measures MCW (uMCW), matched-measures bivariate MCW (mbMCW), and biased-measures MCW (bMCW) tests.

uMCW tests assess whether two sets (*e.g.*, *a* and *b*) of unmatched measures exhibit significant bias in the same direction. The uMCW testing process involves the following steps. First, all possible disjoint data pairs using measures from both sets are drawn. Second, the second measure in each pair is subtracted from the first measure. Third, measure pairs are ranked based on the absolute value of their differences, with the lowest absolute difference receiving the lowest rank. Measure pairs whose subtraction equals 0 are assigned a 0 rank. Measure pairs with the same difference values are assigned the lowest rank. Fourth, ranks are assigned a sign based on the sign of the measure differences calculated in the third step. Fifth, signed ranks for each subtraction type (*e.g.*, *a-b* and *b-a*) are aggregated. Sixth, bias indexes (BIs) are calculated as the sum of signed ranks divided by the maximum value this sum would have if all measures in the first set (*e.g.*, *a*) were greater than those in the second set (*e.g.*, *b*). Finally, the significance of observed BIs is determined by comparing them with a collection of expected-by-chance BIs calculated after repeatedly rearranging the measure assortment between the two sets, and calculating *P_upper_* and *P_lower_* values as previously indicated. uMCW tests can follow two paths based on the user-defined parameter *max_rearrangements.* If the number of potentially distinctive data rearrangements is less than *max_rearrangements*, uMCW tests will draw all potentially distinctive rearrangements, and *P_upper_* and *P_lower_* values will represent an exact estimation of the significance of observed BIs. If the number of potentially distinctive data rearrangements is greater than *max_rearrangements*, uMCW tests will perform a number of rearrangements equal to *max_rearrangements*, and *P_upper_* and *P_lower_* values will represent an approximated estimation of the significance of observed BIs.

mbMCW tests assess whether two sets (*e.g.*, *x* and *y*) of inherently matched-paired measures are significantly differentially biased in the same direction. The mbMCW testing process involves the following steps. First, for each matched-pair of measures, the two potential subtractions are calculated (*e.g.*, *a-b* and *b-a*). Second, for each subtraction type (*e.g.*, *a-b* and *b-a*), measure pairs are ranked, with the lowest absolute difference receiving the lowest rank. Measure pairs whose subtraction equals 0 are assigned a 0 rank. Measure pairs with the same difference values are assigned the lowest rank. Third, ranks are assigned a sign using the sign of the measure differences calculated in the first step. Fourth, signed ranks for each subtraction type (*e.g.*, *a-b* and *b-a*) and paired measure set (*e.g.*, *x* and *y*) are aggregated. Fifth, bias indexes (BIs) are calculated as the sum of signed ranks divided by the maximum value that sum would have if all measure differences in the first set (*e.g.*, *x*) were greater than those in the second set (*e.g.*, *y*). Finally, the significance of observed BIs is determined by comparing them with a collection of expected-by-chance BIs calculated after repeatedly rearranging the measure matched-pair assortment between the two sets, and calculating *P_upper_* and *P_lower_* values as previously indicated. mbMCW tests can also follow two paths to calculate exact and approximated estimations of the significance of observed BIs as indicated for uMCW tests.

bMCW tests are a combination of two tests that assess whether a set of measures of bias for a quantitative trait between two conditions and a specific subset of these bias measures are significantly biased in the same direction. The bMCW testing process involves the following steps. First, all bias measures are ranked using their absolute values, with the lowest measure receiving the lowest rank. Second, ranks are assigned a sign using the sign of the bias measure under study. Third, a whole-set bias index (wBI) is calculated by summing signed ranks for all elements in the dataset and dividing it by the maximum number this sum could have if all bias measures were positive. Third, for each subset of elements in the dataset under study, a subset bias index (sBI) is calculated by summing signed ranks for the elements in the subset and dividing it by the maximum value this sum would have if the elements in the subset had the most positive values in the whole dataset. Fourth, the significance of observed wBIs is determined by comparing them with a collection of expected-by-chance wBIs calculated after repeatedly rearranging the signs of all signed ranks, and calculating *P_upper_* and *P_lower_* values as previously indicated. Finally, the significance of observed sBIs is determined by comparing them with a collection of expected-by-chance sBIs calculated after repeatedly rearranging the number of elements in the whole dataset that are associated with the subset under study, and calculating *P_upper_* and *P_lower_* values as previously indicated. bMCW tests can also follow two paths to calculate exact and approximated estimations of the significance of observed wBIs and sBIs as indicated for uMCW tests.

We used uMCW tests to determine whether mouse body weights, fasting glucose, tissue weights, plasma metabolite levels, litter size, sex ratio, and gene expression for individual genes were significantly biased in the same direction for each exposure group compared to controls (**Supplementary Data 21**, **23**). We used mbMCW tests to determine the significance of fasting body weights and water and food consumption for each exposure group compared to controls. For fasting body weights, we conducted mbMCW tests for mouse pre-fasting and post-fasting body weights data pairs for each experimental group (**Supplementary Data 22**). For water and food consumption, we conducted mbMCW tests for mouse cage entry and exit, and water and food data pairs for each experimental group. We used bMCW tests to determine whether gene expression differences for each exposure *versus* control contrast for genes in the whole transcriptome, overlapping specific isochores, overlapping isochores of the same class or located within autosomes and the *X* chromosome were significantly biased in the same direction (**Supplementary Data 25**, **27**). The measure for gene expression difference we used for bMCW tests was the uMCW test BIs obtained for each gene and exposure *versus* control contrast. For all MCW tests, we set *max_rearrangements* parameter to 10,000.

We used principal component analysis (PCA), gene set enrichment analysis (GSEA), and Pearson correlations, as previously described in the Metabolic and Transcriptomics section. We used the same threshold of significance of *P* = 0.05 for all analyses.

## Results

### Direct exposures to TBT, inorganic arsenic, and the Total Western Diet cause different metabolic disruptions

**Figure 1:**
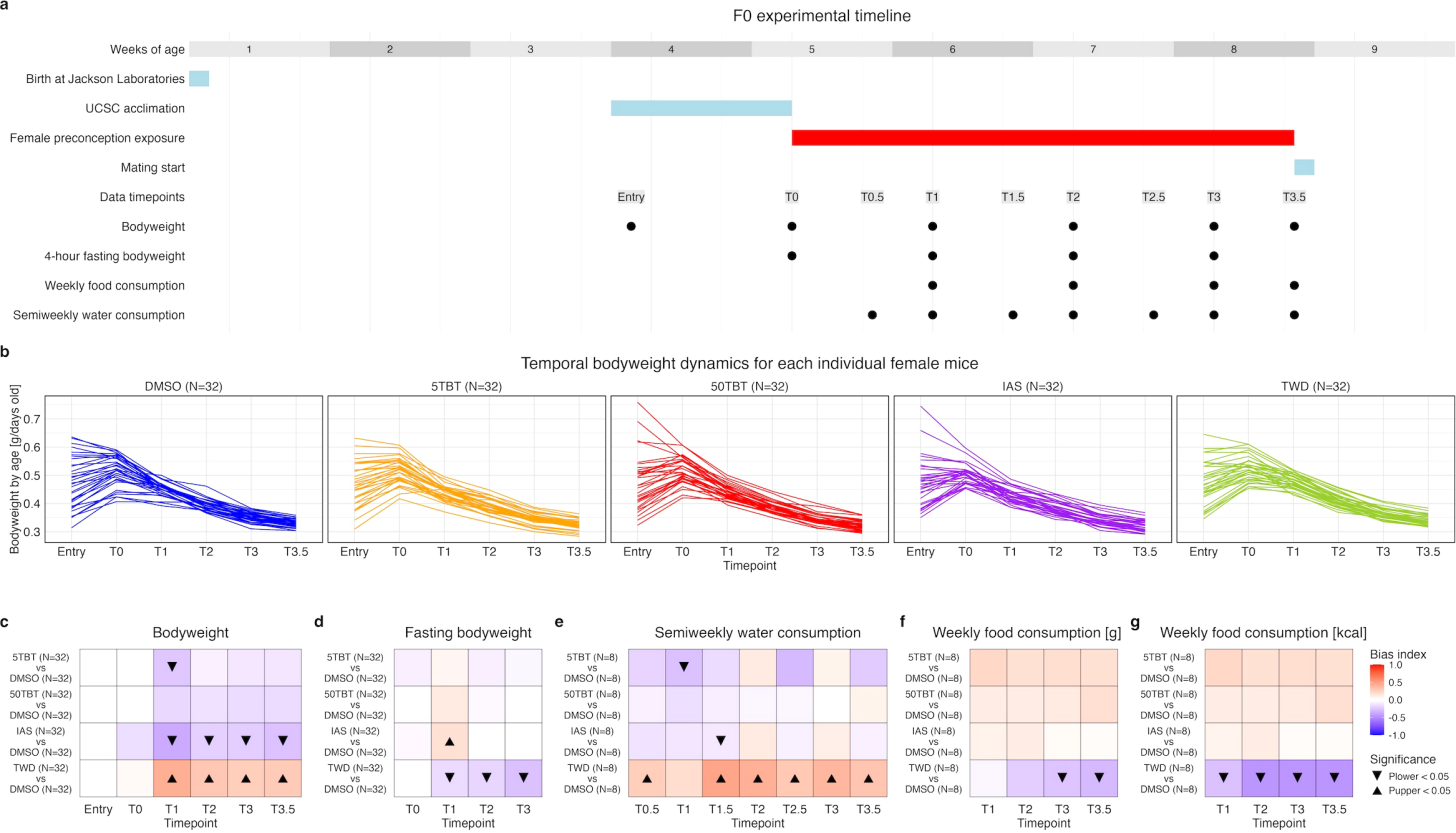
Direct effects of exposing F0 female mice to TBT, inorganic arsenic, and the Total Western Diet. **(a)** Experimental timeline detailing primary mouse operations for exposure to TBT, iAs, and TWD in female mice, and the timepoint structure used to assess variations in body weight, fasting body weight, water, and food consumption between exposure groups and controls. **(b)** Body weight dynamics for each mouse within each experimental group, from entry at UCSC Vivarium to timepoint T3.5. **(c)** Comparison of body weight normalized by age (g/days old) between exposure and control groups using uMCW tests. **(d)** Comparison of fasting body weight normalized by age (g/days old) between exposure and control groups using mbMCW tests. **(e)** Comparison of water consumption (mL) between exposure and control groups using mbMCW tests. **(f)** Comparison of food mass consumption between exposure and control groups using mbMCW tests. **(g)** Comparison of food caloric consumption (kcal) between exposure and control groups using mbMCW tests. Number of replicates (N) for each experimental group is indicated in each panel.

We randomly divided 160 female C57BL/6J mice into five groups. Each group received one of the following combinations of treated drinking water and diet for 3.5 weeks, starting at 5 weeks of age: DMSO and CD (negative control), 5 nM TBT and CD (5TBT group), 50 nM TBT and CD (50TBT group), 10 µg/L sodium (meta)arsenite and CD (IAS group), and DMSO and TWD (TWD group) (**Figure 1a**). We repeatedly measured body weight, fasting body weight, water, and food consumption to assess the efficacy of these exposures and determine if 5TBT, 50TBT, IAS, and TWD exposures caused metabolic disruptions compared to DMSO controls (**Figure 1**).

To determine whether body weights, fasting body weights, water consumption, and food consumption were significantly different between 5TBT, 50TBT, IAS, and TWD groups when compared with the control DMSO group, we used Monte Carlo-Wilcoxon (MCW) tests (see Methods for more details). MCW tests compute a bias index (BI) spanning 1 to -1 to quantify and determine the direction of bias between two conditions. To assess the statistical significance of the BIs computed for the user-provided dataset (observed BIs), a series of expected-by-chance BIs are generated by repeatedly rearranging the dataset and computing BIs for each iteration. *P_upper_* and *P_lower_* values are calculated as the proportions of expected-by-chance BIs that exhibit values equal to or higher than and equal to or less than the observed BIs, respectively. MCW tests include four different tests to interrogate different data structures. We used unmatched-measures MCW (uMCW) tests to determine whether mouse body weights were significantly biased in the same direction for each exposure group compared to controls (**Supplementary Data 21**). We used matched-measures bivariate MCW (mbMCW) tests to determine the significance of fasting body weights and water and food consumption for each exposure group compared to controls (**Supplementary Data 22**).

Although 5TBT, 50TBT, IAS, and TWD exposures appeared to disrupt the metabolism of exposed females, these disruptions manifested differently depending on the exposure. 5TBT exposure resulted in a significant reduction in body weight immediately after exposure, which was subsequently mitigated (**Figure 1c**). 50TBT exposure also resulted in a reduction in body weight, but such a difference was not significant at any timepoint (**Figure 1c**). No consistent patterns were observed for fasting body weight or water and food consumption (**Figure 1d**) for either TBT exposure. While TBT mice exhibited a tendency to consume more food and drink less water compared to DMSO controls (**Figures 1e–g**), water consumption was only significantly decreased for the 5TBT group in a single timepoint (**Figure 1e**).

IAS exposure also led to a substantial reduction in body weight immediately after exposure, which persisted throughout the entire exposure period (**Figure 1c**). Additionally, during the initial week of exposure, but not thereafter, fasting body weight in IAS females was significantly higher compared to DMSO controls (**Figure 1d**). This observation suggests that the reduction in body weight observed in IAS females may not have been equally responsive to the fasting challenge as the controls during the time when the body weight differences were most pronounced. Furthermore, IAS females appeared to consume more food and drink less than controls throughout the exposure period, although these differences were rarely significant (**Figures 1e–g**).

The group exhibiting the most pronounced and substantial differences was the TWD group. These female mice demonstrated significantly higher body weights, lower fasting body weights, increased water consumption, and decreased food consumption compared to controls throughout the exposure period (**Figures 1c-g**). The body weight excess in TWD mice is more readily mobilized even during a mild fasting regimen, which aligns with the substantial increase in water consumption observed in TWD mice when compared to controls (**Figures 1c–e**).

All these patterns are consistent with TBT and IAS exposures causing similar decreases in body weight, with the IAS group weight loss being more pronounced, and TWD exposure causing an increase in body weight. The fact that these four exposures lead to distinct metabolic disruptions provides a great opportunity to comparatively assess if they lead to distinctive metabolic disruptions in the offspring of exposed females.

### Preconception exposures of female mice to TBT, inorganic arsenic, and the Total Western Diet induce sexually dimorphic metabolic disruption in their offspring

**Figure 2:**
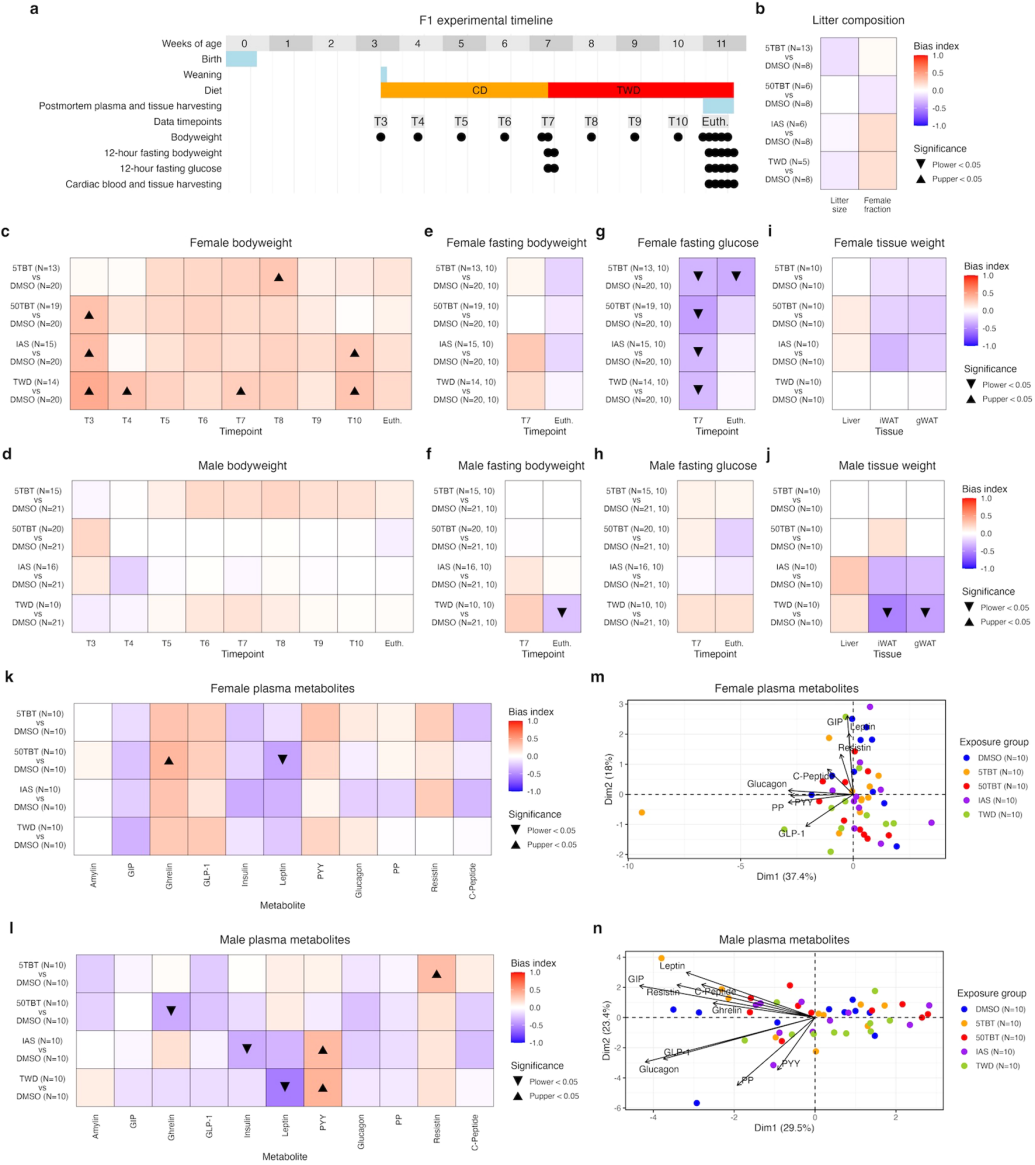
Effects of preconception exposure of F0 female mice to TBT, inorganic arsenic, and the Total Western Diet on the metabolism of their F1 offspring. **(a)** Experimental timeline detailing primary mouse operations and the timepoint structure used to assess variations in litter size, sex ratio, body weight, fasting body weight, fasting glucose, tissue weight, and plasma metabolite levels induced by maternal preconception exposure to TBT, IAS, and TWD compared to controls. **(b)** Comparison of litter size (N) and sex ratio (female fraction) between exposure and control groups using uMCW tests. **(c, d)**. Comparison of body weight normalized by age (g/days old) between exposure and control groups using uMCW tests for F1 females and males, respectively. **(e, f)**. Comparison of fasting body weight normalized by age (g/days old) between exposure and control groups using mbMCW tests for F1 females and males, respectively. **(g, h)**. Comparison of fasting glucose normalized by body weight and age ((mg/dL)/g/days old) between exposure and control groups using uMCW tests for F1 females and males, respectively. **(i, j)**. Comparison of tissue weight normalized by age (g/days old) between exposure and control groups using uMCW tests for F1 females and males, respectively. **(k, l)**. Comparison of plasma metabolite levels normalized by body weight and age ((pg/mL)/g/days old) between exposure and control groups using uMCW tests for F1 females and males, respectively. **(m, n)**. Principal component analyses (PCAs) of metabolite plasma levels normalized by body weight and age (pg/mL/g/days old) for females and males in exposure and control groups, respectively. Number of replicates (N) in each experimental group is indicated in each panel.

At 8 weeks of age and after 3.5 weeks of exposure, we mated F0 exposed females with same-age unexposed males for a week to produce their F1 progeny (**Figure 1a**, **2a**). At F1 weaning, we selected at least 10 mice for each sex and exposure group to determine if ancestral exposures resulted in F1 metabolic disruptions (**Figure 2**). We tracked the body weight of F1 mice weekly from weaning at 3 weeks of age until euthanasia at 11 weeks of age (**Figure 2**). In the past, we demonstrated that exposure to secondary metabolic challenges, such as a high-fat diet, could reveal metabolic disruptions caused by ancestral exposures (7, 9). We fed F1 mice CD since weaning until 7 weeks of age and TWD from then until the end of the experiment (**Figure 2**). At 7 weeks of age, when we changed diets, we fasted F1 mice for 12 hours and measured their body weight and glucose levels in peripheral blood (**Figure 2**). At 11 weeks of age, we fasted F1 mice for 12 hours before euthanasia and measured their body weight, glucose levels in peripheral blood, and the weight of gonadal and inguinal white adipose tissue depots (gWAT, and iWAT, respectively) and liver (**Figure 2**). We processed cardiac blood samples obtained during euthanasia to determine levels of 11 relevant metabolic regulators: amylin, GIP, ghrelin, GLP-1, insulin, leptin, PYY, glucagon, PP, resistin, and C-peptide.

We used uMCW tests to determine whether litter size, sex ratio, body weights, fasting glucose, tissue weights, and plasma metabolite levels were significantly biased in the same direction for each exposure group compared to controls (**Figures 2b–d, g-l**; **Supplementary Data 21**). We used mbMCW tests to determine whether fasting body weights were significantly biased in the same direction for each exposure group compared to controls (**Figures 2e, f**; **Supplementary Data 22**). To determine if the F1 groups were metabolically distinctive, we performed principal component analyses (PCA) using the plasma metabolite data (**Figures 2m, n**). To maximize the value of biological replication, we conducted PCA analyses using only metabolites whose plasma levels had been successfully determined in all biological replicates for all groups in each sex (8 in females and 9 in males).

We did not find any significant differences in litter size or sex ratio between the exposure groups and controls (**Figure 2b**). This suggests that the exposures did not cause any obvious negative effects on the fertility of the F0 females or the viability of their F1 offspring.

Distinct differences were observed between sexes in metabolic traits. Female groups of all treatments showed comparable signs of metabolic disruption compared to controls. They exhibited increased body weight, especially before weaning (**Figure 2c**), and had lower plasma glucose levels, especially before the diet change (**Figure 2g**). Similar alterations were observed in GIP, ghrelin, GLP-1, insulin, leptin, and PYY (**Figure 2k**). While other changes were not statistically significant or as pronounced, F1 females from the exposure groups exhibited similar trends compared to controls. For instance, they had higher and lower fasting body weight before the diet change and euthanasia, respectively (**Figure 2e**), and had reduced gWAT and iWAT weights compared to controls (**Figure 2i**). Although not all the trends or significant alterations were identical for the exposure groups, especially for the 5TBT group, the similarities between exposure groups were more pronounced than the differences.

F1 male groups showed less pronounced and more varied signs of metabolic disruption compared to controls. TWD mice exhibited more evident metabolic disruption, with lower fasting body weight, gWAT, and iWAT weights, and lower and higher plasma levels of leptin and PYY, respectively, at euthanasia (**Figures 2f, l**). TWD male mice were the only group with moderate differentiation from control male mice when analyzing plasma levels of nine metabolites using PCA (**Figure 2n**). IAS F1 males exhibited significantly higher and lower plasma levels of PYY and insulin, respectively (**Figure 2l**). IAS also exhibited non-significant lower trends in gWAT and iWAT weights and plasma levels of leptin at euthanasia, similar to the TWD group (**Figures 2j, l**). The two TBT groups exhibited less pronounced metabolic disruption. The only significant alterations in the 5TBT and 50TBT groups were in the plasma levels of resistin and ghrelin, respectively (**Figure 2l**).

### Preconception exposures of female mice to TBT, inorganic arsenic, and the Total Western Diet induce alterations in the expression of mitochondrial genes in their offspring

**Figure 3:**
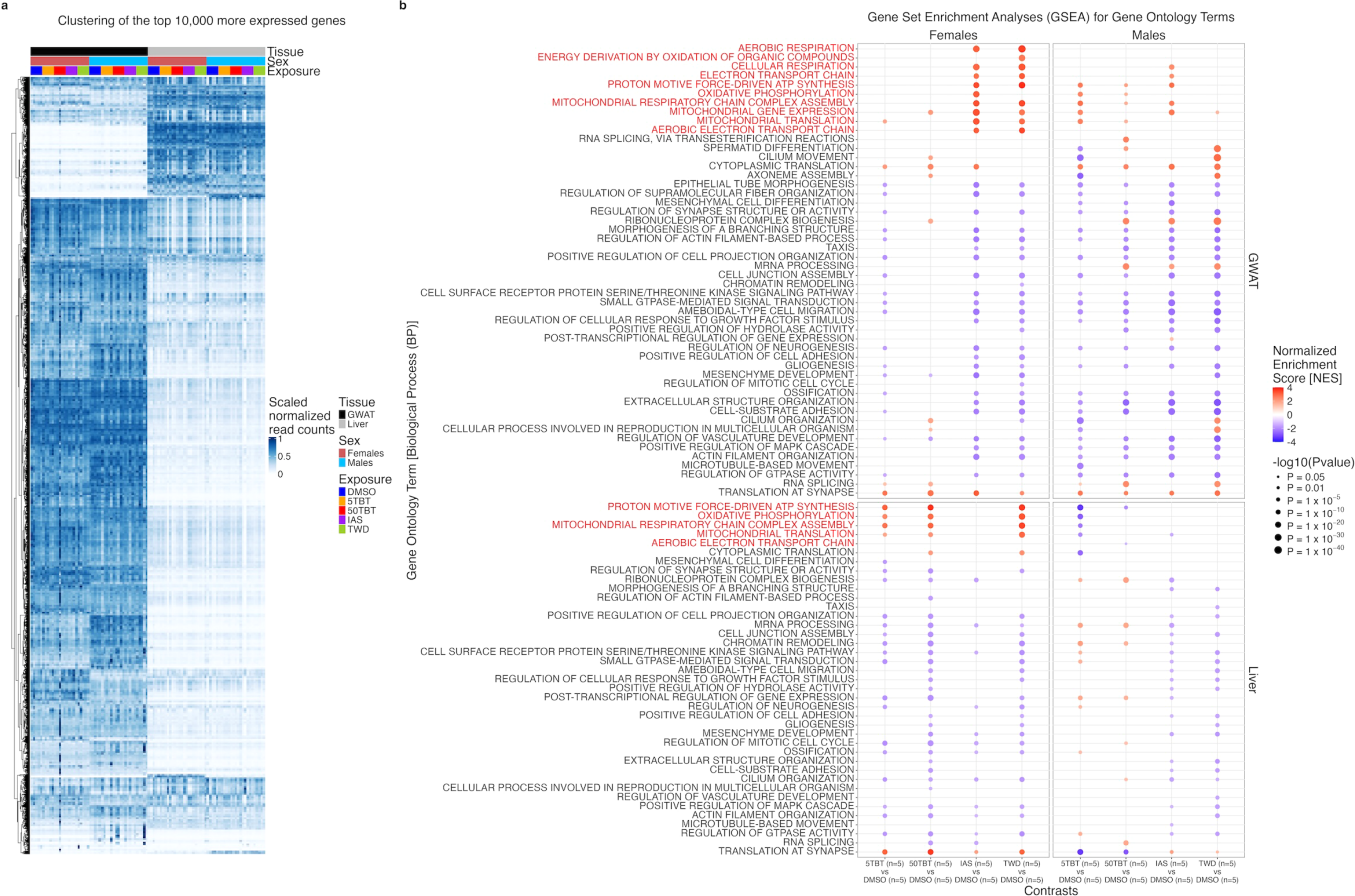
Effects of preconception exposure of F0 female mice to TBT, inorganic arsenic, and the Total Western Diet on the adipose and hepatic transcriptomes of their F1 offspring. **(a)** Gene expression patterns across 100 RNA samples (2 sexes x 2 tissues x 5 experimental groups x 5 replicates) for the 100,000 most expressed genes. **(b)** Gene Set Enrichment Analysis (GSEA) using Gene Ontology (GO) terms of Biological Process (BP) categories for all exposure *versus* control contrasts. For each gene and contrast, we conducted uMCW tests to assess the significance of the expression difference between exposure and control groups. Genes were ranked for each contrast using uMCW test BIs from highest to lowest, and GSEA analysis was performed using the R package *fgsea*. We present GSEA results for the 50 GO-BP with the most extreme GSEA *P* values across contrasts. GO-BP terms marked in red at the top of each panel correspond to metabolic GO-BP terms. Number of replicates (N) in each experimental group is indicated in the *x*-axis.

We processed gWAT and liver samples for transcriptomic analyses, as these tissues regulate energy homeostasis and previous studies showed that transcriptomic analysis can substantiate mechanisms underlying multigenerational metabolic disruptions induced by environmental exposures (7, 8). A general observation of the expression patterns of the 100,000 most expressed genes across all samples shows that their transcriptome is clearly distinctive between tissues and sexes, with the latter being more pronounced in gWAT than in liver (**Figure 3a**).

To elucidate gene expression alterations and their functional implications, we conducted uMCW tests for each gene across each of the 16 exposure *versus* control contrasts (2 sexes x 2 tissues x 4 exposure groups) (**Supplementary Data 23**). We then performed Gene Set Enrichment Analyses (GSEA) using Gene Ontology (GO) terms from the Biological Process (BP) category (**Supplementary Data 24**). **Figure 3b** presents GSEA results for GO-BP terms with the 50 highest -log10 (P values) across all contrasts. These findings show evident metabolic disruptions, consistent with mitochondrial function perturbation, with distinct differences observed between exposure groups and sexes. In females, mitochondrial GO-BP terms are overrepresented for genes with higher expression in gWAT in IAS and TWD groups compared to controls. These terms are also overrepresented for genes with higher expression in liver in 5TBT, 50TBT, and TWD groups relative to controls. In males, mitochondrial GO-BP terms are overrepresented for genes with higher expression in gWAT in 5TBT and IAS groups compared to controls, and for genes with lower expression in liver in 5TBT compared to controls.

The observed enrichment patterns in GO-BP suggest that the similar metabolic disruption in F1 female exposure groups (**Figure 2**) may be dependent on alterations in mitochondrial function (**Figure 3b**). However, there are distinctions. TBT groups show more significant mitochondrial alterations in the liver than gWAT, while the IAS group shows gWAT as the most affected tissue, and the TWD group shows mitochondrial alterations in both tissues.

The relationship between metabolic and transcriptomic alterations in males is less clear. The TWD group shows more distinct metabolic alterations compared to controls (**Figure 2**), with clear functional alterations in gWAT, but not mitochondrial function (**Figure 3b**). Conversely, the 5TBT and IAS groups, which show limited signs of metabolic disruption (**Figure 2**), demonstrate potential mitochondrial function alterations (**Figure 3b**).

### Preconception exposures of female mice to TBT, inorganic arsenic, and the Total Western Diet induce sexually dimorphic alterations of HEC in their offspring

Transcriptomic analyses, guided by indirect approximations of chromatin organization, have shown promise in identifying preliminary evidence of environmental exposure-induced chromatin organization perturbations in the descendants of exposed individuals (7, 8, 10). A hallmark of these perturbations was a dichotomous pattern of significant gene expression changes in heterochromatic and euchromatic compartments. The expression of genes located in heterochromatin was significantly biased in one direction, while the expression of genes located in euchromatin was significantly biased in the other direction (7, 8, 10).

**Figure 4:**
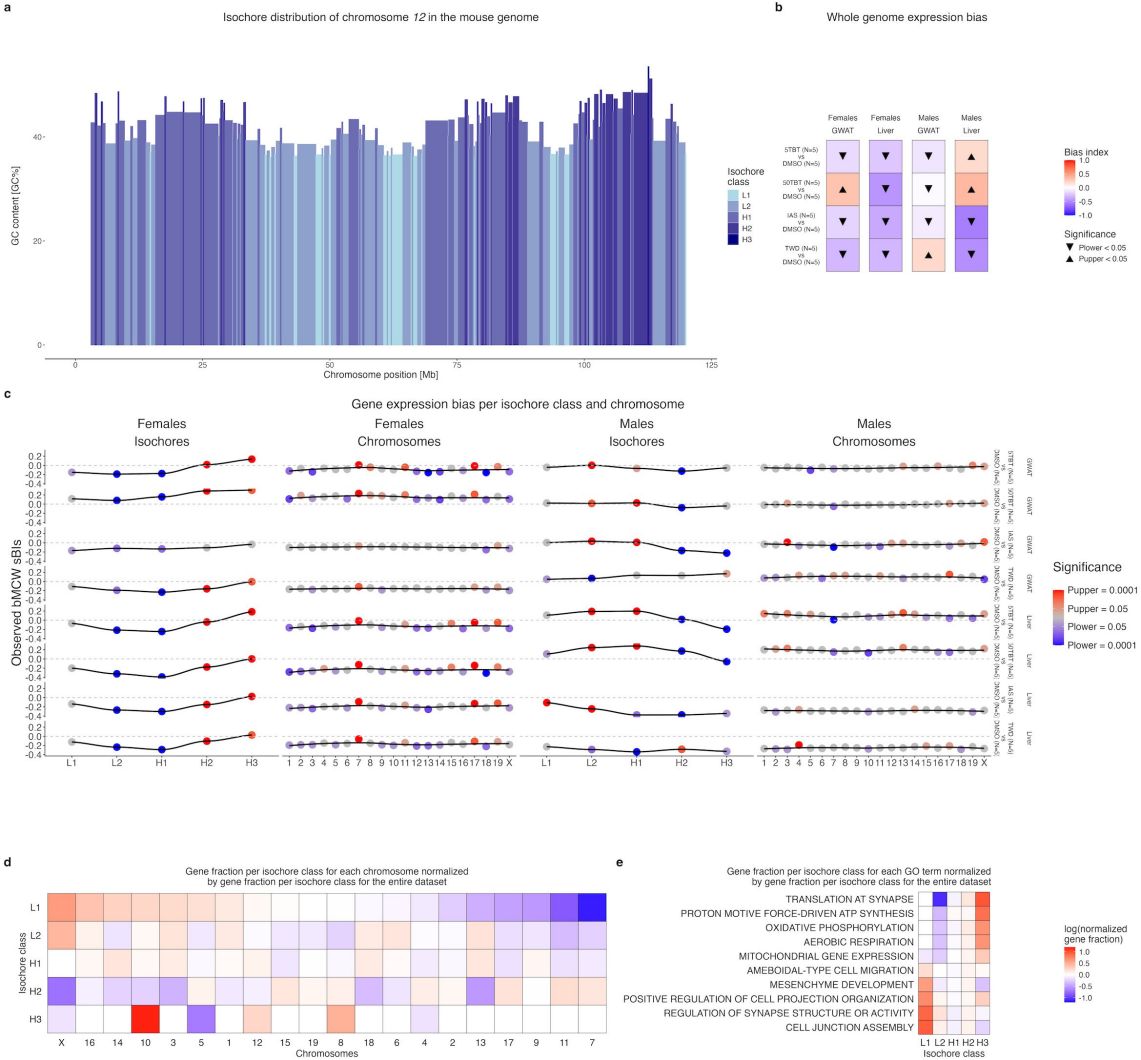
Isochore distribution of F1 adipose and hepatic transcriptome alterations induced by preconception exposure of F0 female mice to TBT, inorganic arsenic, and the Total Western Diet. **(a)** Isochore composition of mouse chromosome *12*. Isochore classes (L1, L2, H1, H2, and H3) are defined by GC content, from most AT-rich to most GC-rich. **(b)** Analysis of concerted alterations in gene expression for all genes in the transcriptome and each exposure *versus* control contrast using bMCW tests. **(c)** Analysis of concerted alterations in gene expression for each isochore class, autosome, and *X* chromosome in female and male gWAT and liver samples for all exposure *versus* control contrasts using bMCW tests. **(d)** Relative gene distribution within each isochore class for all autosomes and the *X* chromosome for genes in the female gWAT 50TBT *versus* control contrast. **(e)** Relative gene distribution within each isochore class for 10 GO-BP terms selected from **Figure 3b** for genes in the female gWAT 50TBT *versus* control contrast. bMCW sBIs: biased Monte Carlo-Wilcoxon test subset-Bias Indexes (see Methods).

To ascertain whether preconception exposure of female mice to TBT, IAS, and TWD resulted in comparable dichotomous perturbations of HEC, we employed an indirect approach we previously devised that utilizes isochores as approximations of varying chromatin organization levels (7, 8). Isochores are long segments of chromosomes characterized by a pronounced propensity for base composition uniformity, typically categorized into five classes: L1, L2, H1, H2, and H3, from most AT-rich to most GC-rich (59, 60) (**Figure 4a**). Isochores are relevant as approximations of chromatin organization for integrative analyses because: (i) their base composition is invariant across sexes, developmental stages, cell types, or individuals within the same species; (ii) individual isochores approximate well local chromatin structural motifs like topologically associating domains (TADs) (59, 60); and (iii) AT-rich L1 and L2 isochores and GC-rich H2 and H3 isochores effectively represent the heterochromatic and euchromatic compartments of eukaryotic genomes (59, 60).

We used biased-measures MCW (bMCW; see Methods section for more details) tests with data from F1 female and male mice, gWAT, and liver to determine whether genes located within AT- and GC-rich isochores exhibit concerted alterations in gene expression in response to preconception exposure of female mice to TBT, IAS, and TWD. We used bMCW tests to determine whether gene expression differences for each exposure *versus* control contrast for genes in the whole transcriptome, overlapping specific isochores, and overlapping isochores of the same class or located within autosomes and the *X* chromosome were significantly biased in the same direction. The measure for gene expression difference used in bMCW tests was the uMCW test BIs obtained for each gene and exposure *versus* control contrast (**Supplementary Data 24**). The analysis of concerted biases of expression for the whole transcriptome and at the chromosome level provided context to the results obtained using isochores. Since all chromosomes in vertebrates possess isochores belonging to the five classes (34, 61, 62), chromosomes represent an independent yet interrelated motive of chromatin organization distinct from the motives approximated by isochores.

Our isochore-based bMCW test results showed distinct sex differences comparable to those we observed for other metabolic traits (**Figure 2**; **Supplementary Data 25**). Specifically, all female groups exhibited similar patterns, whereas in the male groups, TWD stood out as the most distinctive from the other three.

In female gWAT, the transcriptome exhibits a general tendency to be overexpressed in 50TBT males and underexpressed in the other three groups compared to controls (**Figure 4b**). Despite these disparities, the significance of the expression biases for genes in AT- and GC-rich isochores reveals a similar dichotomous pattern across all four groups (**Figure 4c**). The bias in expression for genes situated in GC-rich isochores is higher than what would be expected by chance, while the bias in expression for genes in AT-rich isochores is lower than what would be expected by chance. In female liver, the patterns between groups are even more uniform. The transcriptome as a whole is underexpressed when compared to controls (**Figure 4b**). Additionally, the significance of the expression biases for genes in AT- and GC-rich isochores demonstrates the same dichotomous pattern between groups, closely resembling the pattern observed in gWAT (**Figure 4c**).

In male gWAT, groups 5TBT, 50TBT, and IAS exhibit similar patterns. The transcriptome as a whole tends to be underexpressed when compared to controls (**Figure 4b**). Furthermore, the significance of the biases in expression for genes in AT- and GC-rich isochores demonstrates the same dichotomous patterns, namely that gene expression bias for AT- and GC-rich isochores is higher and lower than what would be expected by chance, respectively (**Figure 4c**). These last patterns are the exact opposite of the ones observed for the females of the same groups in gWAT (**Figure 4c**). The patterns observed in male TWD gWAT clearly differ from the other three groups (**Figure 4c**).

In male liver, the heterogeneity between groups is more pronounced. In the TBT groups, the transcriptome as a whole tends to be overexpressed when compared to controls (**Figure 4b**). Additionally, the significance of the biases in expression for genes in AT- and GC-rich isochores is precisely the same as in male gWAT and the exact opposite of female liver of TBT groups (**Figure 4c**). In the case of IAS and TWD groups, the transcriptome as a whole tends to be underexpressed when compared to controls (**Figure 4b**). Regarding the significance of the biases in expression for genes in AT- and GC-rich isochores, the IAS group resembles more the patterns observed for TBT groups than the TWD group does (**Figure 4c**).

The chromosome-based bMCW tests also yield significant results, albeit with less pronounced bias values compared to their isochore-based bMCW test counterparts (**Figure 4c**). In fact, the results obtained for chromosomes can be attributed to their distinct isochore composition and the biases observed in each isochore class (**Figures 4c–d**, **Supplementary Figure 1**, and **Supplementary Data 26**). For instance, the expression of genes in chromosomes *7* and *11*, which contain more GC- than AT-rich isochores (**Figure 4d**; **Supplementary Figure 1**), tends to be biased in the same direction as GC-rich isochores do for each exposure *versus* control contrast (**Figure 4c**). Conversely, *X* and *3* chromosomes, which contain more AT- than GC-rich isochores (**Figure 4d**; **Supplementary Figure 1**), tend to be biased in the same direction as AT-rich isochores do for each exposure *versus* control contrast (**Figure 4c**).

The dichotomous patterns of gene expression bias observed in AT- and GC-rich isochores align with our hypothesis that preconception exposure to TBT and IAS may have resulted in sexually dimorphic HEC perturbations in their offspring. Conversely, while our findings for TWD also support HEC perturbations in the offspring of exposed females, such perturbations appear to be non-sexually dimorphic.

To determine whether such perturbations could be interrelated with the functional alterations previously observed (**Figure 3b**), we analyzed the isochore distribution of genes associated with some of the GO-BP with more pronounced results (**Figure 3b**; **Supplementary Data 26**). **Figure 4e** and **Supplementary Figure 2** illustrate the isochore distribution of genes associated with five GO-BP terms overrepresented in genes that were underexpressed in the exposure groups compared to controls, and five GO-BP terms overrepresented in genes that were overexpressed in the exposure groups (**Figure 3b**). Notably, the two sets of terms exhibit contrasting bias regarding the gene isochore distributions. Genes associated with GO-BP terms that were overrepresented for genes that were overexpressed in the exposure groups tend to be preferentially located in GC-rich isochores, while genes associated with GO-BP terms that were overrepresented for genes that were underexpressed in the exposure groups tend to be preferentially located in AT-rich isochores.

**Figure 5:**
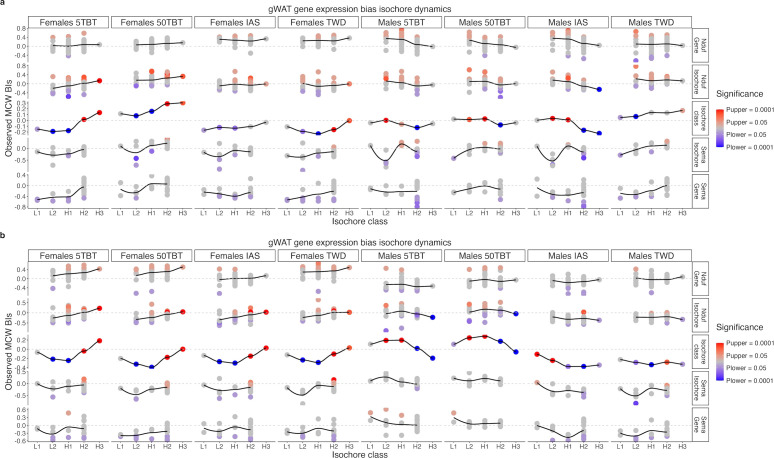
Concerted alterations of the F1 adipose and hepatic transcriptomes induced by preconception exposure of F0 female mice to TBT, inorganic arsenic, and the Total Western Diet. Analysis of gene expression bias for genes belonging to the Nduf and Sema gene families, the isochores where each of these genes reside, and each isochore class for each of the exposure *versus* control contrasts in gWAT **(a)** and liver **(b)**. Individual gene analyses were conducted using uMCW tests, while specific isochore and isochore class analyses were performed using bMCW tests. The results for isochore classes are also represented in **Figure 4c**. MCW BIs: Monte Carlo-Wilcoxon test Bias Indexes (see Methods).

These latter results suggest a potential interplay between HEC and functional perturbations induced by maternal preconception exposure to TBT, IAS, and TWD in their offspring’s gWAT and liver. To further explore this interplay, we compared gene expression biases for genes belonging to two large gene families with broad but distinct isochore distributions, the specific isochores where each member of these families is located, and the five isochore classes in gWAT and liver for all the exposure *versus* control contrasts (**Figure 5**; **Supplementary Data 20**, **27**).

We initially identified the Sema and Nduf gene families due to their distinct association with GO-BP terms overrepresented in over- and underexpressed genes across all the exposure *versus* control contrasts. The mitochondrial Complex I (NADH:ubiquinone oxidoreductase) is a protein complex that participates in the respiratory chain, with subunits encoded both in the mitochondrial and nuclear genomes (63). The Nduf gene family comprises all genes encoding nuclear subunits of Complex I. Nduf genes were associated with mitochondrial GO-BP terms such as “*Proton Motive Force-Driven ATP Synthesis*”, “*Oxidative Phosphorylation*”, and “*Aerobic Respiration*”, which were predominantly overrepresented in genes that were overexpressed in exposure groups compared to controls (**Figure 3b**). The Sema gene family encodes Semaphorins, a large family of surface receptors and ligands that regulate multiple processes in neuronal, vascular, immune, bone, and epithelial systems (64, 65). Sema genes were associated with GO-BP terms such as “*Ameboidal type cell migration*” and “*Mesenchyme development*”, which were mostly overrepresented in genes that were underexpressed in exposure groups compared to controls (**Figure 3b**).

Our transcriptomic dataset comprises 55 Nduf genes (including three pseudogenes) and 20 Sema genes (**Supplementary Data 20**). To ascertain whether the expression of genes and groups of genes located within the same isochore mirrored the patterns observed for isochore classes, we identified the isochores in which each Nduf and Sema gene was located (**Supplementary Data 20**). While both gene families are represented in four out of the five isochore classes, the Nduf isochore distribution exhibits a slight preference for GC-rich isochores, whereas the Sema isochore distribution exhibits a slight preference for AT-rich isochores (**Figure 5**; **Supplementary Data 20**). Subsequently, we conducted bMCW tests to determine if the expression of the genes contained within those isochores was altered in a concordant manner (**Supplementary Data 27**). **Figure 5** presents the outcomes of bMCW tests for each isochore class (also represented in **Figure 4c**), bMCW tests for specific isochores harboring Nduf or Sema genes, and uMCW tests for each Nduf and Sema gene.

Notably, despite some exceptions, the expression of individual Nduf and Sema genes and the specific isochores in which they reside appear to correspond with the dichotomous patterns observed for the five isochore classes. For instance, in female 5TBT liver, genes situated within GC-rich H2 and H3 isochores tend to be overexpressed, while genes located within AT-rich L1, L2, and H1 isochores tend to be underexpressed relative to controls (**Figure 5**). This dichotomy is replicated with varying degrees of intensity for Nduf and Sema genes and the specific isochores where each of these genes is situated.

In summary, our isochore-based analyses support the hypothesis that preconception exposure to TBT, IAS, and TWD results in HEC perturbations in their offspring, which are perceived at varying levels of resolution: specific genes, isochores, namely localized regions encompassing multiple genes defined by their base composition uniformity, and isochore classes, namely dispersed genomic regions characterized by similar base composition.

### Preconception exposures of female mice to TBT, inorganic arsenic, and the Total Western Diet might alter the chromatin architecture regulating the expression of leptin in females but not in males

To explore the link between the disruptions we observed in the descendants of female mice exposed to TBT, IAS, and TWD prior to conception for metabolic traits and chromatin organization, we focused on the gene *Lep*, which codes for leptin. Leptin, a hormone primarily secreted by adipose tissue, regulates feeding behaviors by signaling the hypothalamus about energy reserves (66, 67). When reserves are full, like after a meal, leptin secretion increases, informing the brain about satiety and preventing overeating. Recent research suggests that a chromosome loop involving neighboring genes *Lnclep* and *Lep* is crucial for regulating the expression of the leptin gene, and that the expression of *Lnclep* and *Lep* is strongly correlated across various pathophysiological conditions (68–70).

**Figure 6:**
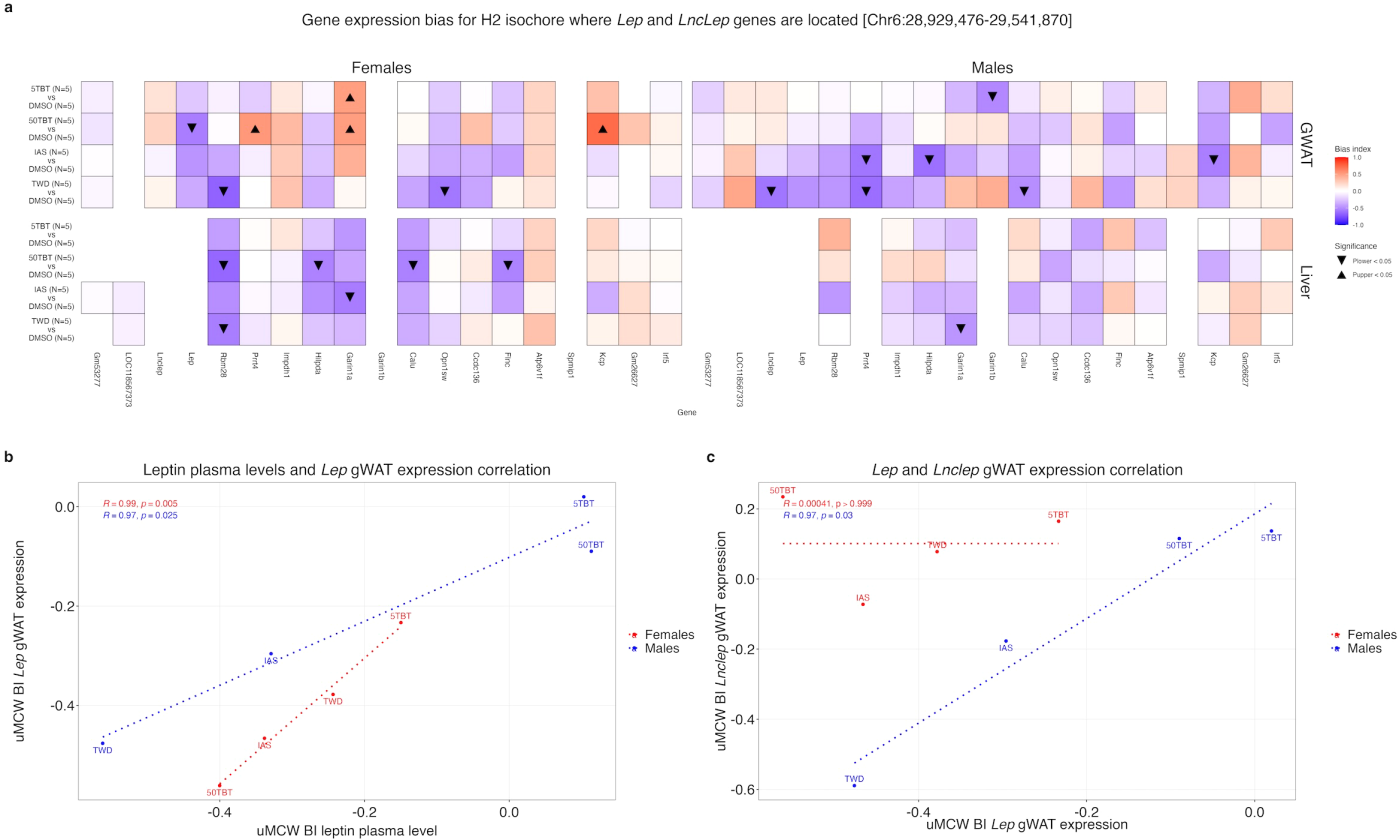
F1 leptin-related alterations induced by preconception exposure of F0 female mice to TBT, inorganic arsenic, and the Total Western Diet. **(a)** Analysis of gene expression bias for genes in the H2 isochore where the gene *Lep* is located using uMCW tests. Missing tiles indicate non-expressed genes (see Methods). **(b)** Pearson correlation analysis between changes in leptin plasma levels and *Lep* gene expression in gWAT for female and male exposure *versus* control contrasts determined using uMCW tests. **(c)** Pearson correlation analysis between *Lep* and *Lnclep* gene expression changes in gWAT for female and male exposure *versus* control contrasts determined using uMCW tests. uMCW BI: unmatched-measures Monte Carlo-Wilcoxon Bias Index.

We previously noted decreased plasma leptin levels in F1 females from the four exposure groups and in F1 males for the IAS and TWD groups (**Figures 2k–l**). Furthermore, leptin plasma levels were a significant metabolite contributing to the PCA separation of TBT, IAS, and TWD groups from controls in females and TWD from the other groups in males (**Figures 2m, n**). To elucidate whether female preconception exposure to TBT, IAS, and TWD induces alterations in the chromatin organization of the gene *Lep*, which may explain the observed leptin plasma levels changes, we identified the isochore where the gene *Lep* is located (**Supplementary Data 20**). **Figure 6a** shows the results of our uMCW tests for all genes within the same H2 isochore, encompassing the *Lep* and *Lnclep* genes, for the 16 exposure *versus* control contrasts. Notably, both the *Lep* and *Lnclep* genes are absent from liver tissue, consistent with the well-established adipose-specific expression of *Lep (*71).

**Figure 6b** shows the highly statistically significant correlations between decreases in plasma leptin levels and *Lep* gene expression in both female and male descendants of females exposed to TBT, IAS, and TWD compared to the control group. Since adipose tissue is the primary source of leptin secretion, these findings suggest that the declines in plasma leptin we observed are due to alterations in the expression of the *Lep* gene in the adipose tissue. Notably, our results demonstrating the absence of *Lep* expression in liver tissue and the precise correlations between variations in plasma leptin and the expression of the *Lep* gene serve as validation of our analytical approach in effectively capturing the adipose-specificity of the *Lep* gene.

**Figure 6c** shows the correlation between uMCW test results for *Lep* and *Lnclep* gene expression for female and male exposure *versus* control contrasts separately. In males, there is an almost perfect correlation between the changes in expression for both genes, which would agree with the afore-mentioned literature that suggests that the expression of these two genes is highly correlated because its regulation is dependent on the same secondary structure (68–70). However, in females, the changes in expression for both genes are not at all correlated. These results suggest that while the architectural regulatory context mediating the correlation of *Lep* and *Lnclep* expression in males is preserved, such context is perturbed in females whose mothers were exposed to TBT, IAS, or TWD.

## Discussion

In this study, we investigated whether preconception exposure of female mice to three metabolic disruptors induced metabolic alterations in their offspring that could be mediated by perturbations of chromatin organization. Our findings generally support this scenario, but also present unexpected results. If we only considered the effects observed in exposed females and their male offspring, we would conclude each exposure had different effects, with TWD exposure being the most pronounced and distinctive.

Contrary to the direct and male offspring effects, the results for female offspring are surprising. Firstly, the phenotypes of the four experimental groups compared to controls exhibited more similarities than differences (**Figures 2**–**5**). Secondly, the perturbations in offspring females exhibit remarkable similarities to the *atypical anorexia nervosa* condition observed in humans. We observed low plasma leptin, insulin, and glucose, high plasma ghrelin, GLP-1, and PYY, and low adipose tissue weight in TBT, IAS, and TWD females compared to controls (**Figure 2**). Although many of these alterations would not be considered significant at the commonly used threshold of significance (*P* = 0.05), it is remarkable that all such trends have been observed in anorexic human females (72–77). Moreover, altered mitochondrial functions, such as those we observed in gWAT and liver transcriptomic analyses (**Figure 3b**), have been reported in studies of transcriptome variation between anorexic patients and healthy controls (78).

Despite aligning with anorexic phenotypes, it is challenging to definitively determine if female offspring of female mice exposed to TBT, IAs, or TWD exhibit anorexia, especially since self-starvation and substantial weight loss are the primary diagnostic characteristics associated with such conditions (79). Firstly, we lack valid data on the feeding behavior of offspring females in our study. We did not conduct individualized measurements of food consumption, and the number of cage replicates per experimental group was limited. Secondly, we did not observe significant or consistent variation trends in body weight in response to fasting for TBT, IAs, or TWD females compared to controls (**Figure 2e**). Thirdly, females in the TBT, IAS, and TWD groups tended to be overweighted compared to controls, with varying degrees of significance across the study period (**Figure 2c**). *Atypical anorexia nervosa* is used to describe patients who exhibit signs of anorexia but do not experience a decrease in body weight (79). While further characterization of offspring effects induced by preconception exposure to metabolism disruptors is warranted, it is intriguing to speculate that such exposures may predispose to *atypical anorexia nervosa*. Despite the growing recognition of the significance of metabolic alterations in the pathogenesis and manifestation of *anorexia nervosa (*80, 81), and the availability of numerous animal models for the study of *anorexia nervosa (*82), to the best of our knowledge, no empirical evidence has been found establishing a causal relationship between anorexic phenotypes and exposure to metabolism disruptors.

A distinctive characteristic of our study is its high degree of integration of disparate data. Our study yielded data on multiple metabolic traits, both basally and in response to metabolic challenges like fasting or diet, as well as transcriptomic data from two F1 sexes and two tissues with distinct ontogenies. Three key factors facilitated this integration.

Firstly, a standardized methodology was used to compare data from exposure groups and controls for all traits. The fact that various MCW tests, each interrogating different data structures and traits, yielded comparable metrics such as BIs, *P_upper_*, and *P_lower_* significantly contributed to establishing connections between different traits. The correlations observed between uMCW test results for plasma leptin levels and *Lep* gene expression in gWAT (**Figure 6b**) and the similarities observed between uMCW test results for plasma metabolite levels and the weight of such levels in PCA (**Figures 2k-n**) demonstrate the utility of MCW tests for the integrative analysis of multidimensional data in complex studies.

Secondly, we used non-stringent definitions of statistical significance. While our study aimed to find evidence consistent with a hypothesis, it is inherently exploratory in nature. While distinguishing true and false positives is often given special attention, such as through multiple test corrections, it is equally perilous for exploratory studies to discard potentially true positives by employing a highly stringent threshold of significance, which could result in false negatives. To determine potentially significant results, we integrated multidimensional data using the same analytical approach, MCW tests, and distinct approaches to measure the same trait at different levels, *such as* leptin plasma levels and gWAT expression (**Figure 5**) or gene expression biases for specific genes, isochores, and isochores of the same class (**Figure 4**). In the case of plasma levels of metabolites, we observed similar patterns in females even when these metabolites were not considered significant using conventional non-stringent definition of significance (*P* = 0.05), that resemble the weight of such metabolites in PCAs (**Figure 2**).

Lastly, although we did not intend originally to study exposures that were expected to yield highly similar outcomes in the female offspring of exposed females, this fact contributed to the identification of potentially relevant results. The comparable trends in plasma metabolites and body weight among the four exposure groups compared to controls suggest a potential link between female preconception exposure to metabolic disruptors and an epigenetic predisposition to *atypical anorexia nervosa* in their female offspring.

Despite its strengths, our study also has limitations. Our central hypothesis posited that female preconception exposure to metabolic disruptors could lead to offspring metabolic alterations mediated by changes in the germ line of exposed individuals. These changes, in turn, could affect the offspring’s establishment of chromatin organization, which has the potential to self-propagate throughout development and predispose to metabolic disruption. Nevertheless, we cannot entirely dismiss the possibility that the effects we observed were influenced by the direct action of exposure agents, such as TBT, IAS, or components of TWD, or the results of their metabolization, like dibutyltin (DBT) in the case of TBT (83), persisted or bioaccumulated in the mother after the interruption of the exposure immediately prior to fertilization on the developing embryo. Additionally, the influence of alterations caused by the exposures in the mother during the development of the embryo could also contribute to the effects we aimed to study and their direction. Future studies based on the *in vitro* fertilization of oocytes from exposed females are needed to disentangle the potential contribution of the effects of environmental exposure on germ cells of the exposed individual, the developing embryo, and/or the physiological alterations of the mother during gestation.

Our analyses, which aimed to identify disruptions in metabolism and chromatin organization, were neither comprehensive nor direct. This was primarily due to our commitment to utilizing widely accessible methodologies, such as standard balances, measure cylinders, facility-provided bulk transcriptomic analyses, and publicly available computer tools. The intentionally low resolution imposed limitations on our ability to, for instance, capture feeding behaviors that contributed to substantiate the apparent anorexic condition of the female descendance of mothers exposed to metabolism disruptors. A more comprehensive and intricate battery of determinations is needed to fully comprehend the metabolic alterations caused by the preconception exposure of females to metabolic disruptors. Also, although our isochore-based transcriptomic analyses can only provide indirect evidence of chromatin organization perturbations, methodologies that directly interrogate chromatin organization, such as high-throughput chromosome conformation capture (Hi-C), remain experimentally and analytically demanding, and their application in complex studies and tissues remains cumbersome (84).

Our study explores the interplay between two largely unexplored elements: the susceptibility window before conception for the effects of metabolism disruptors that can be propagated across generations, and the susceptibility of chromatin organization to the effects of environmental exposures in a metastable manner. Consequently, the availability of pertinent literature to contextualize the findings of our study is limited. The relatively recent implementation of methodologies to interrogate the complexity of the three-dimensional layout and compartmentalization of nuclear genomes explains the limited knowledge on the susceptibility of such levels of organization to accommodate the effects of environmental exposures. Additionally, the prevailing perspective in the study of long-lasting effects of environmental exposures suggests that these effects are induced by factors that perturb normal prenatal and infancy development. While exposure paradigms often include preconception, the number of studies focusing exclusively on this period, especially female preconception, is limited.

## Conclusion

This study provides support for the hypothesis that preconception exposure to metabolic disruptors can cause metabolic alterations in the offspring of exposed individuals through perturbations of chromatin organization. Although our study has the low analytical resolution typical of preliminary exploratory studies, it is noteworthy the level of congruence observed after integrating multidimensional data. This validates our approach as a preliminary step towards determining the potential of environmental exposures to lead to multigenerational effects. Beyond the methodological value of our study, the potential connection we uncovered between preconception exposure to metabolic disruptors and *atypical anorexia nervosa* presents a new avenue for determining the environmental component of complex chronic metabolic or metabolically related diseases beyond the usual suspects of obesity and type 2 diabetes. In addition, the findings of this study provide a roadmap for investigating how the sustained application of our murine preconception exposure model in response to a wide range of metabolism disruptors can yield valuable insights into whether disparate exposures result in comparable offspring outcomes through analogous mechanisms or whether eukaryotic epigenomes possess the ability to transduce signals elicited by distinct exposures into functionally equivalent altered states.

## Data Availability

The data and code necessary to replicate the analyses presented in this study are accessible in the **Supplementary Material** and public repositories. The transcriptomic data discussed herein have been deposited in NCBI’s Gene Expression Omnibus (GSE324852; https://www.ncbi.nlm.nih.gov/geo/query/acc.cgi?acc=GSE324852). The R code required to conduct analyses and reproduce the figures presented in this publication is publicly available in the GitHub repository https://github.com/diazcastillo/2026_Diaz-Castillo-et-al_Frontiers.

## References

[1] Gluckman PD Hanson MA . Living with the past: evolution, development, and patterns of disease. Science. (2004) 305:1733–6. doi: 10.1126/science.1095292 15375258

[2] Locke AE Kahali B Berndt SI Justice AE Pers TH Day FR . Genetic studies of body mass index yield new insights for obesity biology. Nature. (2015) 518:197–206. doi: 10.1038/nature14177 25673413 PMC4382211

[3] King SE Skinner MK . Epigenetic transgenerational inheritance of obesity susceptibility. Trends Endocrinol Metab. (2020) 31:478–94. doi: 10.1016/j.tem.2020.02.009, PMID: 32521235 PMC8260009

[4] Merrill MAL Smith MT McHale CM Heindel JJ Atlas E Cave MC . Consensus on the key characteristics of metabolism disruptors. Nat Rev Endocrinol. (2025) 21:245–61. doi: 10.1038/s41574-024-01059-8, PMID: 39613954 PMC11916920

[5] Klibaner-Schiff E Simonin EM Akdis CA Cheong A Johnson MM Karagas MR . Environmental exposures influence multigenerational epigenetic transmission. Clin Epigenet. (2024) 16:145. doi: 10.1186/s13148-024-01762-3, PMID: 39420431 PMC11487774

[6] Chamorro-García R Sahu M Abbey RJ Laude J Pham N Blumberg B . Transgenerational inheritance of increased fat depot size, stem cell reprogramming, and hepatic steatosis elicited by prenatal exposure to the obesogen tributyltin in mice. Environ Heal Perspect. (2013) 121:359–66. doi: 10.1289/ehp.1205701 PMC3621201 23322813

[7] Chamorro-Garcia R Diaz-Castillo C Shoucri BM Käch H Leavitt R Shioda T . Ancestral perinatal obesogen exposure results in a transgenerational thrifty phenotype in mice. Nat Commun. (2017) 8:2012. doi: 10.1038/s41467-017-01944-z, PMID: 29222412 PMC5722856

[8] Diaz-Castillo C Chamorro-Garcia R Shioda T Blumberg B . Transgenerational self-reconstruction of disrupted chromatin organization after exposure to an environmental stressor in mice. Sci Rep. (2019) 9:13057. doi: 10.1038/s41598-019-49440-2, PMID: 31506492 PMC6736928

[9] Chamorro-García R Poupin N Tremblay-Franco M Canlet C Egusquiza R Gautier R . Transgenerational metabolomic fingerprints in mice ancestrally exposed to the obesogen TBT. Environ Int. (2021) 157:106822. doi: 10.1016/j.envint.2021.106822, PMID: 34455191 PMC8919592

[10] Öst A Lempradl A Casas E Weigert M Tiko T Deniz M . Paternal diet defines offspring chromatin state and intergenerational obesity. Cell. (2014) 159:1352–64. doi: 10.1016/j.cell.2014.11.005, PMID: 25480298

[11] Bhatnagar A Karnay AM Elefant F . Chapter 13 - Drosophila epigenetics. In: Tollefsbol TO , editor. Handbook of epigenetics (2022). p. 215–47. doi: 10.1016/b978-0-323-91909-8.00015-3, PMID:

[12] Steel A Lee WR Xu Z Carè J Cortes-Ramirez J Thomson-Casey C . The influence of environmental exposures during the preconception period on offspring outcomes: a systematic review. Front Public Heal. (2025) 13:1633266. doi: 10.3389/fpubh.2025.1633266 PMC12753933 41480069

[13] Pecori F Torres-Padilla M-E . Dynamics of nuclear architecture during early embryonic development and lessons from liveimaging. Dev Cell. (2023) 58:435–49. doi: 10.1016/j.devcel.2023.02.018, PMID: 36977375 PMC10062924

[14] Rang FJ Kind J Guerreiro I . The role of heterochromatin in 3D genome organization during preimplantation development. Cell Rep. (2023) 42:112248. doi: 10.1016/j.celrep.2023.112248 37059092

[15] Bughio F Huckell GR Maggert KA . Monitoring of switches in heterochromatin-induced silencing shows incomplete establishment and developmental instabilities. Proc Natl Acad Sci. (2019) 116:20043–53. doi: 10.1073/pnas.1909724116, PMID: 31527269 PMC6778184

[16] Owen JA Osmanović D Mirny L . Design principles of 3D epigenetic memory systems. Science. (2023) 382:eadg3053. doi: 10.1126/science.adg3053 37972190 PMC11075759

[17] Kojima ML Hoppe C Giraldez AJ . The maternal-to-zygotic transition: reprogramming of the cytoplasm and nucleus. Nat Rev Genet. (2025) 26:245–67. doi: 10.1038/s41576-024-00792-0 PMC11928286 39587307

[18] Painter RC Roseboom TJ Bleker OP . Prenatal exposure to the Dutch famine and disease in later life: An overview. Reprod Toxicol. (2005) 20:345–52. doi: 10.1016/j.reprotox.2005.04.005, PMID: 15893910

[19] Golding J Gregory S Northstone K Pembrey M Watkins S Iles-Caven Y . Human transgenerational observations of regular smoking before puberty on fat mass in grandchildren and great-grandchildren. Sci Rep. (2022) 12:1139. doi: 10.1038/s41598-021-04504-0 35064168 PMC8782898

[20] Kaati G Bygren L Edvinsson S . Cardiovascular and diabetes mortality determined by nutrition during parents’ and grandparents’ slow growth period. Eur J Hum Genet. (2002) 10:682–8. doi: 10.1038/sj.ejhg.5200859, PMID: 12404098

[21] Vermeulen R Schymanski EL Barabási A-L Miller GW . The exposome and health: Where chemistry meets biology. Science. (2020) 367:392–6. doi: 10.1126/science.aay3164, PMID: 31974245 PMC7227413

[22] Uc-Peraza RG Castro ÍB Fillmann G . An absurd scenario in 2021: Banned TBT-based antifouling products still available on the market. Sci Total Environ. (2022) 805:150377. doi: 10.1016/j.scitotenv.2021.150377, PMID: 34818813

[23] Podgorski J Berg M . Global threat of arsenic in groundwater. Science. (2020) 368:845–50. doi: 10.1126/science.aba1510, PMID: 32439786

[24] Dye CK Domingo-Relloso A Kupsco A Tinkelman NE Spratlen MJ Bozack AK . Maternal DNA methylation signatures of Arsenic exposure is associated with adult offspring insulin resistance in the Strong Heart Study. Environ Int. (2023) 173:107774. doi: 10.1016/j.envint.2023.107774, PMID: 36805808 PMC10166110

[25] Shang B Venkatratnam A Liu T Douillet C Shi Q Miller M . Sex-specific transgenerational effects of preconception exposure to arsenite: metabolic phenotypes of C57BL/6 offspring. Arch Toxicol. (2023) 97:2879–92. doi: 10.1007/s00204-023-03582-5 PMC10754030 37615676

[26] Hintze KJ Benninghoff AD Ward RE . Formulation of the total western diet (TWD) as a basal diet for rodent cancer studies. J Agric Food Chem. (2012) 60:6736–42. doi: 10.1021/jf204509a 22224871

[27] Harper C Llados F . Toxicological profile for tin and tin compounds (2005). Available online at: https://stacks.cdc.gov/view/cdc/7003 (Accessed May 1, 2025).

[28] Chou C-HSJ Harper C . Toxicological profile for arsenic (2007). Available online at: https://stacks.cdc.gov/view/cdc/11481.

[29] R Core Team . R: A language and environment for statistical computing (2025). Available online at: https://www.R-project.org/.

[30] Kassambara A Mundt F . factoextra: extract and visualize the results of multivariate data analyses (2020). Available online at: https://CRAN.R-project.org/package=factoextra.

[31] Perez G Barber GP Benet-Pages A Casper J Clawson H Diekhans M . The UCSC Genome Browser database: 2025 update. Nucleic Acids Res. (2024) 53:D1243–9. doi: 10.1093/nar/gkae974 PMC11701590 39460617

[32] Aleksander SA Balhoff J Carbon S Cherry JM Drabkin HJ Ebert D . The gene ontology knowledgebase in 2023. GENETICS. (2023) 224:iyad031. doi: 10.1093/genetics/iyad031, PMID: 36866529 PMC10158837

[33] Ashburner M Ball CA Blake JA Botstein D Butler H Cherry JM . Gene Ontology: tool for the unification of biology. Nat Genet. (2000) 25:25–9. doi: 10.1038/75556, PMID: 10802651 PMC3037419

[34] Cozzi P Milanesi L Bernardi G . Segmenting the human genome into isochores. Evolutionary Bioinformatics (2015) 11:S27693. doi: 10.4137/EBO.S27693 PMC4662427 26640363

[35] The Galaxy Community Abueg LAL Afgan E Allart O Awan AH Bacon WA . The Galaxy platform for accessible, reproducible, and collaborative data analyses: 2024 update. Nucleic Acids Res. (2024) 52:W83–94. doi: 10.1093/nar/gkae410, PMID: 38769056 PMC11223835

[36] Ewels P Magnusson M Lundin S Käller M . MultiQC: summarize analysis results for multiple tools and samples in a single report. Bioinformatics. (2016) 32:3047–8. doi: 10.1093/bioinformatics/btw354 PMC5039924 27312411

[37] Martin M . Cutadapt removes adapter sequences from high-throughput sequencing reads. EMBnetJ. (2011) 17:10–2. doi: 10.14806/ej.17.1.200

[38] Dobin A Davis CA Schlesinger F Drenkow J Zaleski C Jha S . STAR: ultrafast universal RNA-seq aligner. Bioinformatics. (2013) 29:15–21. doi: 10.1093/bioinformatics/bts635, PMID: 23104886 PMC3530905

[39] Liao Y Smyth GK Shi W . featureCounts: an efficient general purpose program for assigning sequence reads to genomic features. Bioinformatics. (2014) 30:923–30. doi: 10.1093/bioinformatics/btt656, PMID: 24227677

[40] Posit Team . RStudio: integrated development environment for R (2025). Available online at: http://www.posit.co/.

[41] Barrett T Dowle M Srinivasan A Gorecki J Chirico M Hocking T . data.table: extension of `data.frame` (2025). Available online at: https://CRAN.R-project.org/package=data.table.

[42] Gu Z Eils R Schlesner M . Complex heatmaps reveal patterns and correlations in multidimensional genomic data. Bioinformatics. (2016) 32:2847–9. doi: 10.1093/bioinformatics/btw313, PMID: 27207943

[43] Subramanian A Tamayo P Mootha VK Mukherjee S Ebert BL Gillette MA . Gene set enrichment analysis: A knowledge-based approach for interpreting genome-wide expression profiles. Proc Natl Acad Sci. (2005) 102:15545–50. doi: 10.1073/pnas.0506580102, PMID: 16199517 PMC1239896

[44] Korotkevich G Sukhov V Budin N Shpak B Artyomov MN Sergushichev A . Fast gene set enrichment analysis. bioRxiv. (2021), 060012. doi: 10.1101/060012

[45] Dolgalev I . msigdbr: MSigDB gene sets for multiple organisms in a tidy data format. (2025). doi: 10.32614/cran.package.msigdbr. PMID:

[46] Min SH . Visualization of composite plots in R using a programmatic approach and smplot2. Adv Methods Pr Psychol Sci. (2024) 7:25152459241267927. doi: 10.1177/25152459241267927

[47] Wickham H . ggplot2: elegant graphics for data analysis (2016). Available online at: https://ggplot2.tidyverse.org.

[48] Yu G . ggplotify: convert plot to “grob” or “ggplot” Object. (2023). doi: 10.32614/cran.package.ggplotify. PMID:

[49] Slowikowski K . ggrepel: automatically position non-overlapping text labels with “ggplot2”. (2024). doi: 10.32614/cran.package.ggrepel. PMID:

[50] Wilke CO Wiernik BM . ggtext: improved text rendering support for “ggplot2”. (2022). doi: 10.32614/cran.package.ggtext. PMID:

[51] Pedersen TL . patchwork: the composer of plots. (2024). doi: 10.32614/cran.package.patchwork. PMID:

[52] Neuwirth E . RColorBrewer: colorBrewer palettes. (2022). doi: 10.32614/cran.package.rcolorbrewer.

[53] Wickham H Pedersen TL Seidel D . scales: scale functions for visualization. (2023). doi: 10.32614/cran.package.scales.

[54] Wickham H Henry L Pedersen TL Luciani TJ Decorde M Lise V . svglite: an “SVG” Graphics device. (2023). doi: 10.32614/cran.package.svglite.

[55] Díaz-Castillo C . Females and males contribute in opposite ways to the evolution of gene order in drosophila. PLoS One. (2013) 8:e64491. doi: 10.1371/journal.pone.0064491, PMID: 23696898 PMC3655977

[56] Díaz-Castillo C . Transcriptome dynamics along axolotl regenerative development are consistent with an extensive reduction in gene expression heterogeneity in dedifferentiated cells. PeerJ. (2017) 5:e4004. doi: 10.7717/peerj.4004 29134148 PMC5678507

[57] Díaz-Castillo C . Same-sex twin pair phenotypic correlations are consistent with human Y chromosome promoting phenotypic heterogeneity. Evol Biol. (2018) 45:248–58. doi: 10.1007/s11692-018-9454-y

[58] Diaz-Castillo C Chamorro-Garcia R . MCWtests: A suite of Monte Carlo-Wilcoxon tests (2025). Available online at: https://github.com/diazcastillo/MCWtests.

[59] Jabbari K Bernardi G . An isochore framework underlies chromatin architecture. PLoS One. (2017) 12:e0168023. doi: 10.1371/journal.pone.0168023, PMID: 28060840 PMC5218411

[60] Bernardi G . Chromosome architecture and genome organization. PLoS One. (2015) 10:e0143739. doi: 10.1371/journal.pone.0143739, PMID: 26619076 PMC4664426

[61] Costantini M Cammarano R Bernardi G . The evolution of isochore patterns in vertebrate genomes. BMC Genom. (2009) 10:1. doi: 10.1186/1471-2164-10-146, PMID: 19344507 PMC2678159

[62] Costantini M Clay O Auletta F Bernardi G . An isochore map of human chromosomes. Genome Res. (2006) 16:536–541. doi: 10.1101/gr.4910606, PMID: 16597586 PMC1457033

[63] Vinothkumar KR Zhu J Hirst J . Architecture of mammalian respiratory complex I. Nature. (2014) 515:80–4. doi: 10.1038/nature13686, PMID: 25209663 PMC4224586

[64] Alves CJ Yotoko K Zou H Friedel RH . Origin and evolution of plexins, semaphorins, and Met receptor tyrosine kinases. Sci Rep. (2019) 9:1970. doi: 10.1038/s41598-019-38512-y, PMID: 30760850 PMC6374515

[65] Goodman CS Kolodkin AL Luo Y Püschel AW Raper JA . Unified nomenclature for the semaphorins/collapsins. Cell. (1999) 97:551–2. doi: 10.1016/s0092-8674(00)80766-7, PMID: 10367884

[66] Friedman JM . Leptin and the endocrine control of energy balance. Nat Metab. (2019) 1:754–64. doi: 10.1038/s42255-019-0095-y, PMID: 32694767

[67] Friedman J . The long road to leptin. J Clin Investig. (2016) 126:4727–34. doi: 10.1172/jci91578, PMID: 27906690 PMC5127673

[68] Lo KA Huang S Walet ACE Zhang Z Leow MK-S Liu M . Adipocyte long-noncoding RNA transcriptome analysis of obese mice identified lnc-leptin, which regulates leptin. Diabetes. (2018) 67:1045–56. doi: 10.2337/db17-0526, PMID: 29519872

[69] Dallner OS Marinis JM Y-Hsueh Lu Birsoy K Werner E Fayzikhodjaeva G . Dysregulation of a long noncoding RNA reduces leptin leading to a leptin-responsive form of obesity. Nat Med. (2019) 25:507–16. doi: 10.1038/s41591-019-0370-1, PMID: 30842678

[70] Münzberg H Heymsfield SB . New insights into the regulation of leptin gene expression. Cell Metab. (2019) 29:1013–4. doi: 10.1016/j.cmet.2019.04.005, PMID: 31067443 PMC7346278

[71] Ahn J Wu H Lee K . Integrative analysis revealing human adipose-specific genes and consolidating obesity loci. Sci Rep. (2019) 9:3087. doi: 10.1038/s41598-019-39582-8, PMID: 30816281 PMC6395763

[72] Wu Y-K Watson HJ Re ACD Finch JE Hardin SL Dumain AS . Peripheral biomarkers of anorexia nervosa: A meta-analysis. Nutrients. (2024) 16:2095. doi: 10.3390/nu16132095, PMID: 38999843 PMC11243150

[73] Haines MS . Endocrine complications of anorexia nervosa. J Eat Disord. (2023) 11:24. doi: 10.1186/s40337-023-00744-9, PMID: 36793059 PMC9933399

[74] Schorr M Miller KK . The endocrine manifestations of anorexia nervosa: mechanisms and management. Nat Rev Endocrinol. (2017) 13:174–86. doi: 10.1038/nrendo.2016.175 PMC5998335 27811940

[75] Germain N Galusca B Roux CWL Bossu C Ghatei MA Lang F . Constitutional thinness and lean anorexia nervosa display opposite concentrations of peptide YY, glucagon-like peptide 1, ghrelin, and leptin. Am J Clin Nutr. (2007) 85:967–71. doi: 10.1093/ajcn/85.4.967, PMID: 17413094

[76] Fanin A Miele L Bertolini E Giorgini A Pontiroli AE Benetti A . Liver alterations in anorexia nervosa are not caused by insulin resistance. Intern Emerg Med. (2020) 15:337–9. doi: 10.1007/s11739-019-02227-9, PMID: 31734856

[77] Kim Y Hildebrandt T Mayer LES . Differential glucose metabolism in weight restored women with anorexia nervosa. Psychoneuroendocrinology. (2019) 110:104404. doi: 10.1016/j.psyneuen.2019.104404, PMID: 31541915 PMC8666139

[78] Verebi C Lebrun N Petit JV Viltart O Duriez P Saint-Pierre B . Potential new expression biomarkers for anorexia nervosa. Am J Méd Genet Part B: Neuropsychiatr Genet. (2025) 198:e33018. doi: 10.1002/ajmg.b.33018 39660767

[79] Golden NH Walsh BT . Time to revisit the definition of atypical anorexia nervosa. Int J Eat Disord. (2024) 57:757–60. doi: 10.1002/eat.24174, PMID: 38390637

[80] Camacho-Barcia L Giel KE Jiménez-Murcia S Pitti JÁ Micali N Lucas I . Eating disorders and obesity: bridging clinical, neurobiological, and therapeutic perspectives. Trends Mol Med. (2024) 30:361–79. doi: 10.1016/j.molmed.2024.02.007, PMID: 38485648

[81] Watson HJ Yilmaz Z Thornton LM Hübel C Coleman JRI Gaspar HA . Genome-wide association study identifies eight risk loci and implicates metabo-psychiatric origins for anorexia nervosa. Nat Genet. (2019) 51:1207–14. doi: 10.1038/s41588-019-0439-2, PMID: 31308545 PMC6779477

[82] Scharner S Stengel A . Animal models for anorexia nervosa—A systematic review. Front Hum Neurosci. (2021) 14:596381. doi: 10.3389/fnhum.2020.596381, PMID: 33551774 PMC7854692

[83] Chamorro-García R Shoucri BM Willner S Käch H Janesick A Blumberg B . Effects of perinatal exposure to dibutyltin chloride on fat and glucose metabolism in mice, and molecular mechanisms, *in vitro*. Environ Health Persp. (2018) 126:057006. doi: 10.1289/ehp3030, PMID: 29787037 PMC6072003

[84] Lin M-Y Lo Y-C Hung J-H . Unveiling chromatin dynamics with virtual epigenome. Nat Commun. (2025) 16:3491. doi: 10.1038/s41467-025-58481-3, PMID: 40221401 PMC11993739

