# Supplementary material for: Preconception exposures of female mice to a panel of metabolic disruptors induce sexually dimorphic metabolic perturbations in their offspring
Source: Front Endocrinol (Lausanne). 2026 Apr 2;17:1787973. doi: 10.3389/fendo.2026.1787973 (PMC13082944; doi:10.3389/fendo.2026.1787973)
Supplement: Supplementary file 1 [file Supplementaryfile1.zip › Data Sheet 1_updated/Supplementary File Captions.pdf]

Supplementary Files

Supplementary Figure 1. Relative gene distribution within each isochore class for all autosomes and the *X* chromosome for genes included in each exposure group *versus* control contrast.
Supplementary Figure 2. Relative gene distribution within each isochore class for 10 GO-BP terms selected from the results in Figure 3B for genes included in each exposure group *versus* control contrast.
Supplementary Data 1. Data to draw F0 and F1 timelines using the R package *ggplot2*.
Supplementary Data 2. Dates for F0 and F1 timepoints.
Supplementary Data 3. Dates for F0 food consumption timepoints (entfood: entry food, exfood: exit food).
Supplementary Data 4. Mouse exposure groups, generations, sexes, and birthdates.
Supplementary Data 5. Mouse cage exposure group, generation, sex, and number of mice per cage (N).
Supplementary Data 6. F0 mouse body weights [g] per date.
Supplementary Data 7. F0 mouse fasting body weights [g] per date.
Supplementary Data 8. F0 mouse cage exit water [mL] per date.
Supplementary Data 9. F0 mouse cage entry food [g] per date.
Supplementary Data 10. F0 mouse cage exit food [g] per date.
Supplementary Data 11. F1 mouse litter size and sex composition.
Supplementary Data 12. F1 mouse body weights [g] per date.
Supplementary Data 13. F1 mouse fasting body weights [g] per date.
Supplementary Data 14. F1 mouse fasting plasma glucose [mg/dL] per date.
Supplementary Data 15. F1 mouse dissection date, weekly body weight (BW)[g], fasting body weight (fBW)[g], fasting plasma glucose (fglucose)[mg/dL], and weights of inguinal white adipose tissue (iWAT), gonadal white adipose tissue (gWAT), and liver [mg].
Supplementary Data 16. F1 mouse plasma levels of amylin, GIP, GLP-1, ghrelin, insulin, leptin, PYY, glucagon, PP, resistin, and C-peptide [pg/mL].
Supplementary Data 17. Mouse gene chromosome locations, isochore overlap, and RNA-seq read counts in female (F) and male (M), gonadal white adipose tissue (G), and liver (L) samples.
Supplementary Data 18. Isochore composition of the mouse genome determined using *isoSegmenter*.
Supplementary Data 19. Gene Ontology term name translator.
Supplementary Data 20. Gene content of isochores that include the gene encoding for Leptin (Lep), or members of the Nduf and Sema gene families.
Supplementary Data 21. Results of unmatched-measures Monte Carlo-Wilcoxon (uMCW) tests for mouse traits.
Supplementary Data 22. Results of matched-measures bivariate Monte Carlo-Wilcoxon (mbMCW) tests for mouse traits.
Supplementary Data 23. Results of differential gene expression analyses using unmatched-measures Monte Carlo-Wilcoxon (uMCW) tests.
Supplementary Data 24. Results of Gene Ontology-based Gene Set Enrichment Analyses using *fgsea* R package.
Supplementary Data 25. Results of biased-measures Monte Carlo-Wilcoxon tests for the whole transcriptome, isochore classes, and main nuclear chromosomes.
Supplementary Data 26. Isochore distribution of genes located in the same chromosome or associated with the same Gene Ontology term.

Supplementary Data 27. Results of bMCW tests for isochores where genes in Nduf and Sema gene families are located.
Supplementary Code. R code to perform analyses and prepare all figures and supplementary figures.
