# Supplementary material for: Preconception exposures of female mice to a panel of metabolic disruptors induce sexually dimorphic metabolic perturbations in their offspring
Source: Front Endocrinol (Lausanne). 2026 Apr 2;17:1787973. doi: 10.3389/fendo.2026.1787973 (PMC13082944; doi:10.3389/fendo.2026.1787973)
Supplement: Supplementary file 1 [file Supplementaryfile1.zip › Data Sheet 1_updated/Supplementary Code.pdf]

```r
# This file contains the R code necessary to replicate all analyses and figures presented in the publication Diaz-Castillo et al., Frontiers in Endocrinology, 2026 (https://www.frontiersin.org/journals/endocrinology/articles/10.3389/fendo.2026.1787973/abstract)

# Prior to executing this code, the user must download all Supplementary files associated with this publication into a directory of their choice.

# The code will subsequently create three subdirectories and generate a single RData file.
#   - The "temp" subdirectory will be utilized to store files necessary for advancing the analyses and will be deleted during the final step of the code.
#   - The "File confirmations" subdirectory contains two additional subdirectories: "Supplementary files" and "Figures" The "Supplementary files" directory houses copies of Supplementary files for this publication that contain analytical results, including Supplementary Data 21-27 and Supplementary Figures 1 and 2. The "Figures" directory houses copies of the main figures of the publication. These copies will enable the user to compare them with the corresponding files accompanying this publication.
#   - The RData file "2026_Diaz_Castillo_et_al.RData" will be periodically saved to store all main objects resulting from the code's progress. This will provide the user with greater control over inspecting the code's progress and even modifying it if desired.

# Executing this code will require several days or hours depending on the user setup.
# Commented lines in specific sections will inform the user of the steps that require the most time.

# 1. USER SETUP ----
# Please ensure that you download all supplementary files associated with this publication into a directory of your preference and set this directory as the working directory prior to executing the code. While the user has the option to select any directory, we strongly recommend creating a new directory to facilitate the organization of the starting material and the outcomes of code execution.

# Saving RData file
save.image(file = "2026_Diaz_Castillo_et_al.RData")

# 2. INSTALLING NEEDED PACKAGES ----
# This section will install, if necessary, all R packages required to execute this code.

if (!require("BiocManager", quietly = TRUE)) {install.packages("BiocManager"); library(BiocManager)}
if (!require("ComplexHeatmap", quietly = TRUE)) {BiocManager::install("ComplexHeatmap"); library (ComplexHeatmap)}
if (!require("data.table", quietly = TRUE)) {install.packages("data.table"); library (data.table)}
if (!require("devtools", quietly = TRUE)) {install.packages("devtools"); library (devtools)}
if (!require("factoextra", quietly = TRUE)) {install.packages("factoextra"); library (factoextra)}
if (!require("fgsea", quietly = TRUE)) {BiocManager::install("fgsea"); library (fgsea)}
if (!require("ggplot2", quietly = TRUE)) {install.packages("ggplot2"); library (ggplot2)}
if (!require("ggplotify", quietly = TRUE)) {install.packages("ggplotify"); library (ggplotify)}
if (!require("ggrepel", quietly = TRUE)) {install.packages("ggrepel"); library (ggrepel)}
if (!require("ggtext", quietly = TRUE)) {install.packages("ggtext"); library (ggtext)}
if (!require("MCWtests", quietly = TRUE)) {devtools::install_github("diazcastillo/MCWtests"); library (MCWtests)}
if (!require("msigdbr", quietly = TRUE)) {install.packages("msigdbr"); library(msigdbr)}
if (!require("patchwork", quietly = TRUE)) {install.packages("patchwork"); library (patchwork)}
if (!require("RColorBrewer", quietly = TRUE)) {install.packages("RColorBrewer"); library (RColorBrewer)}
if (!require("scales", quietly = TRUE)) {install.packages("scales"); library (scales)}
if (!require("smplot2", quietly = TRUE)) {install.packages("smplot2"); library (smplot2)}

# Saving RData file
save.image(file = "2026_Diaz_Castillo_et_al.RData")

# 3. PREPARATION ----
# This section will generate R objects including keys for preparing data for analysis, rendering analysis results, and preparing figures. Furthermore, it will write three subdirectories and a single RData file within the working directory to store the generated files and R objects. The RData file will be updated after the execution of each primary section
```

of the code.

```
## 3.1. Writing key tables ----
key_mouse <- fread("Supplementary Data 4.csv")

key_cage <- fread("Supplementary Data 5.csv")

key_timepoint <- fread("Supplementary Data 2.csv")[
  , `:=`(date, as.character(date))]

key_food_consumption <- fread("Supplementary Data 3.csv")

key_contrast <- data.table(condition_a = c("5TBT", "50TBT", "TWD", "IAS"),
                           condition_b = ("DMSO"))[
                             , `:=`(contrast, paste0(condition_a, "-", condition_b))]

key_contrast_mouse <- setnames(cbind(element = "mouse",
                                     rbindlist(lapply(split(copy(key_contrast), by =
"contrast"),
                                                      function(x){
                                                        i <- rbind(cbind(contrast = x[,
contrast],
                                                                         rbind(copy(key_mouse)
[exposure == x[, condition_a] & generation == "F0", ],
                                                                               copy(key_mouse)
[exposure == x[, condition_b] & generation == "F0", ])),
                                                                   cbind(contrast = x[,
contrast],
                                                                         rbind(copy(key_mouse)
[exposure == x[, condition_a] & generation == "F1" & sex == "Female", ],
                                                                               copy(key_mouse)
[exposure == x[, condition_b] & generation == "F1" & sex == "Female", ])),
                                                                   cbind(contrast = x[,
contrast],
                                                                         rbind(copy(key_mouse)
[exposure == x[, condition_a] & generation == "F1" & sex == "Male", ],
                                                                               copy(key_mouse)
[exposure == x[, condition_b] & generation == "F1" & sex == "Male", ])))
                                                      }))), "mouse_ID", "element_ID")

key_contrast_cage <- setnames(cbind(element = "cage",
                                    rbindlist(lapply(split(copy(key_contrast), by =
"contrast"),
                                                     function(x){
                                                       i <- rbind(cbind(contrast = x[,
contrast],
                                                                        rbind(copy(key_cage)
[exposure == x[, condition_a] & generation == "F0", ],
                                                                              copy(key_cage)
[exposure == x[, condition_b] & generation == "F0", ])),
                                                                  cbind(contrast = x[,
contrast],
                                                                        rbind(copy(key_cage)
[exposure == x[, condition_a] & generation == "F1" & sex == "Female", ],
                                                                              copy(key_cage)
[exposure == x[, condition_b] & generation == "F1" & sex == "Female", ])),
                                                                  cbind(contrast = x[,
contrast],
                                                                        rbind(copy(key_cage)
[exposure == x[, condition_a] & generation == "F1" & sex == "Male", ],
                                                                              copy(key_cage)
[exposure == x[, condition_b] & generation == "F1" & sex == "Male", ])))
                                                     }))), "cage_ID", "element_ID")

key_contrast_transcriptomics <- data.table(contrast_ID = c(1:16),
                                           condition_a = c("G_F_5TBT", "G_F_50TBT", "G_F_IAS",
"G_F_TWD",
                                                           "G_M_5TBT", "G_M_50TBT", "G_M_IAS",
"G_M_TWD",
                                                           "L_F_5TBT", "L_F_50TBT", "L_F_IAS",
```

```
"L_F_TWD",
                                                            "L_M_5TBT", "L_M_50TBT", "L_M_IAS",
"L_M_TWD"),
                                               condition_b = c(rep("G_F_DMSO", times = 4),
                                                               rep("G_M_DMSO", times = 4),
                                                               rep("L_F_DMSO", times = 4),
                                                               rep("L_M_DMSO", times = 4)))[
                                                                 , `:=`(contrast,
paste0(condition_a, "-", condition_b))]

key_gene_ontology <- as.data.table(msigdbr(species = "Mus musculus", db_species = "MM",
collection = "M5", subcollection = "GO:BP"))[
  , `:=`(gs_name, gsub("^GOBP_", "", gs_name))]

key_gene_ontology_translator <- fread("Supplementary Data 19.csv")

## 3.2. Creating subdirectories and RData files in the working directory ----
dir.create("temp")
dir.create("File confirmations")
dir.create("File confirmations/Supplementary files")
dir.create("File confirmations/Figures")

# Saving RData file
save.image(file = "2026_Diaz_Castillo_et_al.RData")

# 4. ANALYSIS OF NON-TRANSCRIPTOMIC DATA ----
# This section will conduct all analyses for non-transcriptomic data included in this
publication.
## 4.1. Monte Carlo-Wilcoxon tests of non-transcriptomic data ----
### 4.1.a. Preparing data ----
#### Data table for unmatched-measures MCW (uMCW) tests with F0 body weights ----
uMCW_F0_BW <- setnames(merge(na.omit(cbind(trait = "BW",
                                           melt(merge(copy(key_contrast_mouse),
                                                      fread("Supplementary Data 6.csv"),
                                                      by.x = "element_ID", by.y = "mouse_ID",
sort = FALSE),
                                                measure.vars = patterns(".-"),
                                                variable.name = "date",
                                                value.name = "BW")))[
                                                  , `:=`(value = BW / (as.IDate(date) -
birthdate))],
                             copy(key_timepoint)[, `:=`(generation, NULL)], by = "date", all.x
= TRUE, sort = FALSE),
                       c("exposure", "timepoint"), c("condition", "variable"))[
                         , .(trait, element, contrast, generation, sex, variable, element_ID,
condition, value)]

#### Data table for matched-measures bivariate MCW (mbMCW) tests with F0 fasting body weights -
---
mbMCW_F0_fBW <- setnames(merge(na.omit(cbind(trait = "fBW", matched_condition_a = "BW",
matched_condition_b = "fBW",
                                             merge(melt(merge(copy(key_contrast_mouse),
                                                              fread("Supplementary Data
6.csv"),
                                                              by.x = "element_ID", by.y =
"mouse_ID", sort = FALSE),
                                                        measure.vars = c(9:12),
                                                        variable.name = "date",
                                                        value.name = "BW"),
                                                   melt(merge(copy(key_contrast_mouse),
                                                              fread("Supplementary Data
7.csv"),
                                                              by.x = "element_ID", by.y =
"mouse_ID", sort = FALSE),
                                                        measure.vars = patterns("-"),
                                                        variable.name = "date",
                                                        value.name = "fBW"),
                                                   sort = FALSE))),
                               copy(key_timepoint)[, `:=`(generation, NULL)], by = "date",
```

```
all.x = TRUE, sort = FALSE)[
                                      , `:=`(value_a = BW / (as.IDate(date) - birthdate),
                                             value_b = fBW / (as.IDate(date) - birthdate))],
                              c("exposure", "timepoint"), c("unmatched_condition", "variable"))[
                                , .(trait, element, contrast, generation, sex, element_ID,
matched_condition_a, matched_condition_b, unmatched_condition, variable, value_a, value_b)]

#### Data table for mbMCW tests with F0 water consumption ----
mbMCW_F0_water <- setnames(merge(cbind(trait = "water", matched_condition_a = "entwater",
matched_condition_b = "exwater", value_a = 200,
                                       na.omit(melt(merge(copy(key_contrast_cage)[generation ==
"F0", ],
                                                          fread("Supplementary Data 8.csv")[
                                                            , `:=`(element_ID,
as.character(cage_ID))],
                                                          by = "element_ID", all.x = TRUE, sort
= FALSE),
                                                    measure.vars = patterns("-"),
                                                    variable.name = "date",
                                                    value.name = "exwater"))),
                                 copy(key_timepoint)[, `:=`(generation, NULL)], by = "date",
all.x = TRUE, sort = FALSE)[
                                   , `:=`(value_b = (200 - exwater))],
                           c("exposure", "timepoint"), c("unmatched_condition", "variable"))[
                             , .(trait, element, contrast, generation, sex, element_ID,
matched_condition_a, matched_condition_b, unmatched_condition, variable, value_a, value_b)]

#### Data table for mbMCW tests with F0 food consumption by weight ----
mbMCW_F0_food_g <- setnames(cbind(trait = "food_g", matched_condition_a = "entfood",
matched_condition_b = "exfood",
                                  na.omit(merge(merge(melt(merge(copy(key_contrast_cage)
[generation == "F0", ],
                                                                 fread("Supplementary Data
9.csv")[
                                                                   , `:=`(element_ID,
as.character(cage_ID))],
                                                                 by = "element_ID", all.x =
TRUE, sort = FALSE),
                                                           measure.vars = patterns("-"),
                                                           variable.name = "entfood_date",
                                                           value.name = "entfood")[
                                                             , `:=`(entfood_date =
as.IDate(entfood_date))],
                                                      copy(key_food_consumption)[,
`:=`(generation, NULL)], by = "entfood_date", all.x = TRUE, sort = FALSE),
                                                merge(melt(merge(copy(key_contrast_cage)
[generation == "F0", ],
                                                                 fread("Supplementary Data
10.csv")[
                                                                   , `:=`(element_ID,
as.character(cage_ID))],
                                                                 by = "element_ID", all.x =
TRUE, sort = FALSE),
                                                           measure.vars = patterns("-"),
                                                           variable.name = "exfood_date",
                                                           value.name = "exfood")[
                                                             , `:=`(exfood_date =
as.IDate(exfood_date))],
                                                      copy(key_food_consumption)[,
`:=`(generation, NULL)], by = "exfood_date", all.x = TRUE, sort = FALSE),
                                                all.x = TRUE, sort = FALSE)))[
                                                  , `:=`(value_a = entfood,
                                                         value_b = (entfood - exfood))],
                            c("exposure", "timepoint"), c("unmatched_condition", "variable"))[
                              , .(trait, element, contrast, generation, sex, element_ID,
matched_condition_a, matched_condition_b, unmatched_condition, variable, value_a, value_b)]

#### Data table for mbMCW tests with F0 food consumption by caloric content ----
mbMCW_F0_food_kcal <- copy(mbMCW_F0_food_g)[, `:=`(trait = "food_kcal",
```

```
                                                          value_a = fifelse(unmatched_condition ==
"TWD", 4.4 * value_a, 3.8 * value_a),
                                                          value_b = fifelse(unmatched_condition ==
"TWD", 4.4 * value_b, 3.8 * value_b))]

#### Data table for uMCW tests with F1 litter size and composition ----
uMCW_F1_litter <- setnames(cbind(variable = "mating",
                                 na.omit(melt(merge(copy(key_contrast_mouse),
                                                   fread("Supplementary Data 11.csv"),
                                                   by.x = "element_ID", by.y = "mouse_ID",
sort = FALSE)[
                                                     , `:=`(female_fraction = female_N /
litter_size)][
                                                       , `:=`(litter_size,
as.numeric(litter_size))],
                                              measure.vars = c("litter_size",
"female_fraction"),
                                              variable.name = "trait",
                                              value.name = "value"))),
                           "exposure", "condition")[
                             , .(trait, element, contrast, generation, sex, variable,
element_ID, condition, value)]

#### Data table for uMCW tests with F1 body weights ----
uMCW_F1_BW <- rbind(setnames(merge(na.omit(cbind(trait = "BW",
                                                 melt(merge(copy(key_contrast_mouse),
                                                            fread("Supplementary Data 12.csv"),
                                                            by.x = "element_ID", by.y =
"mouse_ID", sort = FALSE),
                                                      measure.vars = patterns(".-"),
                                                      variable.name = "date",
                                                      value.name = "BW")))[
                                                        , `:=`(value, BW / (as.IDate(date) -
birthdate))],
                                   copy(key_timepoint)[, `:=`(generation, NULL)], by = "date",
all.x = TRUE, sort = FALSE),
                             c("exposure", "timepoint"), c("condition", "variable"))[
                               , .(trait, element, contrast, generation, sex, variable,
element_ID, condition, value)],
                    setnames(cbind(trait = "BW", variable = "Euth.",
                                   merge(copy(key_contrast_mouse),
                                         fread("Supplementary Data 15.csv"),
                                         by.x = "element_ID", by.y = "mouse_ID", sort = FALSE))
[
                                           , `:=`(value, BW / (dissection_date - birthdate))],
                             "exposure", "condition")[
                               , .(trait, element, contrast, generation, sex, variable,
element_ID, condition, value)])

#### Data table for mbMCW tests with F1 fasting body weights ----
mbMCW_F1_fBW <- rbind(setnames(cbind(trait = "fBW", matched_condition_a = "BW",
matched_condition_b = "fBW",
                                     merge(merge(na.omit(melt(merge(copy(key_contrast_mouse),
                                                                    fread("Supplementary Data
12.csv"),
                                                                    by.x = "element_ID", by.y =
"mouse_ID", sort = FALSE),
                                                              measure.vars = patterns("-"),
                                                              variable.name = "date_BW",
                                                              value.name = "BW")),
                                                 copy(key_timepoint)[, `:=`(generation, NULL)],
by.x = "date_BW", by.y = "date", all.x = TRUE, sort = FALSE)[
                                                   timepoint == "T7", ],
                                           merge(na.omit(melt(merge(copy(key_contrast_mouse),
                                                                    fread("Supplementary Data
13.csv"),
                                                                    by.x = "element_ID", by.y =
"mouse_ID", sort = FALSE),
                                                              measure.vars = patterns("-"),
```

```
                                                                  variable.name = "date_fBW",
                                                                  value.name = "fBW")),
                                                     copy(key_timepoint)[, `:=`(generation, NULL)],
by.x = "date_fBW", by.y = "date", all.x = TRUE, sort = FALSE)[
                                                       timepoint == "T7", ],
                                               sort = FALSE)),
                                   c("exposure", "timepoint"), c("unmatched_condition",
"variable"))[
                                     , `:=`(value_a = (BW / (as.IDate(date_BW) - birthdate)),
                                            value_b = (fBW / (as.IDate(date_fBW) - birthdate)))][
                                              , .(trait, element, contrast, generation, sex,
element_ID, matched_condition_a, matched_condition_b, unmatched_condition, variable, value_a,
value_b)],
                         setnames(cbind(trait = "fBW", matched_condition_a = "BW",
matched_condition_b = "fBW", variable = "Euth.",
                                         na.omit(merge(copy(key_contrast_mouse),
                                                       fread("Supplementary Data 15.csv"),
                                                       by.x = "element_ID", by.y = "mouse_ID", sort
= FALSE)))[
                                                         , `:=`(value_a = (BW / ((dissection_date
-1) - birthdate)),
                                                                value_b = (fBW / (dissection_date -
birthdate)))],
                                   "exposure", "unmatched_condition")[
                                     , .(trait, element, contrast, generation, sex, element_ID,
matched_condition_a, matched_condition_b, unmatched_condition, variable, value_a, value_b)])

#### Data table for uMCW tests with F1 fasting glucose at T7 timepoint ----
uMCW_F1_fglucose <- setnames(cbind(trait = "fglucose", variable = "T7",
                                       merge(na.omit(melt(merge(copy(key_contrast_mouse),
                                                                fread("Supplementary Data 14.csv"),
                                                                by.x = "element_ID", by.y =
"mouse_ID", sort = FALSE),
                                                          measure.vars = patterns(".-"),
                                                          variable.name = "date",
                                                          value.name = "fglucose")),
                                             na.omit(melt(fread("Supplementary Data 13.csv"),
                                                          measure.vars = patterns(".-"),
                                                          variable.name = "date",
                                                          value.name = "fBW")),
                                             by.x = c("element_ID", "date"), by.y = c("mouse_ID",
"date"), sort = FALSE))[
                                               , `:=`(value = (fglucose / fBW) / (as.IDate(date) -
birthdate))],
                                 "exposure", "condition")[
                                   , .(trait, element, contrast, generation, sex, variable,
element_ID, condition, value)]

#### Data table for uMCW tests with F1 fasting glucose, and inguinal adipose tissue (iWAT),
gonadal white adipose tissue (gWAT) and liver weight at euthanasia ----
uMCW_F1_dissection <- setnames(cbind(variable = "Euth.",
                                         na.omit(melt(merge(copy(key_contrast_mouse),
                                                            fread("Supplementary Data 15.csv"),
                                                            by.x = "element_ID", by.y = "mouse_ID",
sort = FALSE)[
                                                              , `:=`(fglucose_BW_age = (fglucose /
fBW) / (dissection_date - birthdate),
                                                                     iWAT_BW_age = (iWAT_weight /
fBW) / (dissection_date - birthdate),
                                                                     gWAT_BW_age = (gWAT_weight /
fBW) / (dissection_date - birthdate),
                                                                     liver_BW_age = (liver_weight /
fBW) / (dissection_date - birthdate))],
                                                      measure.vars = patterns("_BW"),
                                                      variable.name = "trait",
                                                      value.name = "value")))[
                                                        , `:=`(trait, gsub("_BW_age", "", trait))],
                                   "exposure", "condition")[
                                     , .(trait, element, contrast, generation, sex, variable,
```

```
element_ID, condition, value)]

#### Data table for uMCW tests with F1 plasma metabolite levels ----
uMCW_F1_plasma <- setnames(cbind(variable = "Euth.",
                                 na.omit(melt(merge(merge(copy(key_contrast_mouse),
                                                          fread("Supplementary Data 15.csv"),
                                                          by.x = "element_ID", by.y =
"mouse_ID", sort = FALSE),
                                                    fread("Supplementary Data 16.csv"),
                                                    by.x = "element_ID", by.y = "mouse_ID",
sort = FALSE),
                                              measure.vars = c(15:25),
                                              variable.name = "trait",
                                              value.name = "absolute_value"))[
                                                , `:=`(value = (absolute_value / fBW) /
(dissection_date - birthdate))]),
                           "exposure", "condition")[
                             , .(trait, element, contrast, generation, sex, variable,
element_ID, condition, value)]

### 4.1.b. Writing data tables for uMCW and mbMCW tests to the “temp” subdirectory ----
fwrite(rbindlist(mget(ls(pattern = "uMCW_")))[, `:=`(element_ID, NULL)], file =
"./temp/mouse_uMCWtest_data.csv", row.names = FALSE)
fwrite(rbindlist(mget(ls(pattern = "mbMCW_")))[, `:=`(element_ID, NULL)], file =
"./temp/mouse_mbMCWtest_data.csv", row.names = FALSE)

### 4.1.c. Executing uMCW and mbMCW tests ----
# This step will take a considerable amount of time.
uMCWtest("./temp/mouse_uMCWtest_data.csv", 10000)
mbMCWtest("./temp/mouse_mbMCWtest_data.csv", 10000)

### 4.1.d. Retrieving MCW test results ----
uMCW_mouse_results <- fread("./temp/mouse_uMCWtest_results.csv")[
  BI_type == "uMCW_BI" & contrast == condition_contrast, ][
    , `:=`(significance = fcase(pupper < 0.05, "1",
                                plower < 0.05, "-1",
                                pmin(pupper, plower) >= 0.05, "NO"))]

mbMCW_mouse_results <- fread("./temp/mouse_mbMCWtest_results.csv")[
  contrast == unmatched_condition_contrast & matched_condition_contrast ==
paste0(matched_condition_b, "-", matched_condition_a), ][
    , `:=`(significance = fcase(pupper < 0.05, "1",
                                plower < 0.05, "-1",
                                pmin(pupper, plower) >= 0.05, "NO"))]

### 4.1.e. Writing copies of Supplementary Data files 21 and 22 with uMCW and mbMCW test
results to the “File confirmations/Supplementary files” subdirectory ----
fwrite(setnames(setorder(copy(uMCW_mouse_results)[
  , .(trait, generation, sex, variable, condition_a, condition_b, condition_contrast, N, n_a,
n_b, test_type, observed_BI, expected_by_chance_BI_N, pupper, plower)][
    , `:=`(trait = factor(trait, levels = c("BW", "litter_size", "female_fraction", "fglucose",
"iWAT", "gWAT", "liver", "Amylin", "GIP", "Ghrelin", "GLP-1", "Insulin", "Leptin", "PYY",
"Glucagon", "PP", "Resistin", "C-Peptide")),
           variable = factor(variable, levels = c("Entry", "T0", "T1", "T2", "T3", "T3.5",
"T4", "T5", "T6", "T7", "T8", "T9", "T10", "Euth.", "mating")),
           condition_a = factor(condition_a, levels = c("5TBT", "50TBT", "IAS", "TWD")))],
  generation, sex, trait, condition_a, variable)[
    , `:=`(trait = fcase(trait == "BW", "Bodyweight by age [g/days old]",
                         trait == "fglucose", "Fasting glucose by bodyweight and age
[(mg/dL)/g/days old]",
                         trait == "iWAT", "iWAT weight by bodyweight and age [mg/g/days old]",
                         trait == "gWAT", "gWAT weight by bodyweight and age [mg/g/days old]",
                         trait == "liver", "Liver weight by bodyweight and age [mg/g/days
old]",
                         trait == "litter_size", "Litter size [N]",
                         trait == "female_fraction", "Female fraction [n/N]",
                         trait == "Amylin", "Amylin plasma level by bodyweight and age
[(pg/mL)/g/days old]",
                         trait == "GIP", "GIP plasma level by bodyweight and age
```

```
[(pg/mL)/g/days old]",
                         trait == "GLP-1", "GLP-1 plasma level by bodyweight and age
[(pg/mL)/g/days old]",
                         trait == "Ghrelin", "Ghrelin plasma level by bodyweight and age
[(pg/mL)/g/days old]",
                         trait == "Insulin", "Insulin plasma level by bodyweight and age
[(pg/mL)/g/days old]",
                         trait == "Leptin", "Leptin plasma level by bodyweight and age
[(pg/mL)/g/days old]",
                         trait == "PYY", "PYY plasma level by bodyweight and age
[(pg/mL)/g/days old]",
                         trait == "Glucagon", "Glucagon plasma level by bodyweight and age
[(pg/mL)/g/days old]",
                         trait == "PP", "PP plasma level by bodyweight and age [(pg/mL)/g/days
old]",
                         trait == "Resistin", "Resistin plasma level by bodyweight and age
[(pg/mL)/g/days old]",
                         trait == "C-Peptide", "C-Peptide plasma level by bodyweight and age
[(pg/mL)/g/days old]"))][
                           , `:=`(variable = gsub("Euth.", "Euthanasia", variable))][
                             , `:=`(variable = gsub("mating", "F1 litter", variable))][
                               , `:=`(test_type = ifelse(test_type == "exact", "Exact",
"Approximated"))],
  c("Trait", "Generation", "Sex", "Timepoint", "Condition a", "Condition b", "Condition
contrast", "N", "N condition a", "N condition b", "uMCW test type", "Observed uMCW_BI", "N
expected-by-chance uMCW_BIs", "Pupper value", "Plower value")),
  file = "./File confirmations/Supplementary files/Supplementary Data 21.csv", row.names =
FALSE)

fwrite(setnames(setorder(copy(mbMCW_mouse_results)[
  , .(trait, generation, sex, variable, matched_condition_a, matched_condition_b,
unmatched_condition_x, unmatched_condition_y, matched_condition_contrast,
unmatched_condition_contrast, N, N_x, N_y, test_type, observed_BI, expected_by_chance_BI_N,
pupper, plower)][
    , `:=`(trait = factor(trait, levels = c("fBW", "water", "food_g", "food_kcal")),
           variable = factor(variable, levels = c("T0", "T0.5", "T1", "T1.5", "T2", "T2.5",
"T3", "T3.5", "T7", "Euth.")),
           unmatched_condition_x = factor(unmatched_condition_x, levels = c("5TBT", "50TBT",
"IAS", "TWD")))],
  generation, sex, trait, unmatched_condition_x, variable)[
    , `:=`(trait = fcase(trait == "fBW", "Fasting bodyweight by age [g/days old]",
                         trait == "water", "Semiweekly water consumption [mL]",
                         trait == "food_g", "Weekly food consumption [g]",
                         trait == "food_kcal", "Weekly food consumption [kcal]"),
           matched_condition_contrast = fcase(matched_condition_contrast == "fBW-BW", "Fasting
bodyweight-Prefasting bodyweight",
                                              matched_condition_contrast == "exwater-entwater",
"Exit water-Entry water",
                                              matched_condition_contrast == "exfood-entfood",
"Exit food-Entry food"),
           matched_condition_a = fcase(matched_condition_a == "BW", "Prefasting bodyweight",
                                       matched_condition_a == "entwater", "Entry water",
                                       matched_condition_a == "entfood", "Entry food"),
           matched_condition_b = fcase(matched_condition_b == "fBW", "Fasting bodyweight",
                                       matched_condition_b == "exwater", "Exit water",
                                       matched_condition_b == "exfood", "Exit food"))][
                                         , `:=`(variable = gsub("Euth.", "Euthanasia",
variable))][
                                           , `:=`(test_type = ifelse(test_type == "exact",
"Exact", "Approximated"))],
  c("Trait", "Generation", "Sex", "Timepoint", "Matched condition a", "Matched condition b",
"Unmatched condition x", "Unmatched condition y", "Matched condition contrast", "Unmatched
condition contrast", "N pairs", "N pairs unmatched condition x", "N pairs unmatched condition
y", "mbMCW test type", "Observed mbMCW_BI", "N expected-by-chance mbMCW_BIs", "Pupper value",
"Plower value")),
  file = "./File confirmations/Supplementary files/Supplementary Data 22.csv", row.names =
FALSE)

### 4.1.f. Drawing figure panels with results of uMCW and mbMCW tests ----
```

```
#### Figure panel for timepoint dynamics of F0 body weights ----
fig_panel_F0_BW_raw <- ggplot(unique(copy(uMCW_F0_BW)[
  , .(condition, variable, element_ID, value)])
  , aes(x = variable,
        y = value, group = interaction(condition, element_ID),
        colour = condition)) +
  geom_line(show.legend = FALSE) +
  facet_grid(~ factor(condition,
                      levels = c("DMSO", "5TBT", "50TBT", "IAS", "TWD"),
                      labels = c("DMSO (N=32)", "5TBT (N=32)", "50TBT (N=32)", "IAS (N=32)",
"TWD (N=32)"))) +
  scale_color_manual(values = c("red", "orange", "blue", "purple", "yellowgreen")) +
  labs(tag = "A",
       title = "Temporal bodyweight dynamics for each individual female mice",
       x = "Timepoint",
       y = "Bodyweight by age [g/days old]") +
  theme(panel.background = element_blank(),
        panel.border = element_rect(color = "black", fill = NA, linewidth = 1),
        panel.grid = element_line(color = "gray90"),
        plot.title = element_text(hjust = 0.5, size = 20),
        axis.ticks = element_blank(),
        axis.text = element_text(size = 15, color = "black"),
        axis.title.y = element_text(size = 15),
        strip.text = element_text(size = 15),
        strip.background = element_blank())

#### Figure panel for F0 body weight uMCW test results ----
fig_panel_F0_BW <- ggplot(copy(uMCW_mouse_results)[trait == "BW" & generation == "F0", ],
                          aes(x = factor(variable,
                                         levels = c("Entry", "T0", "T1", "T2", "T3", "T3.5")),
                              y = factor(condition_contrast,
                                         levels = c("TWD-DMSO", "IAS-DMSO", "50TBT-DMSO",
"5TBT-DMSO"),
                                         labels = c("TWD (N=32)\nvs\nDMSO (N=32)",
                                                    "IAS (N=32)\nvs\nDMSO (N=32)",
                                                    "50TBT (N=32)\nvs\nDMSO (N=32)",
                                                    "5TBT (N=32)\nvs\nDMSO (N=32)")))) +
  geom_tile(aes(fill = observed_BI), colour = "black", size = 0.25) +
  scale_fill_gradientn(colours = c("blue", "white", "red"),
                       values = rescale(c(-1, 0, 1)),
                       limits = c(-1, 1)) +
  geom_point(aes(shape = factor(significance)), size = 5, na.rm = TRUE) +
  scale_shape_manual(labels = c("Plower < 0.05", "Pupper < 0.05"),
                     values = c("1" = "\u25B2", "-1" = "\u25BC"),
                     limits = c(-1, 1)) +
  labs(tag = "A",
       title = "Bodyweight",
       x = "Timepoint",
       y = NULL,
       fill = "Bias index",
       shape = "Significance") +
  guides(fill = guide_colorbar(order = 1), shape = guide_legend(override.aes = list(size = 5),
order = 2)) +
  coord_fixed(ratio = 1) +
  theme(panel.background = element_blank(),
        panel.border = element_rect(color = "black", fill = NA, linewidth = 0),
        plot.title = element_text(hjust = 0.5),
        axis.ticks = element_blank(),
        axis.text = element_text(size = 15, color = "black"),
        axis.text.y = element_text(size = 12, hjust = 0.5),
        legend.key = element_blank(),
        strip.background = element_blank(),
        text = element_text(family = "sans", size = 10, color = "black"))

#### Figure panel for F0 fasting body weight mbMCW test results ----
fig_panel_F0_fBW <- ggplot(copy(mbMCW_mouse_results)[trait == "fBW" & generation == "F0", ],
                           aes(x = factor(variable,
                                          levels = c("Entry", "T0", "T1", "T2", "T3", "T3.5")),
                               y = factor(unmatched_condition_contrast,
```

```
                                          levels = c("TWD-DMSO", "IAS-DMSO", "50TBT-DMSO",
"5TBT-DMSO"),
                                          labels = c("TWD (N=32)\nvs\nDMSO (N=32)",
                                                     "IAS (N=32)\nvs\nDMSO (N=32)",
                                                     "50TBT (N=32)\nvs\nDMSO (N=32)",
                                                     "5TBT (N=32)\nvs\nDMSO (N=32)")))) +
  geom_tile(aes(fill = observed_BI), colour = "black", size = 0.25) +
  scale_fill_gradientn(colours = c("blue", "white", "red"),
                       values = rescale(c(-1, 0, 1)),
                       limits = c(-1, 1)) +
  geom_point(aes(shape = factor(significance)), size = 5, na.rm = TRUE) +
  scale_shape_manual(labels = c("Plower < 0.05", "Pupper < 0.05"),
                     values = c("1" = "\u25B2", "-1" = "\u25BC"),
                     limits = c(-1, 1)) +
  labs(tag = "A",
       title = "Fasting bodyweight",
       x = "Timepoint",
       y = NULL,
       fill = "Bias index",
       shape = "Significance") +
  guides(fill = guide_colorbar(order = 1), shape = guide_legend(override.aes = list(size = 5),
order = 2)) +
  coord_fixed(ratio = 1) +
  theme(panel.background = element_blank(),
        panel.border = element_rect(color = "black", fill = NA, linewidth = 0),
        plot.title = element_text(hjust = 0.5),
        axis.ticks = element_blank(),
        axis.text = element_text(size = 15, color = "black"),
        axis.text.y = element_text(size = 12, hjust = 0.5),
        legend.key = element_blank(),
        strip.background = element_blank(),
        text = element_text(family = "sans", size = 10, color = "black"))

#### Figure panel for F0 water consumption mbMCW test results ----
fig_panel_F0_water <- ggplot(copy(mbMCW_mouse_results)[trait == "water" & generation == "F0",
],
                              aes(x = factor(variable,
                                             levels = c("T0.5", "T1", "T1.5", "T2", "T2.5",
"T3", "T3.5")),
                                  y = factor(unmatched_condition_contrast,
                                             levels = c("TWD-DMSO", "IAS-DMSO", "50TBT-DMSO",
"5TBT-DMSO"),
                                             labels = c("TWD (N=8)\nvs\nDMSO (N=8)",
                                                        "IAS (N=8)\nvs\nDMSO (N=8)",
                                                        "50TBT (N=8)\nvs\nDMSO (N=8)",
                                                        "5TBT (N=8)\nvs\nDMSO (N=8)")))) +
  geom_tile(aes(fill = observed_BI), colour = "black", size = 0.25) +
  scale_fill_gradientn(colours = c("blue", "white", "red"),
                       values = rescale(c(-1, 0, 1)),
                       limits = c(-1, 1)) +
  geom_point(aes(shape = factor(significance)), size = 5, na.rm = TRUE) +
  scale_shape_manual(labels = c("Plower < 0.05", "Pupper < 0.05"),
                     values = c("1" = "\u25B2", "-1" = "\u25BC"),
                     limits = c(-1, 1)) +
  labs(tag = "A",
       title = "Semiweekly water consumption",
       x = "Timepoint",
       y = NULL,
       fill = "Bias index",
       shape = "Significance") +
  guides(fill = guide_colorbar(order = 1), shape = guide_legend(override.aes = list(size = 5),
order = 2)) +
  coord_fixed(ratio = 1) +
  theme(panel.background = element_blank(),
        panel.border = element_rect(color = "black", fill = NA, linewidth = 0),
        plot.title = element_text(hjust = 0.5),
        axis.ticks = element_blank(),
        axis.text = element_text(size = 15, color = "black"),
        axis.text.y = element_text(size = 12, hjust = 0.5),
```

```
        legend.key = element_blank(),
        strip.background = element_blank(),
        text = element_text(family = "sans", size = 10, color = "black"))

#### Figure panel for F0 food weight consumption mbMCW test results ----
fig_panel_F0_food_g <- ggplot(copy(mbMCW_mouse_results)[trait == "food_g" & generation == "F0",
],
                              aes(x = factor(variable,
                                             levels = c("T1", "T2", "T3", "T3.5")),
                                  y = factor(unmatched_condition_contrast,
                                             levels = c("TWD-DMSO", "IAS-DMSO", "50TBT-DMSO",
"5TBT-DMSO"),
                                             labels = c("TWD (N=8)\nvs\nDMSO (N=8)",
                                                        "IAS (N=8)\nvs\nDMSO (N=8)",
                                                        "50TBT (N=8)\nvs\nDMSO (N=8)",
                                                        "5TBT (N=8)\nvs\nDMSO (N=8)")))) +
  geom_tile(aes(fill = observed_BI), colour = "black", size = 0.25) +
  scale_fill_gradientn(colours = c("blue", "white", "red"),
                       values = rescale(c(-1, 0, 1)),
                       limits = c(-1, 1)) +
  geom_point(aes(shape = factor(significance)), size = 5, na.rm = TRUE) +
  scale_shape_manual(labels = c("Plower < 0.05", "Pupper < 0.05"),
                     values = c("1" = "\u25B2", "-1" = "\u25BC"),
                     limits = c(-1, 1)) +
  labs(tag = "A",
       title = "Weekly food consumption [g]",
       x = "Timepoint",
       y = NULL,
       fill = "Bias index",
       shape = "Significance") +
  guides(fill = guide_colorbar(order = 1), shape = guide_legend(override.aes = list(size = 5),
order = 2)) +
  coord_fixed(ratio = 1) +
  theme(panel.background = element_blank(),
        panel.border = element_rect(color = "black", fill = NA, linewidth = 0),
        plot.title = element_text(hjust = 1),
        axis.ticks = element_blank(),
        axis.text = element_text(size = 15, color = "black"),
        axis.text.y = element_text(size = 12, hjust = 0.5),
        legend.key = element_blank(),
        strip.background = element_blank(),
        text = element_text(family = "sans", size = 10, color = "black"))

#### Figure panel for F0 food caloric content consumption mbMCW test results ----
fig_panel_F0_food_kcal <- ggplot(copy(mbMCW_mouse_results)[trait == "food_kcal" & generation ==
"F0", ],
                                 aes(x = factor(variable,
                                                levels = c("T1", "T2", "T3", "T3.5")),
                                     y = factor(unmatched_condition_contrast,
                                                levels = c("TWD-DMSO", "IAS-DMSO", "50TBT-
DMSO", "5TBT-DMSO"),
                                                labels = c("TWD (N=8)\nvs\nDMSO (N=8)",
                                                           "IAS (N=8)\nvs\nDMSO (N=8)",
                                                           "50TBT (N=8)\nvs\nDMSO (N=8)",
                                                           "5TBT (N=8)\nvs\nDMSO (N=8)")))) +
  geom_tile(aes(fill = observed_BI), colour = "black", size = 0.25) +
  scale_fill_gradientn(colours = c("blue", "white", "red"),
                       values = rescale(c(-1, 0, 1)),
                       limits = c(-1, 1)) +
  geom_point(aes(shape = factor(significance)), size = 5, na.rm = TRUE) +
  scale_shape_manual(labels = c("Plower < 0.05", "Pupper < 0.05"),
                     values = c("1" = "\u25B2", "-1" = "\u25BC"),
                     limits = c(-1, 1)) +
  labs(tag = "A",
       title = "Weekly food consumption [kcal]",
       x = "Timepoint",
       y = NULL,
       fill = "Bias index",
       shape = "Significance") +
```

```
  guides(fill = guide_colorbar(order = 1), shape = guide_legend(override.aes = list(size = 5),
order = 2)) +
  coord_fixed(ratio = 1) +
  theme(panel.background = element_blank(),
        panel.border = element_rect(color = "black", fill = NA, linewidth = 0),
        plot.title = element_text(hjust = 0.8),
        axis.ticks = element_blank(),
        axis.text = element_text(size = 15, color = "black"),
        axis.text.y = element_text(size = 12, hjust = 0.5),
        legend.key = element_blank(),
        strip.background = element_blank(),
        text = element_text(family = "sans", size = 10, color = "black"))

#### Figure panel for F1 litter size and composition uMCW test results ----
fig_panel_F1_litter <- ggplot(copy(uMCW_mouse_results)[(trait == "litter_size" | trait ==
"female_fraction"), ],
                             aes(x = factor(trait,
                                            levels = c("litter_size", "female_fraction"),
labels = c("Litter\nsize", "Female\nfraction")),
                                 y = factor(condition_contrast,
                                            levels = c("TWD-DMSO", "IAS-DMSO", "50TBT-DMSO",
"5TBT-DMSO"),
                                            labels = c("TWD (N=5)\nvs\nDMSO (N=8)",
                                                       "IAS (N=6)\nvs\nDMSO (N=8)",
                                                       "50TBT (N=6)\nvs\nDMSO (N=8)",
                                                       "5TBT (N=13)\nvs\nDMSO (N=8)")))) +
  geom_tile(aes(fill = observed_BI), colour = "black", size = 0.25) +
  scale_fill_gradientn(colours = c("blue", "white", "red"),
                       values = rescale(c(-1, 0, 1)),
                       limits = c(-1, 1)) +
  geom_point(aes(shape = factor(significance)), size = 5, na.rm = TRUE) +
  scale_shape_manual(labels = c("Plower < 0.05", "Pupper < 0.05"),
                     values = c("1" = "\u25B2", "-1" = "\u25BC"),
                     limits = c(-1, 1)) +
  labs(tag = "A",
       title = "Litter composition",
       x = NULL,
       y = NULL,
       fill = "Bias index",
       shape = "Significance") +
  guides(fill = guide_colorbar(order = 1), shape = guide_legend(override.aes = list(size = 5),
order = 2)) +
  coord_fixed(ratio = 1) +
  theme(panel.background = element_blank(),
        panel.border = element_rect(color = "black", fill = NA, linewidth = 0),
        plot.title = element_text(hjust = 0.5),
        axis.ticks = element_blank(),
        axis.text = element_text(size = 10, color = "black"),
        axis.text.y = element_text(hjust = 0.5),
        legend.key = element_blank(),
        strip.background = element_blank(),
        text = element_text(family = "sans", size = 10, color = "black"))

#### Figure panel for F1 female body weight uMCW test results ----
fig_panel_F1_BW_f <- ggplot(copy(uMCW_mouse_results)[trait == "BW" & generation == "F1" & sex
== "Female", ],
                           aes(x = factor(variable,
                                          levels = c("T3", "T4", "T5", "T6", "T7", "T8", "T9",
"T10", "Euth.")),
                               y = factor(condition_contrast,
                                          levels = c("TWD-DMSO", "IAS-DMSO", "50TBT-DMSO",
"5TBT-DMSO"),
                                          labels = c("TWD (N=14)\nvs\nDMSO (N=20)",
                                                     "IAS (N=15)\nvs\nDMSO (N=20)",
                                                     "50TBT (N=19)\nvs\nDMSO (N=20)",
                                                     "5TBT (N=13)\nvs\nDMSO (N=20)")))) +
  geom_tile(aes(fill = observed_BI), colour = "black", size = 0.25) +
  scale_fill_gradientn(colours = c("blue", "white", "red"),
                       values = rescale(c(-1, 0, 1)),
```

```
                     limits = c(-1, 1)) +
  geom_point(aes(shape = factor(significance)), size = 5, na.rm = TRUE) +
  scale_shape_manual(labels = c("Plower < 0.05", "Pupper < 0.05"),
                     values = c("1" = "\u25B2", "-1" = "\u25BC"),
                     limits = c(-1, 1)) +
  labs(tag = "A",
       title = "Female bodyweight",
       x = "Timepoint",
       y = NULL,
       fill = "Bias index",
       shape = "Significance") +
  guides(fill = guide_colorbar(order = 1), shape = guide_legend(override.aes = list(size = 5),
order = 2)) +
  coord_fixed(ratio = 1) +
  theme(panel.background = element_blank(),
        panel.border = element_rect(color = "black", fill = NA, linewidth = 0),
        plot.title = element_text(hjust = 0.5),
        axis.ticks = element_blank(),
        axis.text = element_text(size = 10, color = "black"),
        axis.text.y = element_text(hjust = 0.5),
        legend.key = element_blank(),
        strip.background = element_blank(),
        text = element_text(family = "sans", size = 10, color = "black"))

#### Figure panel for F1 male body weight uMCW test results ----
fig_panel_F1_BW_m <- ggplot(copy(uMCW_mouse_results)[trait == "BW" & generation == "F1" & sex
== "Male", ],
                            aes(x = factor(variable,
                                           levels = c("T3", "T4", "T5", "T6", "T7", "T8", "T9",
"T10", "Euth.")),
                                y = factor(condition_contrast,
                                           levels = c("TWD-DMSO", "IAS-DMSO", "50TBT-DMSO",
"5TBT-DMSO"),
                                           labels = c("TWD (N=10)\nvs\nDMSO (N=21)",
                                                      "IAS (N=16)\nvs\nDMSO (N=21)",
                                                      "50TBT (N=20)\nvs\nDMSO (N=21)",
                                                      "5TBT (N=15)\nvs\nDMSO (N=21)")))) +
  geom_tile(aes(fill = observed_BI), colour = "black", size = 0.25) +
  scale_fill_gradientn(colours = c("blue", "white", "red"),
                       values = rescale(c(-1, 0, 1)),
                       limits = c(-1, 1)) +
  geom_point(aes(shape = factor(significance)), size = 5, na.rm = TRUE) +
  scale_shape_manual(labels = c("Plower < 0.05", "Pupper < 0.05"),
                     values = c("1" = "\u25B2", "-1" = "\u25BC"),
                     limits = c(-1, 1)) +
  labs(tag = "A",
       title = "Male bodyweight",
       x = "Timepoint",
       y = NULL,
       fill = "Bias index",
       shape = "Significance") +
  guides(fill = guide_colorbar(order = 1), shape = guide_legend(override.aes = list(size = 5),
order = 2)) +
  coord_fixed(ratio = 1) +
  theme(panel.background = element_blank(),
        panel.border = element_rect(color = "black", fill = NA, linewidth = 0),
        plot.title = element_text(hjust = 0.5),
        axis.ticks = element_blank(),
        axis.text = element_text(size = 10, color = "black"),
        axis.text.y = element_text(hjust = 0.5),
        legend.key = element_blank(),
        strip.background = element_blank(),
        text = element_text(family = "sans", size = 10, color = "black"))

#### Figure panel for F1 female fasting body weight mbMCW test results ----
fig_panel_F1_fBW_f <- ggplot(copy(mbMCW_mouse_results)[trait == "fBW" & generation == "F1" &
sex == "Female", ],
                             aes(x = factor(variable,
                                            levels = c("T3", "T4", "T5", "T6", "T7", "T8",
```

```
"T9", "T10", "Euth.")),
                                 y = factor(unmatched_condition_contrast,
                                            levels = c("TWD-DMSO", "IAS-DMSO", "50TBT-DMSO",
"5TBT-DMSO"),
                                            labels = c("TWD (N=14, 10)\nvs\nDMSO (N=20, 10)",
                                                       "IAS (N=15, 10)\nvs\nDMSO (N=20, 10)",
                                                       "50TBT (N=19, 10)\nvs\nDMSO (N=20, 10)",
                                                       "5TBT (N=13, 10)\nvs\nDMSO (N=20,
10)")))) +
  geom_tile(aes(fill = observed_BI), colour = "black", size = 0.25) +
  scale_fill_gradientn(colours = c("blue", "white", "red"),
                       values = rescale(c(-1, 0, 1)),
                       limits = c(-1, 1)) +
  geom_point(aes(shape = factor(significance)), size = 5, na.rm = TRUE) +
  scale_shape_manual(labels = c("Plower < 0.05", "Pupper < 0.05"),
                     values = c("1" = "\u25B2", "-1" = "\u25BC"),
                     limits = c(-1, 1)) +
  labs(tag = "A",
       title = "Female fasting bodyweight",
       x = "Timepoint",
       y = NULL,
       fill = "Bias index",
       shape = "Significance") +
  guides(fill = guide_colorbar(order = 1), shape = guide_legend(override.aes = list(size = 5),
order = 2)) +
  coord_fixed(ratio = 1) +
  theme(panel.background = element_blank(),
        panel.border = element_rect(color = "black", fill = NA, linewidth = 0),
        plot.title = element_text(hjust = 1),
        axis.ticks = element_blank(),
        axis.text = element_text(size = 10, color = "black"),
        axis.text.y = element_text(hjust = 0.5),
        legend.key = element_blank(),
        strip.background = element_blank(),
        text = element_text(family = "sans", size = 10, color = "black"))

#### Figure panel for F1 male fasting body weight mbMCW test results ----
fig_panel_F1_fBW_m <- ggplot(copy(mbMCW_mouse_results)[trait == "fBW" & generation == "F1" &
sex == "Male", ],
                             aes(x = factor(variable,
                                            levels = c("T3", "T4", "T5", "T6", "T7", "T8",
"T9", "T10", "Euth.")),
                                 y = factor(unmatched_condition_contrast,
                                            levels = c("TWD-DMSO", "IAS-DMSO", "50TBT-DMSO",
"5TBT-DMSO"),
                                            labels = c("TWD (N=10, 10)\nvs\nDMSO (N=21, 10)",
                                                       "IAS (N=16, 10)\nvs\nDMSO (N=21, 10)",
                                                       "50TBT (N=20, 10)\nvs\nDMSO (N=21, 10)",
                                                       "5TBT (N=15, 10)\nvs\nDMSO (N=21,
10)")))) +
  geom_tile(aes(fill = observed_BI), colour = "black", size = 0.25) +
  scale_fill_gradientn(colours = c("blue", "white", "red"),
                       values = rescale(c(-1, 0, 1)),
                       limits = c(-1, 1)) +
  geom_point(aes(shape = factor(significance)), size = 5, na.rm = TRUE) +
  scale_shape_manual(labels = c("Plower < 0.05", "Pupper < 0.05"),
                     values = c("1" = "\u25B2", "-1" = "\u25BC"),
                     limits = c(-1, 1)) +
  labs(tag = "A",
       title = "Male fasting bodyweight",
       x = "Timepoint",
       y = NULL,
       fill = "Bias index",
       shape = "Significance") +
  guides(fill = guide_colorbar(order = 1), shape = guide_legend(override.aes = list(size = 5),
order = 2)) +
  coord_fixed(ratio = 1) +
  theme(panel.background = element_blank(),
        panel.border = element_rect(color = "black", fill = NA, linewidth = 0),
```

```
        plot.title = element_text(hjust = 1),
        axis.ticks = element_blank(),
        axis.text = element_text(size = 10, color = "black"),
        axis.text.y = element_text(hjust = 0.5),
        legend.key = element_blank(),
        strip.background = element_blank(),
        text = element_text(family = "sans", size = 10, color = "black"))

#### Figure panel for F1 female fasting glucose uMCW test results ----
fig_panel_F1_fglucose_f <- ggplot(copy(uMCW_mouse_results)[trait == "fglucose" & generation ==
"F1" & sex == "Female", ],
                                  aes(x = factor(variable,
                                                 levels = c("T3", "T4", "T5", "T6", "T7", "T8",
"T9", "T10", "Euth.")),
                                      y = factor(condition_contrast,
                                                 levels = c("TWD-DMSO", "IAS-DMSO", "50TBT-
DMSO", "5TBT-DMSO"),
                                                 labels = c("TWD (N=14, 10)\nvs\nDMSO (N=20,
10)",
                                                            "IAS (N=15, 10)\nvs\nDMSO (N=20,
10)",
                                                            "50TBT (N=19, 10)\nvs\nDMSO (N=20,
10)",
                                                            "5TBT (N=13, 10)\nvs\nDMSO (N=20,
10)")))) +
  geom_tile(aes(fill = observed_BI), colour = "black", size = 0.25) +
  scale_fill_gradientn(colours = c("blue", "white", "red"),
                       values = rescale(c(-1, 0, 1)),
                       limits = c(-1, 1)) +
  geom_point(aes(shape = factor(significance)), size = 5, na.rm = TRUE) +
  scale_shape_manual(labels = c("Plower < 0.05", "Pupper < 0.05"),
                     values = c("1" = "\u25B2", "-1" = "\u25BC"),
                     limits = c(-1, 1)) +
  labs(tag = "A",
       title = "Female fasting glucose",
       x = "Timepoint",
       y = NULL,
       fill = "Bias index",
       shape = "Significance") +
  guides(fill = guide_colorbar(order = 1), shape = guide_legend(override.aes = list(size = 5),
order = 2)) +
  coord_fixed(ratio = 1) +
  theme(panel.background = element_blank(),
        panel.border = element_rect(color = "black", fill = NA, linewidth = 0),
        plot.title = element_text(hjust = 1),
        axis.ticks = element_blank(),
        axis.text = element_text(size = 10, color = "black"),
        axis.text.y = element_text(hjust = 0.5),
        legend.key = element_blank(),
        strip.background = element_blank(),
        text = element_text(family = "sans", size = 10, color = "black"))

#### Figure panel for F1 male fasting glucose uMCW test results ----
fig_panel_F1_fglucose_m <- ggplot(copy(uMCW_mouse_results)[trait == "fglucose" & generation ==
"F1" & sex == "Male", ],
                                  aes(x = factor(variable,
                                                 levels = c("T3", "T4", "T5", "T6", "T7", "T8",
"T9", "T10", "Euth.")),
                                      y = factor(condition_contrast,
                                                 levels = c("TWD-DMSO", "IAS-DMSO", "50TBT-
DMSO", "5TBT-DMSO"),
                                                 labels = c("TWD (N=10, 10)\nvs\nDMSO (N=21,
10)",
                                                            "IAS (N=16, 10)\nvs\nDMSO (N=21,
10)",
                                                            "50TBT (N=20, 10)\nvs\nDMSO (N=21,
10)",
                                                            "5TBT (N=15, 10)\nvs\nDMSO (N=21,
10)")))) +
```

```
  geom_tile(aes(fill = observed_BI), colour = "black", size = 0.25) +
  scale_fill_gradientn(colours = c("blue", "white", "red"),
                       values = rescale(c(-1, 0, 1)),
                       limits = c(-1, 1)) +
  geom_point(aes(shape = factor(significance)), size = 5, na.rm = TRUE) +
  scale_shape_manual(labels = c("Plower < 0.05", "Pupper < 0.05"),
                     values = c("1" = "\u25B2", "-1" = "\u25BC"),
                     limits = c(-1, 1)) +
  labs(tag = "A",
       title = "Male fasting glucose",
       x = "Timepoint",
       y = NULL,
       fill = "Bias index",
       shape = "Significance") +
  guides(fill = guide_colorbar(order = 1), shape = guide_legend(override.aes = list(size = 5),
order = 2)) +
  coord_fixed(ratio = 1) +
  theme(panel.background = element_blank(),
        panel.border = element_rect(color = "black", fill = NA, linewidth = 0),
        plot.title = element_text(hjust = 1),
        axis.ticks = element_blank(),
        axis.text = element_text(size = 10, color = "black"),
        axis.text.y = element_text(hjust = 0.5),
        legend.key = element_blank(),
        strip.background = element_blank(),
        text = element_text(family = "sans", size = 10, color = "black"))

#### Figure panel for F1 female tissue weight uMCW test results ----
fig_panel_F1_tissues_f <- ggplot(copy(uMCW_mouse_results)[(trait == "liver" | trait == "iWAT" |
trait == "gWAT") & generation == "F1" & sex == "Female", ],
                                 aes(x = factor(trait,
                                                levels = c("liver", "iWAT", "gWAT"), labels =
c("Liver", "iWAT", "gWAT")),
                                     y = factor(condition_contrast,
                                                levels = c("TWD-DMSO", "IAS-DMSO", "50TBT-
DMSO", "5TBT-DMSO"),
                                                labels = c("TWD (N=10)\nvs\nDMSO (N=10)",
                                                           "IAS (N=10)\nvs\nDMSO (N=10)",
                                                           "50TBT (N=10)\nvs\nDMSO (N=10)",
                                                           "5TBT (N=10)\nvs\nDMSO (N=10)")))) +
  geom_tile(aes(fill = observed_BI), colour = "black", size = 0.25) +
  scale_fill_gradientn(colours = c("blue", "white", "red"),
                       values = rescale(c(-1, 0, 1)),
                       limits = c(-1, 1)) +
  geom_point(aes(shape = factor(significance)), size = 5, na.rm = TRUE) +
  scale_shape_manual(labels = c("Plower < 0.05", "Pupper < 0.05"),
                     values = c("1" = "\u25B2", "-1" = "\u25BC"),
                     limits = c(-1, 1)) +
  labs(tag = "A",
       title = "Female tissue weight",
       x = "Tissue",
       y = NULL,
       fill = "Bias index",
       shape = "Significance") +
  guides(fill = guide_colorbar(order = 1), shape = guide_legend(override.aes = list(size = 5),
order = 2)) +
  coord_fixed(ratio = 1) +
  theme(panel.background = element_blank(),
        panel.border = element_rect(color = "black", fill = NA, linewidth = 0),
        plot.title = element_text(hjust = 0.5),
        axis.ticks = element_blank(),
        axis.text = element_text(size = 10, color = "black"),
        axis.text.y = element_text(hjust = 0.5),
        legend.key = element_blank(),
        strip.background = element_blank(),
        text = element_text(family = "sans", size = 10, color = "black"))

#### Figure panel for F1 male tissue weight uMCW test results ----
fig_panel_F1_tissues_m <- ggplot(copy(uMCW_mouse_results)[(trait == "liver" | trait == "iWAT" |
```

```
trait == "gWAT") & generation == "F1" & sex == "Male", ],
                                       aes(x = factor(trait,
                                                      levels = c("liver", "iWAT", "gWAT"), labels =
c("Liver", "iWAT", "gWAT")),
                                           y = factor(condition_contrast,
                                                      levels = c("TWD-DMSO", "IAS-DMSO", "50TBT-
DMSO", "5TBT-DMSO"),
                                                      labels = c("TWD (N=10)\nvs\nDMSO (N=10)",
                                                                 "IAS (N=10)\nvs\nDMSO (N=10)",
                                                                 "50TBT (N=10)\nvs\nDMSO (N=10)",
                                                                 "5TBT (N=10)\nvs\nDMSO (N=10)")))) +
  geom_tile(aes(fill = observed_BI), colour = "black", size = 0.25) +
  scale_fill_gradientn(colours = c("blue", "white", "red"),
                       values = rescale(c(-1, 0, 1)),
                       limits = c(-1, 1)) +
  geom_point(aes(shape = factor(significance)), size = 5, na.rm = TRUE) +
  scale_shape_manual(labels = c("Plower < 0.05", "Pupper < 0.05"),
                     values = c("1" = "\u25B2", "-1" = "\u25BC"),
                     limits = c(-1, 1)) +
  labs(tag = "A",
       title = "Male tissue weight",
       x = "Tissue",
       y = NULL,
       fill = "Bias index",
       shape = "Significance") +
  guides(fill = guide_colorbar(order = 1), shape = guide_legend(override.aes = list(size = 5),
order = 2)) +
  coord_fixed(ratio = 1) +
  theme(panel.background = element_blank(),
        panel.border = element_rect(color = "black", fill = NA, linewidth = 0),
        plot.title = element_text(hjust = 0.5),
        axis.ticks = element_blank(),
        axis.text = element_text(size = 10, color = "black"),
        axis.text.y = element_text(hjust = 0.5),
        legend.key = element_blank(),
        strip.background = element_blank(),
        text = element_text(family = "sans", size = 10, color = "black"))

#### Figure panel for F1 female plasma metabolite levels uMCW test results ----
fig_panel_F1_metabolites_f <- ggplot(copy(uMCW_mouse_results)[(trait == "Amylin" | trait ==
"GIP" | trait == "GLP-1" | trait == "Ghrelin" | trait == "Insulin" | trait == "Leptin" | trait
== "PYY" | trait == "Glucagon" | trait == "PP" | trait == "Resistin" | trait == "C-Peptide") &
generation == "F1" & sex == "Female", ],
                                           aes(x = factor(trait,
                                                          levels = c("Amylin", "GIP", "Ghrelin",
"GLP-1", "Insulin", "Leptin", "PYY", "Glucagon", "PP", "Resistin", "C-Peptide")),
                                               y = factor(condition_contrast,
                                                          levels = c("TWD-DMSO", "IAS-DMSO", "50TBT-
DMSO", "5TBT-DMSO"),
                                                          labels = c("TWD (N=10)\nvs\nDMSO (N=10)",
                                                                     "IAS (N=10)\nvs\nDMSO (N=10)",
                                                                     "50TBT (N=10)\nvs\nDMSO (N=10)",
                                                                     "5TBT (N=10)\nvs\nDMSO
(N=10)")))) +
  geom_tile(aes(fill = observed_BI), colour = "black", size = 0.25) +
  scale_fill_gradientn(colours = c("blue", "white", "red"),
                       values = rescale(c(-1, 0, 1)),
                       limits = c(-1, 1)) +
  geom_point(aes(shape = factor(significance)), size = 5, na.rm = TRUE) +
  scale_shape_manual(labels = c("Plower < 0.05", "Pupper < 0.05"),
                     values = c("1" = "\u25B2", "-1" = "\u25BC"),
                     limits = c(-1, 1)) +
  labs(tag = "A",
       title = "Female plasma metabolites",
       x = "Metabolite",
       y = NULL,
       fill = "Bias index",
       shape = "Significance") +
  guides(fill = guide_colorbar(order = 1), shape = guide_legend(override.aes = list(size = 5),
```

```
order = 2)) +
  coord_fixed(ratio = 1) +
  theme(panel.background = element_blank(),
        panel.border = element_rect(color = "black", fill = NA, linewidth = 0),
        plot.title = element_text(hjust = 0.5),
        axis.ticks = element_blank(),
        axis.text = element_text(size = 10, color = "black"),
        axis.text.x = element_text(angle = 90, hjust = 1, vjust = 0.5),
        axis.text.y = element_text(hjust = 0.5),
        legend.key = element_blank(),
        strip.background = element_blank(),
        text = element_text(family = "sans", size = 10, color = "black"))

#### Figure panel for F1 male plasma metabolite levels uMCW test results ----
fig_panel_F1_metabolites_m <- ggplot(copy(uMCW_mouse_results)[(trait == "Amylin" | trait ==
"GIP" | trait == "GLP-1" | trait == "Ghrelin" | trait == "Insulin" | trait == "Leptin" | trait
== "PYY" | trait == "Glucagon" | trait == "PP" | trait == "Resistin" | trait == "C-Peptide") &
generation == "F1" & sex == "Male", ],
                                     aes(x = factor(trait,
                                                    levels = c("Amylin", "GIP", "Ghrelin",
"GLP-1", "Insulin", "Leptin", "PYY", "Glucagon", "PP", "Resistin", "C-Peptide")),
                                         y = factor(condition_contrast,
                                                    levels = c("TWD-DMSO", "IAS-DMSO", "50TBT-
DMSO", "5TBT-DMSO"),
                                                    labels = c("TWD (N=10)\nvs\nDMSO (N=10)",
                                                               "IAS (N=10)\nvs\nDMSO (N=10)",
                                                               "50TBT (N=10)\nvs\nDMSO (N=10)",
                                                               "5TBT (N=10)\nvs\nDMSO
(N=10)")))) +
  geom_tile(aes(fill = observed_BI), colour = "black", size = 0.25) +
  scale_fill_gradientn(colours = c("blue", "white", "red"),
                       values = rescale(c(-1, 0, 1)),
                       limits = c(-1, 1)) +
  geom_point(aes(shape = factor(significance)), size = 5, na.rm = TRUE) +
  scale_shape_manual(labels = c("Plower < 0.05", "Pupper < 0.05"),
                     values = c("1" = "\u25B2", "-1" = "\u25BC"),
                     limits = c(-1, 1)) +
  labs(tag = "A",
       title = "Male plasma metabolites",
       x = "Metabolite",
       y = NULL,
       fill = "Bias index",
       shape = "Significance") +
  guides(fill = guide_colorbar(order = 1), shape = guide_legend(override.aes = list(size = 5),
order = 2)) +
  coord_fixed(ratio = 1) +
  theme(panel.background = element_blank(),
        panel.border = element_rect(color = "black", fill = NA, linewidth = 0),
        plot.title = element_text(hjust = 0.5),
        axis.ticks = element_blank(),
        axis.text = element_text(size = 10, color = "black"),
        axis.text.x = element_text(angle = 90, hjust = 1, vjust = 0.5),
        axis.text.y = element_text(hjust = 0.5),
        legend.key = element_blank(),
        strip.background = element_blank(),
        text = element_text(family = "sans", size = 10, color = "black"))

## 4.2. Principal Component Analysis (PCA) of plasma metabolite data ----
### 4.2.a. Preparing data ----
##### Selecting metabolites with data for all mice per sex ----
PCA_F1_plasma <- unique(copy(uMCW_F1_plasma)[
  , .(trait, sex, element_ID, condition, value)])[
    , .(N = .N), by = .(trait, sex, condition)][
      N == 10, ][
        , .(N = .N), by = .(trait, sex)][
          N == 5, ]

#### Executing F1 female PCA ----
PCA_F1_plasma_f <- setorder(dcast(unique(copy(uMCW_F1_plasma)[
```

```
  trait %in% copy(PCA_F1_plasma)[sex == "Female", ][
    , .(trait)][[1]] & sex == "Female", ][
      , .(trait, element_ID, condition, value)]),
  element_ID + condition ~ trait, value.var = "value")[
    , `:=`(order, factor(condition, levels = c("DMSO", "5TBT", "50TBT", "IAS", "TWD")))],
  order, element_ID)
PCA_F1_plasma_f_prcomp <- prcomp(x = as.matrix(copy(PCA_F1_plasma_f)[
  , `:=`(condition = NULL, order = NULL)], rownames = TRUE), scale = TRUE)
PCA_F1_plasma_f_eig <- fviz_eig(PCA_F1_plasma_f_prcomp)

#### Executing F1 male PCA ----
PCA_F1_plasma_m <- setorder(dcast(unique(copy(uMCW_F1_plasma)[
  trait %in% copy(PCA_F1_plasma)[sex == "Male", ][
    , .(trait)][[1]] & sex == "Male", ][
      , .(trait, element_ID, condition, value)]),
  element_ID + condition ~ trait, value.var = "value")[
    , `:=`(order, factor(condition, levels = c("DMSO", "5TBT", "50TBT", "IAS", "TWD")))],
  order, element_ID)
PCA_F1_plasma_m_prcomp <- prcomp(x = as.matrix(copy(PCA_F1_plasma_m)[
  , `:=`(condition = NULL, order = NULL)], rownames = TRUE), scale = TRUE)
PCA_F1_plasma_m_eig <- fviz_eig(PCA_F1_plasma_m_prcomp)

### 4.2.b. Drawing figure panels with PCA results ----
#### Figure panel for F1 female PCA results ----
fig_panel_PCA_F1_plasma_f_biplot <- fviz_pca(PCA_F1_plasma_f_prcomp,
                                             habillage =
factor(copy(PCA_F1_plasma_f)$condition,
                                                                levels = c("DMSO", "5TBT",
"50TBT", "IAS", "TWD"),
                                                                labels = c("DMSO (N=10)", "5TBT
(N=10)", "50TBT (N=10)", "IAS (N=10)", "TWD (N=10)")),
                                             repel = TRUE, pointsize = 3, label = "var",
col.var = "black", legend.title = "Exposure group", invisible = "quali") +
  scale_shape_manual(values = c(19,19,19,19,19)) +
  scale_color_manual(values = c("blue", "orange", "red", "purple", "yellowgreen")) +
  labs(tag = "A", title = "Female plasma metabolites") +
  theme(aspect.ratio = 0.5,
        panel.border = element_rect(color = "black", fill = NA, linewidth = 1),
        plot.title = element_text(hjust = 0.5))

#### Figure panel with F1 male plasma PCA results ----
fig_panel_PCA_F1_plasma_m_biplot <- fviz_pca(PCA_F1_plasma_m_prcomp,
                                             habillage =
factor(copy(PCA_F1_plasma_m)$condition,
                                                                levels = c("DMSO", "5TBT",
"50TBT", "IAS", "TWD"),
                                                                labels = c("DMSO (N=10)", "5TBT
(N=10)", "50TBT (N=10)", "IAS (N=10)", "TWD (N=10)")),
                                             repel = TRUE, pointsize = 3, label = "var",
col.var = "black", legend.title = "Exposure group", invisible = "quali") +
  scale_shape_manual(values = c(19,19,19,19,19)) +
  scale_color_manual(values = c("blue", "orange", "red", "purple", "yellowgreen")) +
  labs(tag = "A", title = "Male plasma metabolites") +
  theme(aspect.ratio = 0.5,
        panel.border = element_rect(color = "black", fill = NA, linewidth = 1),
        plot.title = element_text(hjust = 0.5))

# Saving RData file
save.image(file = "2026_Diaz_Castillo_et_al.RData")

# 5. ANALYSIS OF DIFFERENTIAL GENE EXPRESSION ----
# This section will determine large scale differences in gene expression between female and
male, GWAT and liver samples for all experimental groups (5.1), and will determine significant
changes in gene expression (5.2) and functional enrichments for the most pronounced changes in
gene expression (5.3) between exposure groups and controls in each sex and tissue.
## 5.1. Gene expression hierarchical clustering analyses ----
### 5.1.a. Preparing data and annotations ----
clustering_scaled_normalized_reads <- t(apply(as.matrix(setorder(fread("Supplementary Data
17.csv")[
```

```
  , c(1, 10:109)][
    , (2:101) := lapply(.SD, function(x) (x/sum(x))*1000000), .SDcols = 2:101][
      , `:=`(sum, rowSums(.SD, na.rm = TRUE)), .SDcols = 2:101],
  -sum)[
    1:10000, ][
      , `:=`(sum, NULL)],
  rownames = 1),
  1, function(x) (x - min(x)) / (max(x) - min(x))))[
    , c("G_F_DMSO_569", "G_F_DMSO_623", "G_F_DMSO_695", "G_F_DMSO_709", "G_F_DMSO_785",
        "G_F_5TBT_637", "G_F_5TBT_645", "G_F_5TBT_724", "G_F_5TBT_727", "G_F_5TBT_820",
        "G_F_50TBT_600", "G_F_50TBT_649", "G_F_50TBT_653", "G_F_50TBT_757", "G_F_50TBT_763",
        "G_F_IAS_608", "G_F_IAS_671",  "G_F_IAS_774",  "G_F_IAS_800",  "G_F_IAS_817",
        "G_F_TWD_616",  "G_F_TWD_680",  "G_F_TWD_684",  "G_F_TWD_687", "G_F_TWD_806",
        "G_M_DMSO_573", "G_M_DMSO_627", "G_M_DMSO_703", "G_M_DMSO_713", "G_M_DMSO_788",
        "G_M_5TBT_639", "G_M_5TBT_646", "G_M_5TBT_726", "G_M_5TBT_733", "G_M_5TBT_822",
        "G_M_50TBT_601", "G_M_50TBT_650", "G_M_50TBT_657", "G_M_50TBT_760", "G_M_50TBT_767",
        "G_M_IAS_612",  "G_M_IAS_673",  "G_M_IAS_779",  "G_M_IAS_804",  "G_M_IAS_818",
        "G_M_TWD_621",  "G_M_TWD_683",  "G_M_TWD_688", "G_M_TWD_689",  "G_M_TWD_808",
        "L_F_DMSO_569", "L_F_DMSO_623", "L_F_DMSO_695", "L_F_DMSO_709", "L_F_DMSO_785",
        "L_F_5TBT_637", "L_F_5TBT_645", "L_F_5TBT_724", "L_F_5TBT_727", "L_F_5TBT_820",
        "L_F_50TBT_600", "L_F_50TBT_649", "L_F_50TBT_653", "L_F_50TBT_757", "L_F_50TBT_763",
        "L_F_IAS_608",  "L_F_IAS_671",  "L_F_IAS_774",  "L_F_IAS_800",  "L_F_IAS_817",
        "L_F_TWD_616",  "L_F_TWD_680", "L_F_TWD_684",  "L_F_TWD_687",  "L_F_TWD_806",
        "L_M_DMSO_573", "L_M_DMSO_627", "L_M_DMSO_703", "L_M_DMSO_713", "L_M_DMSO_788",
        "L_M_5TBT_639", "L_M_5TBT_646", "L_M_5TBT_726", "L_M_5TBT_733", "L_M_5TBT_822",
        "L_M_50TBT_601", "L_M_50TBT_650", "L_M_50TBT_657", "L_M_50TBT_760", "L_M_50TBT_767",
        "L_M_IAS_612",  "L_M_IAS_673",  "L_M_IAS_779",  "L_M_IAS_804",  "L_M_IAS_818",
        "L_M_TWD_621", "L_M_TWD_683",  "L_M_TWD_688",  "L_M_TWD_689",  "L_M_TWD_808")]

clustering_annotation <- setcolorder(subset(data.frame(data.table(sample =
colnames(clustering_scaled_normalized_reads))[
  , `:=`(c("Tissue", "Sex", "Exposure", "mouse_ID"), tstrsplit(sample, "_", fixed = TRUE))][
    , `:=`(Tissue = fcase(Tissue == "G", "GWAT",
                          Tissue == "L", "Liver"),
           Sex = fcase(Sex == "F", "Females",
                       Sex == "M", "Males"))][
                         , `:=`(Exposure, factor(Exposure, levels = c("DMSO", "5TBT", "50TBT",
"IAS", "TWD")))][
                           , .(sample, Exposure, Sex, Tissue)],
  row.names = 1),
  select = (1:3)),
  c("Tissue", "Sex", "Exposure"))

### 5.1.b. Drawing figure panel for hierarchical clustering analysis results ----
fig_panel_clustering <- Heatmap(clustering_scaled_normalized_reads,
                                cluster_columns = FALSE,
                                show_row_names = FALSE,
                                show_column_names = FALSE,
                                col = brewer.pal(9, "Blues"),
                                name = "Scaled\nnormalized\nread counts",
                                use_raster = "TRUE",
                                heatmap_legend_param = list(title_gp = gpar(fontsize = 20,
fontface = "plain"),
                                                            labels_gp = gpar(fontsize = 15,
fontface = "plain")),
                                top_annotation = HeatmapAnnotation(df = clustering_annotation,
                                                                   annotation_name_gp=
gpar(fontsize = 20),
                                                                   annotation_legend_param =
list(title_gp = gpar(fontsize = 20, fontface = "plain"),

labels_gp = gpar(fontsize = 15)),
                                                                   col = list(Exposure =
c("DMSO" = "blue",

"5TBT" = "orange",

"50TBT" = "red",
```

```
"IAS" = "purple",

"TWD" = "yellowgreen"),
                                                                                Sex = c("Females"
= "indianred",
                                                                                        "Males" =
"deepskyblue"),
                                                                                Tissue = c("GWAT"
= "grey4",

"Liver" = "grey"))))

## 5.2. Differential gene expression analyses using uMCW tests ----
#### 5.2.a. Preparing data ----
uMCW_transcriptomic_cpm <- setorder(rbindlist(lapply(split(copy(key_contrast_transcriptomics),
by = "contrast_ID"),
                                                     function(x){
                                                       i <- setnames(fread("Supplementary Data
17.csv"),
                                                                     c("Gene_ID", "Chromosome",
"Start", "End"),
                                                                     c("element_ID",
"element_chr", "element_start", "element_end"))
                                                       ii <- paste0("element|", x$condition_a)
                                                       iii <- paste0("element|", x$condition_b)
                                                       iv <- setnames(data.table(contrast_ID =
x$contrast_ID,
                                                                                 condition_a =
x$condition_a,
                                                                                 condition_b =
x$condition_b,
                                                                                 merge(copy(i)
[, .SD, .SDcols = patterns(ii)],
                                                                                       copy(i)
[, .SD, .SDcols = patterns(iii)])),
                                                                      8:17, c("a.1", "a.2",
"a.3", "a.4", "a.5", "b.1", "b.2", "b.3", "b.4", "b.5"))[
                                                                        , `:=`(filtering,
rowSums(.SD > 1, na.rm = TRUE)), .SDcols = 8:17][
                                                                          filtering > 1, ][
                                                                            , `:=`(filtering,
NULL)]
                                                       v <- rbind(cbind(contrast_type = "long",
                                                                        copy(iv)[, (8:17) :=
lapply(.SD, function(x) (x/sum(x))*1000000), .SDcols = 8:17]),
                                                                  cbind(contrast_type =
"short",
                                                                        copy(iv)[element_chr ==
"chrY" | element_chr %like% "chr.*_.*" | element_chr == "chrM", `:=`(element_chr, "NO")][
                                                                          element_chr != "NO",
][
                                                                            , (8:17) :=
lapply(.SD, function(x) (x/sum(x))*1000000), .SDcols = 8:17]))})),
                                    contrast_type, contrast_ID, element_chr, element_start,
element_end)

### 5.2.b. Writing data tables for uMCW tests to the “temp” subdirectory ----
# Data tables for each contrast will be broken into multiple sections (primarily one per
chromosome) to facilitate the execution of these tests.
invisible(lapply(split(copy(uMCW_transcriptomic_cpm)[
  , `:=`(subset_chr, gsub(".*_.*", "NO", element_chr))], by = c("contrast_type", "contrast_ID",
"subset_chr")),
  function(x){
    contrast <- unique(x[, contrast_ID])
    contrast_type <- unique(x[, contrast_type])
    chr <- unique(x[, subset_chr])
    fwrite(x[, `:=`(subset_chr = NULL)], file = paste0("./temp/contrast_", contrast, "_",
contrast_type, "_", chr, "_uMCWtest_data.csv"), row.names = FALSE)}))
```

```
### 5.2.c. Executing uMCW tests ----
# This step will take a considerable amount of time. The user can monitor the progress by
checking the uMCW result files that are being saved to "temp".
lapply(list.files("./temp", pattern = "^contrast_.*_uMCWtest_data.csv", full.names = TRUE),
       function(x){
         a <- uMCWtest(x, 10000)})

### 5.2.d. Retrieving uMCW results ----
uMCW_transcriptomic_results <- rbindlist(lapply(list.files("./temp/", pattern =
"^contrast_.*_uMCWtest_results.csv$", full.names = TRUE),
                                                function(x){
                                                  i <- fread(x)[
                                                    BI_type == "uMCW_BI" & paste0(condition_a,
"-", condition_b) == condition_contrast, ]}))[
                                                      , `:=`(c("tissue", "sex", "condition_a"),
tstrsplit(condition_a, "_", fixed = TRUE))][
                                                        , `:=`(c("tissue", "sex",
"condition_b"), tstrsplit(condition_b, "_", fixed = TRUE))][
                                                          , `:=`(tissue = fcase(tissue == "G",
"GWAT",
                                                                                tissue == "L",
"Liver"),
                                                                 sex = fcase(sex == "F",
"Females",
                                                                             sex == "M",
"Males"))][
                                                                                ,
`:=`(significance = fcase(pupper < 0.05, "1",

plower < 0.05, "-1",

pmin(pupper, plower) >= 0.05, "NO"))]

### 5.2.e. Writing copy of Supplementary Data 23 with uMCW test results to the "File
confirmations/Supplementary files" subdirectory ----
fwrite(setnames(copy(uMCW_transcriptomic_results)[
  , .(contrast_type, sex, tissue, condition_a, condition_b, condition_contrast, element_ID,
element_chr, element_start, element_end, N, n_a, n_b, test_type, observed_BI,
expected_by_chance_BI_N, pupper, plower)][
    , `:=`(contrast_type = fcase(contrast_type == "long", "Complete",
                                 contrast_type == "short", "Main nuclear chromosomes; no Y"))][
                                   , `:=`(test_type = ifelse(test_type == "exact", "Exact",
"Approximated"))],
  c("Contrast type", "Sex", "Tissue", "Condition a", "Condition b", "Condition contrast", "Gene
ID", "Chromosome", "Start", "End", "N", "N condition a", "N condition b", "uMCW test type",
"Observed uMCW_BI", "N expected-by-chance uMCW_BIs", "Pupper value", "Plower value")),
  file = "./File confirmations/Supplementary files/Supplementary Data 23.csv", row.names =
FALSE)

## 5.3. Functional characterization of differential gene expression ----
### 5.3.a. Executing Gene Set Enrichment Analyses (GSEAs) using Gene Ontology (GO) Biological
Process terms and uMCW test results ----
fgsea_results <-
setnames(setorder(na.omit(merge(rbindlist(lapply(split(uMCW_transcriptomic_results[contrast_type
== "long", ], by = "contrast_ID"),
                                                                  function(x){
                                                                    i <- fgsea(pathways =
split(key_gene_ontology$gene_symbol, key_gene_ontology$gs_name),
                                                                               stats =
with(x[order(-observed_BI)], setNames(observed_BI, element_ID)),
                                                                               eps = 0.0,
                                                                               minSize = 15,
                                                                               maxSize = 500)
                                                                    ii <-
collapsePathways(i[order(pval)][pval < 0.05, ],

split(key_gene_ontology$gene_symbol, key_gene_ontology$gs_name),

with(x[order(-observed_BI)], setNames(observed_BI, element_ID)),
```

```
pval.threshold = 0.05)
                                                                    iii <- cbind(unique(copy(x)
[, .(sex, tissue, condition_a, condition_b, condition_contrast)]),

na.omit(i[pathway %in% ii$mainPathways][order(-NES)])}),
                                                           idcol = "contrast_ID"),
                                                 copy(key_gene_ontology_translator),
                                                 by.x = "pathway", by.y = "GO name capitals
underscore", all.x = TRUE, sort = FALSE))[
                                                   , `:=`(contrast_ID,
as.integer(contrast_ID))],
                                   contrast_ID)[
                                     , .(sex, tissue, condition_a, condition_b,
condition_contrast, `GO name capitals`, size, NES, pval)],
                          c("Sex", "Tissue", "Condition a", "Condition b", "Condition
contrast", "Gene Ontology term", "Size", "Normalized Enrichment Score (NES)", "P value"))

### 5.3.b. Writing copy of Supplementary Data 24 with GSEA results to the “File
confirmations/Supplementary files” subdirectory ----
fwrite(fgsea_results, file = "./File confirmations/Supplementary files/Supplementary Data
24.csv", row.names = FALSE)

### 5.3.c. Drawing figure panel for GSEA results ----
fig_panel_fgsea <- ggplot(setorder(fread("Supplementary Data 24.csv")[
  `Gene Ontology term` %in% unlist(unique(setorder(fread("Supplementary Data 24.csv"), `P
value`, -`Normalized Enrichment Score (NES)`)[
    , .(`Gene Ontology term`)])[1:50, ]), ][
      ,`:=`(pval_log = -log10(`P value`))][
        , `:=`(metabolic_GO = fifelse(`Gene Ontology term` %in% c("AEROBIC ELECTRON TRANSPORT
CHAIN",
                                                                  "AEROBIC RESPIRATION",
                                                                  "CELLULAR RESPIRATION",
                                                                  "ELECTRON TRANSPORT CHAIN",
                                                                  "ENERGY DERIVATION BY
OXIDATION OF ORGANIC COMPOUNDS",
                                                                  "MITOCHONDRIAL GENE
EXPRESSION",
                                                                  "MITOCHONDRIAL RESPIRATORY
CHAIN COMPLEX ASSEMBLY",
                                                                  "MITOCHONDRIAL TRANSLATION",
                                                                  "OXIDATIVE PHOSPHORYLATION",
                                                                  "PROTON MOTIVE FORCE-DRIVEN
ATP SYNTHESIS"),
                                      "YES", "NO"))],
  metabolic_GO, pval_log, -`Normalized Enrichment Score (NES)`)[
    , `:=`(`Gene Ontology term` = fifelse(`Gene Ontology term` %in% c("AEROBIC ELECTRON
TRANSPORT CHAIN",
                                                                      "AEROBIC RESPIRATION",
                                                                      "CELLULAR RESPIRATION",
                                                                      "ELECTRON TRANSPORT
CHAIN",
                                                                      "ENERGY DERIVATION BY
OXIDATION OF ORGANIC COMPOUNDS",
                                                                      "MITOCHONDRIAL GENE
EXPRESSION",
                                                                      "MITOCHONDRIAL
RESPIRATORY CHAIN COMPLEX ASSEMBLY",
                                                                      "MITOCHONDRIAL
TRANSLATION",
                                                                      "OXIDATIVE
PHOSPHORYLATION",
                                                                      "PROTON MOTIVE FORCE-
DRIVEN ATP SYNTHESIS"),
                                          paste0("<span style='color:#FF0000'>", `Gene Ontology
term`, "</span>"),
                                          `Gene Ontology term`))],
  aes(x = factor(`Condition a`,
                 levels = c("5TBT", "50TBT", "IAS", "TWD"),
```

```
                  labels = c("5TBT (n=5)\nvs\nDMSO (n=5)",
                             "50TBT (n=5)\nvs\nDMSO (n=5)",
                             "IAS (n=5)\nvs\nDMSO (n=5)",
                             "TWD (n=5)\nvs\nDMSO (n=5)")),
      y = factor(`Gene Ontology term`, levels = unique(`Gene Ontology term`)))) +
  geom_count(aes(size = pval_log, color = `Normalized Enrichment Score (NES)`)) +
  facet_grid(Tissue ~ Sex, scales = "free_y", space = "free_y") +
  scale_size(range = c(-log10(0.05), 5),
             breaks = c(-log10(0.05), 2, 5, 10, 20, 30, 40),
             labels = c("P = 0.05",
                        "P = 0.01",
                        expression(paste("P = 1 x ", 10^-5)),
                        expression(paste("P = 1 x ", 10^-10)),
                        expression(paste("P = 1 x ", 10^-20)),
                        expression(paste("P = 1 x ", 10^-30)),
                        expression(paste("P = 1 x ", 10^-40))),
             limits = c(-log10(0.05), 40)) +
  scale_color_gradientn(colours = c("blue", "white", "red"),
                        values = rescale(c(-4, 0, 4)),
                        limits = c(-4, 4)) +
  labs(tag = "A", x = "Contrasts", y = "Gene Ontology Term [Biological Process (BP)]", color =
"Normalized\nEnrichment\nScore [NES]", size = "-log10(Pvalue)") +
  theme(panel.background = element_rect(fill = "white", color = "black"),
        panel.grid = element_line(color = "grey90"),
        plot.title = element_text(hjust = 0.5),
        strip.background = element_blank(),
        strip.text = element_text(size = 20),
        axis.text.x = element_text(size = 12, color = "black"),
        axis.text.y = element_markdown(),
        legend.key = element_rect(colour = NA, fill = NA),
        text = element_text(family = "sans", size = 20, color = "black"))

# Saving RData file
save.image(file = "2026_Diaz_Castillo_et_al.RData")

# 6. ANALYSIS OF ISOCHORE COMPOSITION OF CHROMOSOMES AND ISOCHORE DISTRIBUTION OF FUNCTIONALLY
RELATED GENES ----
# This section will determine the isochore composition of the primary nuclear chromosomes
(excluding chromosome Y) and the isochore distribution of genes associated with the same Gene
Ontology (GO) terms for each exposure group versus control contrast.
## 6.1. Figure panel for isochore composition of chromosome 12 ----
fig_panel_chr12_isochore <- ggplot(fread("Supplementary Data 18.csv")[Chromosome == "chr12", ])
+
  geom_rect(aes(xmin = Start,
                xmax = End,
                ymin = 0,
                ymax = `GC%`,
                fill = factor(`Class`, levels = c("L1", "L2", "H1", "H2", "H3")),
                color = factor(Class, levels = c("L1", "L2", "H1", "H2", "H3")))) +
  scale_color_manual(breaks = c("L1", "L2", "H1", "H2", "H3"),
                     values = seq_gradient_pal("lightblue", "navyblue", "Lab")
(seq(0,1,length.out=5))) +
  scale_fill_manual(breaks = c("L1", "L2", "H1", "H2", "H3"),
                    values = seq_gradient_pal("lightblue", "navyblue", "Lab")
(seq(0,1,length.out=5))) +
  labs(tag = "A",
       title = "Isochore distribution of chromosome *12* in the mouse genome",
       x = "Chromosome position [Mb]",
       y = "GC content [GC%]",
       fill = "Isochore\nclass",
       color = "Isochore\nclass") +
  scale_x_continuous(labels = function(x) x/1000000) +
  theme(panel.background = element_blank(),
        axis.line.x = element_line(color = "black"),
        plot.title = element_markdown(hjust = 0.5),
        text = element_text(family = "sans", size = 15, color = "black"))

## 6.2. Determining isochore composition of all nuclear chromosomes, except Y, and isochore
distribution of genes associated with specific GO terms ----
```

```
### 6.2.a. Preparing data ----
isochore_chromosome_intersection <- merge(copy(uMCW_transcriptomic_results)[contrast_type ==
"short", ],
                                          fread("Supplementary Data 17.csv")[, 1:9],
                                          by.x = "element_ID", by.y = "Gene_ID", all.x = TRUE,
sort = FALSE)

### 6.2.b. Calculating isochore distributions ----
isochore_distributions <- rbindlist(lapply(split(melt(setnames(setnafill(
  rbind(Reduce(function(x, y) merge(x, y, by = c("sex", "tissue", "condition_a", "condition_b",
"condition_contrast"), all = TRUE, sort = FALSE),
               list(all = copy(isochore_chromosome_intersection)[, .(N = .N), by = .(sex,
tissue, condition_a, condition_b, condition_contrast)],
                    L1 = copy(isochore_chromosome_intersection)[, .(L1 = .N), by = .(sex,
tissue, condition_a, condition_b, condition_contrast, `L1 isochore overlap`)][`L1 isochore
overlap` != "NO",],
                    L2 = copy(isochore_chromosome_intersection)[, .(L2 = .N), by = .(sex,
tissue, condition_a, condition_b, condition_contrast, `L2 isochore overlap`)][`L2 isochore
overlap` != "NO",],
                    H1 = copy(isochore_chromosome_intersection)[, .(H1 = .N), by = .(sex,
tissue, condition_a, condition_b, condition_contrast, `H1 isochore overlap`)][`H1 isochore
overlap` != "NO",],
                    H2 = copy(isochore_chromosome_intersection)[, .(H2 = .N), by = .(sex,
tissue, condition_a, condition_b, condition_contrast, `H2 isochore overlap`)][`H2 isochore
overlap` != "NO",],
                    H3 = copy(isochore_chromosome_intersection)[, .(H3 = .N), by = .(sex,
tissue, condition_a, condition_b, condition_contrast, `H3 isochore overlap`)][`H3 isochore
overlap` != "NO",]))[
                      , `:=`(compartment = "all")],
        setnames(Reduce(function(x, y) merge(x, y, by = c("sex", "tissue", "condition_a",
"condition_b", "condition_contrast", "element_chr"), all = TRUE, sort = FALSE),
                        list(all = copy(isochore_chromosome_intersection)[, .(N = .N), by = .
(sex, tissue, condition_a, condition_b, condition_contrast, element_chr)],
                             L1 = copy(isochore_chromosome_intersection)[, .(L1 = .N), by = .
(sex, tissue, condition_a, condition_b, condition_contrast, element_chr, `L1 isochore
overlap`)][`L1 isochore overlap` != "NO",],
                             L2 = copy(isochore_chromosome_intersection)[, .(L2 = .N), by = .
(sex, tissue, condition_a, condition_b, condition_contrast, element_chr, `L2 isochore
overlap`)][`L2 isochore overlap` != "NO",],
                             H1 = copy(isochore_chromosome_intersection)[, .(H1 = .N), by = .
(sex, tissue, condition_a, condition_b, condition_contrast, element_chr, `H1 isochore
overlap`)][`H1 isochore overlap` != "NO",],
                             H2 = copy(isochore_chromosome_intersection)[, .(H2 = .N), by = .
(sex, tissue, condition_a, condition_b, condition_contrast, element_chr, `H2 isochore
overlap`)][`H2 isochore overlap` != "NO",],
                             H3 = copy(isochore_chromosome_intersection)[, .(H3 = .N), by = .
(sex, tissue, condition_a, condition_b, condition_contrast, element_chr, `H3 isochore
overlap`)][`H3 isochore overlap` != "NO",])),
                 "element_chr", "compartment"),
        rbindlist(lapply(split(merge(copy(key_gene_ontology),
                                     copy(key_gene_ontology_translator),
                                     by.x = "gs_name", by.y = "GO name capitals underscore",
all.x = TRUE, sort = FALSE)[
                                       `GO name capitals` %in% c("CELL JUNCTION ASSEMBLY",
                                                                 "REGULATION OF SYNAPSE
STRUCTURE OR ACTIVITY",
                                                                 "POSITIVE REGULATION OF CELL
PROJECTION ORGANIZATION",
                                                                 "MESENCHYME DEVELOPMENT",
                                                                 "AMEBOIDAL-TYPE CELL
MIGRATION",
                                                                 "MITOCHONDRIAL GENE
EXPRESSION",
                                                                 "AEROBIC RESPIRATION",
                                                                 "OXIDATIVE PHOSPHORYLATION",
                                                                 "PROTON MOTIVE FORCE-DRIVEN
ATP SYNTHESIS",
                                                                 "TRANSLATION AT SYNAPSE"), ][
                                                                   , .(gene_symbol, `GO name
```

```
capitals`)],
                               by = "GO name capitals"),
                         function(x){
                           i <- copy(isochore_chromosome_intersection)[
                             element_ID %in% unlist(x[, .(gene_symbol)])][
                               , `:=`(GO_name, unique(x[, .(`GO name capitals`)]))]
                           ii <- setnames(Reduce(function(x, y) merge(x, y, by = c("sex",
"tissue", "condition_a", "condition_b", "condition_contrast", "GO_name"), all = TRUE, sort =
FALSE),
                                                 list(all = copy(i)[, .(N = .N), by = .(sex,
tissue, condition_a, condition_b, condition_contrast, GO_name)],
                                                      L1 = copy(i)[, .(L1 = .N), by = .(sex,
tissue, condition_a, condition_b, condition_contrast, GO_name, `L1 isochore overlap`)][`L1
isochore overlap` != "NO", ],
                                                      L2 = copy(i)[, .(L2 = .N), by = .(sex,
tissue, condition_a, condition_b, condition_contrast, GO_name, `L2 isochore overlap`)][`L2
isochore overlap` != "NO", ],
                                                      H1 = copy(i)[, .(H1 = .N), by = .(sex,
tissue, condition_a, condition_b, condition_contrast, GO_name, `H1 isochore overlap`)][`H1
isochore overlap` != "NO", ],
                                                      H2 = copy(i)[, .(H2 = .N), by = .(sex,
tissue, condition_a, condition_b, condition_contrast, GO_name, `H2 isochore overlap`)][`H2
isochore overlap` != "NO", ],
                                                      H3 = copy(i)[, .(H3 = .N), by = .(sex,
tissue, condition_a, condition_b, condition_contrast, GO_name, `H3 isochore overlap`)][`H3
isochore overlap` != "NO", ])),
                                          "GO_name", "compartment")}))[
                                            , .(sex, tissue, condition_a, condition_b,
condition_contrast, compartment, N, L1, L2, H1, H2, H3)],
  cols = c("N", "L1", "L2", "H1", "H2", "H3"), fill = 0),
  c("L1", "L2", "H1", "H2", "H3"), c("L1_N", "L2_N", "H1_N", "H2_N", "H3_N")),
  measure.vars = patterns("_N"),
  variable.name = "isochore",
  value.name = "isochore_N")[
    , `:=`(isochore_fraction = isochore_N / N)],
  by = c("sex", "tissue", "condition_a", "isochore")),
  function(x){
    i <- copy(x)[
      , `:=`(isochore_fraction_all, copy(x)[compartment == "all", .(isochore_fraction)])][
        compartment != "all", ]}))[
          , `:=`(isochore_fraction_normalized = log10(isochore_fraction /
isochore_fraction_all))][
            , `:=`(isochore_fraction_normalized, gsub("-Inf", 0,
isochore_fraction_normalized))][
              , `:=`(isochore_fraction_normalized, as.numeric(isochore_fraction_normalized))][
                , `:=`(isochore, gsub("_N", "", isochore))]

### 6.2.c. Drawing figure panels for main figures ----
#### Figure panel for the distribution of isochores in each chromosome using G_F_50TBT vs
G_F_DMSO contrast data ----
fig_panel_isochore_chr <- ggplot(copy(isochore_distributions)[substr(compartment, 1, 3) ==
"chr" & condition_contrast == "G_F_50TBT-G_F_DMSO", ],
                                 aes(x = factor(compartment,
                                                levels = c("chrX", "chr16", "chr14", "chr10",
"chr3", "chr5", "chr1", "chr12", "chr15", "chr19", "chr8", "chr18", "chr6", "chr4", "chr2",
"chr13", "chr17", "chr9", "chr11", "chr7"),
                                                labels = c("X", "16", "14", "10", "3", "5",
"1", "12", "15", "19", "8", "18", "6", "4", "2", "13", "17", "9", "11", "7")),
                                     y = factor(isochore, levels = c("H3", "H2", "H1", "L2",
"L1")))) +
  geom_tile(aes(fill = isochore_fraction_normalized), colour = "black", size = 0.25) +
  scale_fill_gradientn(colours = c("blue", "white", "red"),
                       values = rescale(c(-1.2, 0, 1.2)),
                       limits = c(-1.2, 1.2)) +
  labs(tag = "A",
       title = "Gene fraction per isochore class for each chromosome normalized\nby gene
fraction per isochore class for the entire dataset",
       x = "Chromosomes",
       y = "Isochore class",
```

```
      fill = "log(normalized\ngene fraction)") +
  guides(fill = guide_colorbar(order = 1), shape = guide_legend(override.aes = list(size = 5),
order = 2)) +
  coord_fixed(ratio = 1) +
  theme(panel.background = element_blank(),
        panel.border = element_rect(color = "black", fill = NA, linewidth = 0),
        plot.title = element_text(size = 15, hjust = 0.5),
        axis.ticks = element_blank(),
        axis.text = element_text(size = 15, color = "black"),
        axis.text.y = element_text(hjust = 0.5),
        legend.key = element_blank(),
        strip.background = element_blank(),
        text = element_text(family = "sans", size = 15, color = "black"))

#### Figure panel for the isochore distribution of genes associated to specific GO terms using
G_F_50TBT vs DMSO contrast data ----
fig_panel_isochore_GO <- ggplot(copy(isochore_distributions)[
  substr(compartment, 1, 3) != "chr" &
    condition_contrast == "G_F_50TBT-G_F_DMSO" &
    compartment %in% c("CELL JUNCTION ASSEMBLY",
                       "REGULATION OF SYNAPSE STRUCTURE OR ACTIVITY",
                       "POSITIVE REGULATION OF CELL PROJECTION ORGANIZATION",
                       "MESENCHYME DEVELOPMENT",
                       "AMEBOIDAL-TYPE CELL MIGRATION",
                       "MITOCHONDRIAL GENE EXPRESSION",
                       "AEROBIC RESPIRATION",
                       "OXIDATIVE PHOSPHORYLATION",
                       "PROTON MOTIVE FORCE-DRIVEN ATP SYNTHESIS",
                       "TRANSLATION AT SYNAPSE"), ],
  aes(x = factor(isochore, levels = c("L1", "L2", "H1", "H2", "H3")),
      y = factor(compartment,
                 levels = c("CELL JUNCTION ASSEMBLY",
                            "REGULATION OF SYNAPSE STRUCTURE OR ACTIVITY",
                            "POSITIVE REGULATION OF CELL PROJECTION ORGANIZATION",
                            "MESENCHYME DEVELOPMENT",
                            "AMEBOIDAL-TYPE CELL MIGRATION",
                            "MITOCHONDRIAL GENE EXPRESSION",
                            "AEROBIC RESPIRATION",
                            "OXIDATIVE PHOSPHORYLATION",
                            "PROTON MOTIVE FORCE-DRIVEN ATP SYNTHESIS",
                            "TRANSLATION AT SYNAPSE")))) +
  geom_tile(aes(fill = isochore_fraction_normalized), colour = "black", size = 0.25) +
  scale_fill_gradientn(colours = c("blue", "white", "red"),
                       values = rescale(c(-1, 0, 1)),
                       limits = c(-1, 1)) +
  labs(tag = "A",
      title = "Gene fraction per isochore class for each GO term normalized\nby gene fraction
per isochore class for the entire dataset",
      y = NULL,
      x = "Isochore class",
      fill = "log(normalized\ngene fraction)") +
  guides(fill = guide_colorbar(order = 1), shape = guide_legend(override.aes = list(size = 5),
order = 2)) +
  coord_fixed(ratio = 1) +
  theme(panel.background = element_blank(),
        panel.border = element_rect(color = "black", fill = NA, linewidth = 0),
        plot.title = element_text(size = 15, hjust = 1),
        axis.ticks = element_blank(),
        axis.text = element_text(size = 15, color = "black"),
        axis.text.y = element_text(hjust = 1),
        legend.key = element_blank(),
        strip.background = element_blank(),
        text = element_text(family = "sans", size = 15, color = "black"))

### 6.2.d. Writing copy of Supplementary files with isochore distribution results to the “File
confirmations/Supplementary files” and “File confirmations/Figures” subdirectories ----
#### Writing copy of Supplementary Data 26 to the “File confirmations/Supplementary files”
subdirectory ----
fwrite(setnames(setorder(copy(isochore_distributions)[
```

```
  , `:=`(`Gene subset type` = fifelse(compartment %like% "chr", "Chromosome", "GO term"))][
    , .(sex, tissue, condition_a, condition_b, condition_contrast, `Gene subset type`,
isochore, compartment, isochore_fraction_normalized)][
      , `:=`(condition_a = factor(condition_a, levels = c("5TBT", "50TBT", "IAS", "TWD")),
             isochore = factor(isochore, levels = c("L1", "L2", "H1", "H2", "H3")),
             compartment = factor(compartment, levels = c("chr1", "chr2", "chr3", "chr4",
"chr5",
                                                          "chr6", "chr7", "chr8", "chr9",
"chr10",
                                                          "chr11", "chr12", "chr13", "chr14",
"chr15",
                                                          "chr16", "chr17", "chr18", "chr19",
"chrX",
                                                          "AEROBIC RESPIRATION",
                                                          "AMEBOIDAL-TYPE CELL MIGRATION",
                                                          "CELL JUNCTION ASSEMBLY",
                                                          "MESENCHYME DEVELOPMENT",
                                                          "MITOCHONDRIAL GENE EXPRESSION",
                                                          "OXIDATIVE PHOSPHORYLATION",
                                                          "POSITIVE REGULATION OF CELL
PROJECTION ORGANIZATION",
                                                          "PROTON MOTIVE FORCE-DRIVEN ATP
SYNTHESIS",
                                                          "REGULATION OF SYNAPSE STRUCTURE OR
ACTIVITY",
                                                          "TRANSLATION AT SYNAPSE")))],
  sex, tissue, condition_a, compartment, isochore),
  c("compartment", "isochore", "isochore_fraction_normalized"), c("Gene subset", "Isochore
class", "Normalized gene fraction")),
  file = "./File confirmations/Supplementary files/Supplementary Data 26.csv", row.names =
FALSE)

#### Writing copy of Supplementary figure 1 to the “File confirmations/Figures” subdirectory --
--
fig_sup_isochore_chr <- ggplot(copy(isochore_distributions)[substr(compartment, 1, 3) == "chr",
],
                               aes(x = factor(compartment,
                                              levels = c("chrX", "chr16", "chr14", "chr10",
"chr3", "chr5", "chr1", "chr12", "chr15", "chr19", "chr8", "chr18", "chr6", "chr4", "chr2",
"chr13", "chr17", "chr9", "chr11", "chr7"),
                                              labels = c("ChrX", "Chr16", "Chr14", "Chr10",
"Chr3", "Chr5", "Chr1", "Chr12", "Chr15", "Chr19", "Chr8", "Chr18", "Chr6", "Chr4", "Chr2",
"Chr13", "Chr17", "Chr9", "Chr11", "Chr7")),
                                   y = factor(isochore, levels = c("H3", "H2", "H1", "L2",
"L1")))) +
  geom_tile(aes(fill = isochore_fraction_normalized), colour = "black", size = 0.25) +
  scale_fill_gradientn(colours = c("blue", "white", "red"),
                       values = rescale(c(-1.2, 0, 1.2)),
                       limits = c(-1.2, 1.2)) +
  facet_wrap(factor(condition_contrast,
                    levels = c("G_F_5TBT-G_F_DMSO", "G_F_50TBT-G_F_DMSO", "G_F_IAS-G_F_DMSO",
"G_F_TWD-G_F_DMSO",
                               "L_F_5TBT-L_F_DMSO", "L_F_50TBT-L_F_DMSO", "L_F_IAS-L_F_DMSO",
"L_F_TWD-L_F_DMSO",
                               "G_M_5TBT-G_M_DMSO", "G_M_50TBT-G_M_DMSO", "G_M_IAS-G_M_DMSO",
"G_M_TWD-G_M_DMSO",
                               "L_M_5TBT-L_M_DMSO", "L_M_50TBT-L_M_DMSO", "L_M_IAS-L_M_DMSO",
"L_M_TWD-L_M_DMSO"),
                    labels = c("G_F_5TBT vs DMSO", "G_F_50TBT vs DMSO", "G_F_IAS vs DMSO",
"G_F_TWD vs DMSO",
                               "L_F_5TBT vs DMSO", "L_F_50TBT vs DMSO", "L_F_IAS vs DMSO",
"L_F_TWD vs DMSO",
                               "G_M_5TBT vs DMSO", "G_M_50TBT vs DMSO", "G_M_IAS vs DMSO",
"G_M_TWD vs DMSO",
                               "L_M_5TBT vs DMSO", "L_M_50TBT vs DMSO", "L_M_IAS vs DMSO",
"L_M_TWD vs DMSO")) ~ .) +
  labs(title = "Gene fraction per isochore class for each chromosome normalized by gene
fraction per isochore class for the entire dataset",
       x = "Chromosomes",
```

```
      y = "Isochore class",
      fill = "log(normalized\ngene fraction)") +
  guides(fill = guide_colorbar(order = 1), shape = guide_legend(override.aes = list(size = 5),
order = 2)) +
  coord_fixed(ratio = 1) +
  theme(panel.background = element_blank(),
        panel.border = element_rect(color = "black", fill = NA, linewidth = 0),
        plot.title = element_text(hjust = 0.5, size = 20),
        axis.ticks = element_blank(),
        axis.text = element_text(size = 10, color = "black"),
        axis.text.x = element_text(angle = 270, hjust = 0, vjust = 0.5),
        axis.text.y = element_text(hjust = 0.5),
        legend.key = element_blank(),
        strip.background = element_blank(),
        text = element_text(family = "sans", size = 10, color = "black"))

ggsave("./File confirmations/Supplementary files/Supplementary Figure 1.png", plot =
fig_sup_isochore_chr, width = 25, height = 15, dpi = 300)

#### Writing copy of Supplementary figure 2 to the “File confirmations/Figures” subdirectory --
--
fig_sup_isochore_GO <- ggplot(copy(isochore_distributions)[
  substr(compartment, 1, 3) != "chr" & compartment %in% c("CELL JUNCTION ASSEMBLY",
                                                          "REGULATION OF SYNAPSE STRUCTURE OR
ACTIVITY",
                                                          "POSITIVE REGULATION OF CELL
PROJECTION ORGANIZATION",
                                                          "MESENCHYME DEVELOPMENT",
                                                          "AMEBOIDAL-TYPE CELL MIGRATION",
                                                          "MITOCHONDRIAL GENE EXPRESSION",
                                                          "AEROBIC RESPIRATION",
                                                          "OXIDATIVE PHOSPHORYLATION",
                                                          "PROTON MOTIVE FORCE-DRIVEN ATP
SYNTHESIS",
                                                          "TRANSLATION AT SYNAPSE"), ],
  aes(x = factor(compartment,
                 levels = c("CELL JUNCTION ASSEMBLY",
                            "REGULATION OF SYNAPSE STRUCTURE OR ACTIVITY",
                            "POSITIVE REGULATION OF CELL PROJECTION ORGANIZATION",
                            "MESENCHYME DEVELOPMENT",
                            "AMEBOIDAL-TYPE CELL MIGRATION",
                            "MITOCHONDRIAL GENE EXPRESSION",
                            "AEROBIC RESPIRATION",
                            "OXIDATIVE PHOSPHORYLATION",
                            "PROTON MOTIVE FORCE-DRIVEN ATP SYNTHESIS",
                            "TRANSLATION AT SYNAPSE")),
      y = factor(isochore, levels = c("H3", "H2", "H1", "L2", "L1")))) +
  geom_tile(aes(fill = isochore_fraction_normalized), colour = "black", size = 0.25) +
  scale_fill_gradientn(colours = c("blue", "white", "red"),
                       values = rescale(c(-1, 0, 1)),
                       limits = c(-1, 1)) +
  facet_wrap(factor(condition_contrast,
                    levels = c("G_F_5TBT-G_F_DMSO", "G_F_50TBT-G_F_DMSO", "G_F_IAS-G_F_DMSO",
"G_F_TWD-G_F_DMSO",
                               "L_F_5TBT-L_F_DMSO", "L_F_50TBT-L_F_DMSO", "L_F_IAS-L_F_DMSO",
"L_F_TWD-L_F_DMSO",
                               "G_M_5TBT-G_M_DMSO", "G_M_50TBT-G_M_DMSO", "G_M_IAS-G_M_DMSO",
"G_M_TWD-G_M_DMSO",
                               "L_M_5TBT-L_M_DMSO", "L_M_50TBT-L_M_DMSO", "L_M_IAS-L_M_DMSO",
"L_M_TWD-L_M_DMSO"),
                    labels = c("G_F_5TBT vs DMSO", "G_F_50TBT vs DMSO", "G_F_IAS vs DMSO",
"G_F_TWD vs DMSO",
                               "L_F_5TBT vs DMSO", "L_F_50TBT vs DMSO", "L_F_IAS vs DMSO",
"L_F_TWD vs DMSO",
                               "G_M_5TBT vs DMSO", "G_M_50TBT vs DMSO", "G_M_IAS vs DMSO",
"G_M_TWD vs DMSO",
                               "L_M_5TBT vs DMSO", "L_M_50TBT vs DMSO", "L_M_IAS vs DMSO",
"L_M_TWD vs DMSO")) ~ .) +
  labs(title = "Gene fraction per isochore class for each GO term normalized by gene fraction
```

```
per isochore class for the entire dataset",
       x = NULL,
       y = "Isochore class",
       fill = "log(normalized\ngene fraction)") +
  guides(fill = guide_colorbar(order = 1), shape = guide_legend(override.aes = list(size = 5),
order = 2)) +
  coord_fixed(ratio = 1) +
  theme(panel.background = element_blank(),
        panel.border = element_rect(color = "black", fill = NA, linewidth = 0),
        plot.title = element_text(hjust = 0.5, size = 20),
        axis.ticks = element_blank(),
        axis.text = element_text(size = 10, color = "black"),
        axis.text.x = element_text(angle = 270, hjust = 0, vjust = 0.5),
        axis.text.y = element_text(hjust = 0.5),
        legend.key = element_blank(),
        strip.background = element_blank(),
        text = element_text(family = "sans", size = 10, color = "black"))

ggsave("./File confirmations/Supplementary files/Supplementary Figure 2.png", plot =
fig_sup_isochore_GO, width = 25, height = 15, dpi = 300)

# Saving RData file
save.image(file = "2026_Diaz_Castillo_et_al.RData")

# 7. ANALYSIS OF CONCERTED BIASES IN DIFFERENTIAL GENE EXPRESSION ----
# This section will determine the extent of concerted alterations in gene expression for genes
situated within the same isochore class or chromosome across all exposure groups versus control
contrasts.
## 7.1. Biased-measures Monte Carlo Wilcoxon (bMCW) tests ----
### 7.1.a. Preparing data and writing bMCW test data to the “temp” directory ----
fwrite(setnames(merge(copy(uMCW_transcriptomic_results)[contrast_type == "short", ],
                      setnames(fread("Supplementary Data 17.csv")[, 1:9],
                               c("element_ID", "element_chr", "element_start", "element_end",
"subset_L1", "subset_L2", "subset_H1", "subset_H2", "subset_H3")),
                      all.x = TRUE, sort = FALSE)[
                        , `:=`(subset_chr, element_chr)][
                          , .(contrast_ID, sex, tissue, condition_a, condition_b,
condition_contrast, element_ID, subset_L1, subset_L2, subset_H1, subset_H2, subset_H3,
subset_chr, BI_type, observed_BI)],
                c("BI_type", "observed_BI"), c("bias_measure", "bias_value")),
       file = "./temp/bMCWtest_data.csv", row.names = FALSE)

### 7.1.b. Executing bMCW tests ----
# This step will take a considerable amount of time.
bMCW_results <- bMCWtest("./temp/bMCWtest_data.csv", 10000)[
  , `:=`(significance = fcase(pupper < 0.05, "1",
                              plower < 0.05, "-1",
                              pmin(pupper, plower) >= 0.05, "NO"))]

### 7.1.c. Writing copy of Supplementary Data 25 with bMCW test results to the “File
confirmations/Supplementary files” subdirectory ----
fwrite(setnames(setorder(copy(bMCW_results)[
  , `:=`(gene_subset = fcase(subset_type == "wholeset", "Whole transcriptome",
                             subset_type != "wholeset", tested_subset))][
                               , `:=`(test_type = ifelse(test_type == "exact", "Exact",
"Approximated"))][
                                 , `:=`(gene_subset, factor(gene_subset,
                                                            levels = c("Whole transcriptome",
"L1", "L2", "H1", "H2", "H3",
                                                                       "chr1", "chr2", "chr3",
"chr4", "chr5",
                                                                       "chr6", "chr7", "chr8",
"chr9", "chr10",
                                                                       "chr11", "chr12",
"chr13", "chr14", "chr15",
                                                                       "chr16", "chr17",
"chr18", "chr19", "chrX")))],
  contrast_ID, gene_subset)[
    , .(sex, tissue, condition_a, condition_b, condition_contrast, gene_subset, N, n,
```

```
test_type, BI_type, observed_BI, expected_by_chance_BI_N, pupper, plower)],
  c("Sex", "Tissue", "Condition a", "Condition b", "Condition contrast", "Gene subset", "N", "N
gene subset", "bMCW test type", "bMCW BI type", "Observed bMCW_BI", "N expected-by-chance
bMCW_BIs", "Pupper value", "Plower value")),
  file = "./File confirmations/Supplementary files/Supplementary Data 25.csv", row.names =
FALSE)

### 7.1.d. Drawing figure panels for bMCW test results ----
#### Figure panel for whole transcriptome bMCW results ----
fig_panel_bMCW_whole <- ggplot(copy(bMCW_results)[
  subset_type == "wholeset",],
  aes(x = bias_measure,
      y = factor(condition_a,
                 levels = c("TWD", "IAS", "50TBT", "5TBT"),
                 labels = c("TWD (N=5)\nvs\nDMSO (N=5)",
                            "IAS (N=5)\nvs\nDMSO (N=5)",
                            "50TBT (N=5)\nvs\nDMSO (N=5)",
                            "5TBT (N=5)\nvs\nDMSO (N=5)")))) +
  geom_tile(aes(fill = observed_BI), colour = "black", size = 0.25) +
  scale_fill_gradientn(colours = c("blue", "white", "red"),
                       values = rescale(c(-1, 0, 1)),
                       limits = c(-1, 1)) +
  geom_point(aes(shape = factor(significance)), size = 5, na.rm = TRUE) +
  scale_shape_manual(labels = c("Plower < 0.05", "Pupper < 0.05"),
                     values = c("1" = "\u25B2", "-1" = "\u25BC"),
                     limits = c(-1, 1)) +
  facet_grid(. ~ sex + tissue) +
  labs(tag = "A",
       title = "Whole genome expression bias",
       x = NULL,
       y = NULL,
       fill = "Bias index",
       shape = "Significance") +
  guides(fill = guide_colorbar(order = 1), shape = guide_legend(override.aes = list(size = 5),
order = 2)) +
  coord_fixed(ratio = 1) +
  theme(panel.background = element_blank(),
        panel.border = element_rect(color = "black", fill = NA, linewidth = 0),
        plot.title = element_text(hjust = 0.5),
        axis.ticks = element_blank(),
        axis.text = element_text(size = 10, color = "black"),
        axis.text.x = element_blank(),
        axis.text.y = element_text(hjust = 0.5),
        legend.key = element_blank(),
        strip.background = element_blank(),
        text = element_text(family = "sans", size = 15, color = "black"))

#### Figure panel for isochore and chromosome bMCW results ----
fig_panel_bMCW_isochore_chr <- ggplot(copy(bMCW_results)[
  subset_type != "wholeset",][
    subset_type == "subset_chr", `:=`(subset_type = "Chromosomes")][
      subset_type %like% "subset_", `:=`(subset_type = "Isochores")][
        , `:=`(significance = fifelse(pupper < plower, -log10(pupper), log10(plower)))][
          , `:=`(significance, gsub("Inf", -log10(1/10000), significance))][
            , `:=`(significance = as.integer(significance))][
              , `:=`(condition_a, factor(condition_a,
                                         levels = c("5TBT", "50TBT", "IAS", "TWD"),
                                         labels = c("5TBT (N=5)\nvs\nDMSO (N=5)",
                                                    "50TBT (N=5)\nvs\nDMSO (N=5)",
                                                    "IAS (N=5)\nvs\nDMSO (N=5)",
                                                    "TWD (N=5)\nvs\nDMSO (N=5)")))],
  aes(x = factor(tested_subset,
                 levels = c("chr1", "chr2", "chr3", "chr4", "chr5",
                            "chr6", "chr7", "chr8", "chr9", "chr10",
                            "chr11", "chr12", "chr13", "chr14", "chr15",
                            "chr16", "chr17", "chr18", "chr19", "chrX",
                            "L1", "L2", "H1", "H2", "H3"),
                 labels = c("1", "2", "3", "4", "5",
                            "6", "7", "8", "9", "10",
```

```
                                "11", "12", "13", "14", "15",
                                "16", "17", "18", "19", "X",
                                "L1", "L2", "H1", "H2", "H3")),
      y = observed_BI,
      fill = significance,
      colour = significance)) +
  geom_point(size = 5) +
  geom_smooth(aes(group = 1), level = 0, se = FALSE, color = "black") +
  scale_fill_gradientn(colours = c("blue", "grey", "red"),
                       breaks = c(log10(1/10000), log10(0.05), -log10(0.05), -log10(1/10000)),
                       labels = c("Plower = 0.0001", "Plower = 0.05", "Pupper = 0.05", "Pupper
= 0.0001"),
                       limits = c(log10(1/10000), -log10(1/10000))) +
  scale_colour_gradientn(colours = c("blue", "grey", "red"),
                         breaks = c(log10(1/10000), log10(0.05), -log10(0.05), -
log10(1/10000)),
                         labels = c("Plower = 0.0001", "Plower = 0.05", "Pupper = 0.05",
"Pupper = 0.0001"),
                         limits = c(log10(1/10000), -log10(1/10000))) +
  geom_hline(yintercept = 0, linetype = "dashed", color = "grey") +
  facet_grid(tissue + condition_a ~ sex + factor(subset_type,
                                                 levels = c("Isochores", "Chromosomes")),
             scales = "free_x") +
  labs(tag = "A", title = "Gene expression bias per isochore class and chromosome",
       x = NULL,
       y = "Observed bMCW sBIs",
       fill = "Significance\n",
       colour = "Significance\n") +
  theme_classic() +
  theme(plot.title = element_text(size = 20, hjust = 0.5),
        axis.text = element_text(size = 15),
        axis.title = element_text(size = 20),
        strip.text.x = element_text(size = 20),
        strip.text.y = element_text(size = 15),
        strip.background = element_blank(),
        legend.title = element_text(size = 20),
        legend.text = element_text(size = 15))

# 8. ANALYSIS OF SPECIFIC GENES ----
## 8.1. bMCW tests for isochores including genes of the Nduf and Sema gene families ----
### 8.1.a. Preparing data and writing bMCW test data to the “temp” directory ----
fwrite(setnames(Reduce(function(x, y) merge(x, y),
                       lapply(split(fread("Supplementary Data 20.csv")[
                         focal_gene != "Lep", ],
                         by = c("focal_gene", "focal_isochore_class")),
                         function(x){
                           copy(uMCW_transcriptomic_results)[
                             contrast_type == "short", ][
                               , .(contrast_ID, sex, tissue, condition_a, condition_b,
condition_contrast, element_ID, BI_type, observed_BI)][
                                 , `:=`(paste0("subset_", unlist(unique(copy(x)[, .
(focal_gene)])), "_", unlist(unique(copy(x)[, .(focal_isochore_class)]))),
                                        fifelse(element_ID %in% unlist(copy(x)[, .(gene_ID)]),
"YES", "NO"))]})),
               c("BI_type", "observed_BI"), c("bias_measure", "bias_value")),
       file = "./temp/specific_gene_bMCWtest_data.csv", row.names = FALSE)

### 8.1.b. Executing bMCW tests ----
# This step will take a considerable amount of time.
bMCW_specific_genes_results <- bMCWtest("./temp/specific_gene_bMCWtest_data.csv", 10000)[
  subset_type != "wholeset", ]

### 8.1.c. Writing copy of Supplementary Data 27 with bMCW test results to the “File
confirmations/Supplementary files” subdirectory ----
fwrite(setnames(copy(bMCW_specific_genes_results)[
  , `:=`(subset_type, gsub("subset_", "", subset_type))][
    , `:=`(c("focal_gene", "focal_isochore"), tstrsplit(subset_type, "_", fixed = TRUE))][
      , `:=`(test_type = ifelse(test_type == "exact", "Exact", "Approximated"))][
        , .(sex, tissue, condition_a, condition_b, condition_contrast, focal_gene,
```

```
focal_isochore, N, n, test_type, BI_type, observed_BI, expected_by_chance_BI_N, pupper,
plower)],
  c("Sex", "Tissue", "Condition a", "Condition b", "Condition contrast", "Focal gene", "Focal
isochore class", "N", "N gene subset", "bMCW test type", "bMCW BI type", "Observed bMCW_BI", "N
expected-by-chance bMCW_BIs", "Pupper value", "Plower value")),
  file ="./File confirmations/Supplementary files/Supplementary Data 27.csv", row.names =
FALSE)

### 8.1.d. Drawing figure panels that depict uMCW test results for Nduf and Sema genes, and
bMCW test results for isochores that include Nduf and Sema genes, and all isochore classes ----
#### Retrieving MCW test results for Nduf, Sema genes and the isochores where they are located
----
specific_gene_isochore_BIs <- rbind(fread("Supplementary Data 27.csv")[
  , `:=`(`Focal element type` = "Isochore")][
    , .(Sex, Tissue, `Condition a`, `Condition b`, `Condition contrast`, `Focal element type`,
`Focal gene`, `Focal isochore class`, `Observed bMCW_BI`, `Pupper value`, `Plower value`)],
  merge(unique(fread("Supplementary Data 27.csv")[
    , .(`Focal gene`, `Focal isochore class`)]),
    fread("Supplementary Data 23.csv")[
      `Contrast type` != "Complete", ],
    by.x = "Focal gene", by.y = "Gene ID", all.x = TRUE, sort = FALSE)[
      , `:=`(`Focal element type` = "Gene")][
        , .(Sex, Tissue, `Condition a`, `Condition b`, `Condition contrast`, `Focal element
type`, `Focal gene`, `Focal isochore class`, `Observed uMCW_BI`, `Pupper value`, `Plower
value`)],
  fread("Supplementary Data 25.csv")[
    `Gene subset` %in% c("L1", "L2", "H1", "H2", "H3"), ][
      , `:=`(`Focal element type` = "Isochore class",
             `Focal gene` = "none",
             `Focal isochore class` = `Gene subset`)][
               , .(Sex, Tissue, `Condition a`, `Condition b`, `Condition contrast`, `Focal
element type`, `Focal gene`, `Focal isochore class`, `Observed bMCW_BI`, `Pupper value`,
`Plower value`)]
  , use.names = FALSE)[
    , `:=`(trait = fcase(`Focal element type` == "Isochore class", "Isochore\nclass",
                         `Focal element type` == "Gene", paste0(substr(`Focal gene`, 1, 4),
"\n", `Focal element type`),
                         `Focal element type` == "Isochore", paste0(substr(`Focal gene`, 1, 4),
"\n", `Focal element type`)),
           sex_condition = paste0(Sex, " ", `Condition a`))][
             , `:=`(significance = fifelse(`Pupper value` < `Plower value`, -log10(`Pupper
value`), log10(`Plower value`)))][
               , `:=`(significance, gsub("Inf", -log10(1/10000), significance))][
                 , `:=`(significance = as.integer(significance))][
                   , `:=`(trait, factor(trait, levels = c("Nduf\nGene", "Nduf\nIsochore",
"Isochore\nclass", "Sema\nIsochore", "Sema\nGene")))]

#### Drawing figure panel for MCW test results for all GWAT contrasts ----
fig_panel_GWAT_gene_isochore_BIs <- ggplot(copy(specific_gene_isochore_BIs)[Tissue == "GWAT",
],
                                           aes(x = factor(`Focal isochore class`, levels =
c("L1", "L2", "H1", "H2", "H3")),
                                               y = `Observed bMCW_BI`,
                                               fill = significance,
                                               colour = significance)) +
  geom_point(size = 5) +
  geom_smooth(aes(group = 1), level = 0, se = FALSE, color = "black") +
  scale_fill_gradientn(colours = c("blue", "grey", "red"),
                       breaks = c(log10(1/10000), log10(0.05), -log10(0.05), -log10(1/10000)),
                       labels = c("Plower = 0.0001", "Plower = 0.05", "Pupper = 0.05", "Pupper
= 0.0001"),
                       limits = c(log10(1/10000), -log10(1/10000))) +
  scale_colour_gradientn(colours = c("blue", "grey", "red"),
                         breaks = c(log10(1/10000), log10(0.05), -log10(0.05), -
log10(1/10000)),
                         labels = c("Plower = 0.0001", "Plower = 0.05", "Pupper = 0.05",
"Pupper = 0.0001"),
                         limits = c(log10(1/10000), -log10(1/10000))) +
  geom_hline(yintercept = 0, linetype = "dashed", color = "grey") +
```

```
  facet_grid(trait ~ factor(sex_condition, levels = c("Females 5TBT",
                                                      "Females 50TBT",
                                                      "Females IAS",
                                                      "Females TWD",
                                                      "Males 5TBT",
                                                      "Males 50TBT",
                                                      "Males IAS",
                                                      "Males TWD")),
             scales = "free_y") +
  labs(tag = "A",
       title = "gWAT gene expression bias isochore dynamics",
       x = "Isochore class",
       y = "Observed MCW BIs",
       fill = "Significance\n", colour = "Significance\n") +
  theme_classic() +
  theme(plot.title = element_text(size = 20, hjust = 0.5),
        axis.text = element_text(size = 15),
        axis.title = element_text(size = 20),
        strip.text.x = element_text(size = 20),
        strip.text.y = element_text(size = 15),
        legend.title = element_text(size = 20),
        legend.text = element_text(size = 15))

#### Drawing figure panel for MCW test results for all liver contrasts ----
fig_panel_liver_gene_isochore_BIs <- ggplot(copy(specific_gene_isochore_BIs)[Tissue == "Liver",
],
                                            aes(x = factor(`Focal isochore class`, levels =
c("L1", "L2", "H1", "H2", "H3")),
                                                y = `Observed bMCW_BI`,
                                                fill = significance,
                                                colour = significance)) +
  geom_point(size = 5) +
  geom_smooth(aes(group = 1), level = 0, se = FALSE, color = "black") +
  scale_fill_gradientn(colours = c("blue", "grey", "red"),
                       breaks = c(log10(1/10000), log10(0.05), -log10(0.05), -log10(1/10000)),
                       labels = c("Plower = 0.0001", "Plower = 0.05", "Pupper = 0.05", "Pupper
= 0.0001"),
                       limits = c(log10(1/10000), -log10(1/10000))) +
  scale_colour_gradientn(colours = c("blue", "grey", "red"),
                         breaks = c(log10(1/10000), log10(0.05), -log10(0.05), -
log10(1/10000)),
                         labels = c("Plower = 0.0001", "Plower = 0.05", "Pupper = 0.05",
"Pupper = 0.0001"),
                         limits = c(log10(1/10000), -log10(1/10000))) +
  geom_hline(yintercept = 0, linetype = "dashed", color = "grey") +
  facet_grid(trait ~ factor(sex_condition, levels = c("Females 5TBT",
                                                      "Females 50TBT",
                                                      "Females IAS",
                                                      "Females TWD",
                                                      "Males 5TBT",
                                                      "Males 50TBT",
                                                      "Males IAS",
                                                      "Males TWD")),
             scales = "free_y") +
  labs(tag = "A",
       title = "gWAT gene expression bias isochore dynamics",
       x = "Isochore class",
       y = "Observed MCW BIs",
       fill = "Significance\n",
       colour = "Significance\n") +
  theme_classic() +
  theme(plot.title = element_text(size = 20, hjust = 0.5),
        axis.text = element_text(size = 15),
        axis.title = element_text(size = 20),
        strip.text.x = element_text(size = 20),
        strip.text.y = element_text(size = 15),
        legend.title = element_text(size = 20),
        legend.text = element_text(size = 15))
```

```
## 8.2. Leptin analyses ----
### 8.2.a. Retrieving MCW test results for changes in plasma levels of leptin and gene
expression of genes Lep and Lnclep ----
leptin_correlations <- merge(copy(uMCW_mouse_results)[
  trait == "Leptin", ][
    , .(sex, condition_a, condition_b, observed_BI, pupper, plower)][
      , `:=`(sex, fifelse(sex == "Female", "Females", "Males"))],
  dcast(copy(uMCW_transcriptomic_results)[
    contrast_type == "long" & (element_ID == "Lep" | element_ID == "Lnclep"), ][
      , .(sex, condition_a, condition_b, element_ID, observed_BI, pupper, plower)],
    sex + condition_a + condition_b ~ element_ID,
    value.var = c("observed_BI", "pupper", "plower")), sort = FALSE)

### 8.2.b. Drawing figure panels ----
#### Figure panel for uMCW test results for changes in expression of genes within the isochore
where leptin is located ----
fig_panel_Lep_isochore <- ggplot(copy(uMCW_transcriptomic_results)[
  contrast_type == "short", ][
    element_ID %in% unlist(fread("Supplementary Data 20.csv")[
      focal_gene == "Lep", ][
        , .(gene_ID)]), ][
          , `:=`(condition_a = fcase(condition_a == "5TBT", "5TBT (N=5)\nvs\nDMSO (N=5)",
                                     condition_a == "50TBT", "50TBT (N=5)\nvs\nDMSO (N=5)",
                                     condition_a == "IAS", "IAS (N=5)\nvs\nDMSO (N=5)",
                                     condition_a == "TWD", "TWD (N=5)\nvs\nDMSO (N=5)"))][
                                       , `:=`(significance = fcase(pupper < 0.05, "1",
                                                                   plower < 0.05, "-1",
                                                                   pmin(pupper, plower) >=
0.05, "NO"))],
  aes(x = factor(element_ID,
                 levels = c("Gm53277", "Gm38779", "Gm40357", "LOC118567373", "Mir129-1",
                            "Lnclep", "Lep", "Gm25589", "Rbm28", "Prrt4",
                            "Impdh1", "Gm26021", "Gm40358", "Hilpda", "Garin1a",
                            "Garin1b", "Calu", "Opn1sw", "n-TPagg6", "Ccdc136",
                            "Flnc", "Atp6v1f", "Spmip1", "Kcp", "Gm26627", "Irf5")),
      y = factor(condition_a,
                 levels = c("TWD (N=5)\nvs\nDMSO (N=5)",
                            "IAS (N=5)\nvs\nDMSO (N=5)",
                            "50TBT (N=5)\nvs\nDMSO (N=5)",
                            "5TBT (N=5)\nvs\nDMSO (N=5)")))) +
  geom_tile(aes(fill = observed_BI), colour = "black", size = 0.25) +
  scale_fill_gradientn(colours = c("blue", "white", "red"),
                       values = rescale(c(-1, 0, 1)),
                       limits = c(-1, 1)) +
  geom_point(aes(shape = factor(significance)), size = 5, na.rm = TRUE) +
  scale_shape_manual(labels = c("Plower < 0.05", "Pupper < 0.05"),
                     values = c("1" = "\u25B2", "-1" = "\u25BC"),
                     limits = c(-1, 1)) +
  facet_grid(tissue ~ sex)+
  labs(tag = "A",
       title = "Gene expression bias for H2 isochore where *Lep* and *LncLep* genes are located
[Chr6:28,929,476-29,541,870]",
       x = "Gene",
       y = NULL,
       fill = "Bias index",
       shape = "Significance") +
  guides(fill = guide_colorbar(order = 1), shape = guide_legend(override.aes = list(size = 5),
order = 2)) +
  coord_fixed(ratio = 1) +
  theme(panel.background = element_blank(),
        panel.border = element_rect(color = "black", fill = NA, linewidth = 0),
        plot.title = element_markdown(size = 20, hjust = 0.5),
        axis.ticks = element_blank(),
        axis.text = element_text(size = 10, color = "black"),
        axis.text.x = element_text(angle = 270, hjust = 0, vjust = 0.5),
        axis.text.y = element_text(hjust = 0.5),
        legend.key = element_blank(),
        strip.background = element_blank(),
        strip.text = element_text(size = 20),
```

```
        text = element_text(family = "sans", size = 10, color = "black"))

#### Figure panel for the correlation between changes in Leptin plasma levels and GWAT Lep gene
expression -----
fig_panel_lep_corr <- ggplot(leptin_correlations,
                             aes(x = observed_BI,
                                 y = observed_BI_Lep,
                                 color = sex)) +
  geom_point() +
  geom_text_repel(aes(label = condition_a), direction = "y") +
  sm_statCorr(corr_method = "pearson", fit.params = list(linetype = "dotted"), legends = TRUE)
+
  scale_color_manual(values = c("red", "blue")) +
  labs(tag = "A",
       x = "uMCW BI leptin plasma level",
       y = "uMCW BI *Lep* gWAT expression",
       color = NULL) +
  theme(axis.title.y = element_markdown(),
        text = element_text(size = 15))

#### Figure panel for the correlation between changes in GWAT expression for genes Lep and
Lnclep -----
fig_panel_lep_lnclep_corr <- ggplot(leptin_correlations,
                                    aes(x = observed_BI_Lep,
                                        y = observed_BI_Lnclep,
                                        color = sex)) +
  geom_point() +
  geom_text_repel(aes(label = condition_a), direction = "y") +
  sm_statCorr(corr_method = "pearson", fit.params = list(linetype = "dotted"), legends = TRUE)
+
  scale_color_manual(values = c("red", "blue")) +
  labs(tag = "A",
       x = "uMCW BI *Lep* gWAT expression",
       y = "uMCW BI *Lnclep* gWAT expression",
       color = NULL) +
  theme(axis.title = element_markdown(),
        text = element_text(size = 15))

# Saving RData file
save.image(file = "2026_Diaz_Castillo_et_al.RData")

# 9. DRAWING MAIN FIGURES ----
## 9.1. Drawing timeline figure panels ----
### 9.2.a. Figure panel for F0 timeline ----
fig_panel_F0_timeline <- ggplot(fread("Supplementary Data 1.csv")[generation == "F0", ]) +
  geom_point(data = ~.x[.x$start == .x$end, ], aes(x = start,
                                                   y = factor(group,
                                                              levels = c("Semiweekly water
consumption", "Weekly food consumption", "4-hour fasting bodyweight", "Bodyweight", "Data
timepoints")),
                                                   size = 20)) +
  geom_label(data = ~.x[.x$group == "Data timepoints", ], aes(y = group,
                                                              x = start,
                                                              label = event),
             size = 5, label.padding = unit(0, "lines"), label.r = unit(0, "lines"), label.size
= 0, fill = "gray90") +
  geom_linerange(data = ~.x[.x$start != .x$end, ],
                 aes(xmin = start,
                     xmax = end,
                     y = factor(group,
                                levels = c("Mating start", "Female preconception exposure",
"UCSC acclimation",  "Birth at Jackson Laboratories", "Weeks of age")),
                     colour = factor(color)),
                 size = I(9)) +
  geom_text(aes(y = group, x = mid, label = event, size = 20)) +
  scale_x_date(position = "top", date_breaks = "7 days", labels = NULL, expand = c(0, 0)) +
  scale_colour_manual(values = c("a" = "gray90", "b" = "gray80", "c" = "lightblue", "d" =
"red")) +
  labs(tag = "A",
```

```
       title = "F0 experimental timeline",
       x = NULL,
       y = NULL) +
  theme_minimal() +
  theme(plot.title = element_text(hjust = 0.5, size = 20),
        legend.position = "none",
        panel.grid.major.y = element_blank(),
        axis.text.y = element_text(colour = "black", size = 15))

### 9.2.b. Figure panel for F1 timeline ----
fig_panel_F1_timeline <-  ggplot(fread("Supplementary Data 1.csv")[generation == "F1", ]) +
  geom_point(data = ~.x[.x$start == .x$end, ], aes(x = start,
                                                    y = factor(group,
                                                               levels = c("Cardiac blood and
tissue harvesting", "12-hour fasting glucose", "12-hour fasting bodyweight", "Bodyweight",
"Data timepoints")),
                                                    size = 20)) +
  geom_label(data = ~.x[.x$group == "Data timepoints", ], aes(y = group,
                                                               x = start,
                                                               label = event),
             size = 5, label.padding = unit(0, "lines"), label.r = unit(0, "lines"), label.size
= 0, fill = "gray90") +
  geom_linerange(data = ~.x[.x$start != .x$end, ],
                 aes(xmin = start,
                     xmax = end,
                     y = factor(group, levels = c("Postmortem plasma and tissue harvesting",
"Diet", "Weaning", "Birth", "Weeks of age")),
                     colour = factor(color)),
                 size = I(9)) +
  geom_text(aes(y = group, x = mid, label = event, size = 20)) +
  scale_x_date(position = "top", date_breaks = "7 days", labels = NULL, expand = c(0, 0)) +
  scale_colour_manual(values = c("a" = "gray90", "b" = "gray80", "c" = "lightblue", "d" =
"orange", "e" = "red")) +
  labs(tag = "A",
       title = "F1 experimental timeline",
       x = NULL,
       y = NULL) +
  theme_minimal() +
  theme(plot.title = element_text(hjust = 0.5, size = 15),
        legend.position = "none",
        panel.grid.major.y = element_blank(),
        axis.text.y = element_text(colour = "black", size = 12))

## 9.2. Drawing multipanel figures ----
### 9.2.a. Drawing and saving Figure 1 to the “File confirmations/Figures” subdirectory ----
figure1 <- free(fig_panel_F0_timeline) /
  free(fig_panel_F0_BW_raw) /
  (fig_panel_F0_BW + fig_panel_F0_fBW + fig_panel_F0_water + fig_panel_F0_food_g +
fig_panel_F0_food_kcal + plot_layout(ncol = 5, axes = "collect_y", guides = "collect")) +
  plot_annotation(tag_levels = "a") + plot_layout(design = c(area(1, 1, 1, 5), area(2, 1, 2,
5), area(3, 1, 3, 5)), heights = c(3, 2, 2)) &
  theme(text = element_text(size = 15), plot.tag = element_text(size = 20, hjust = 0, vjust =
0, face = "bold"))

ggsave("Figure_1.png", plot = figure1, path = "./File confirmations/Figures/", width = 25,
height = 15, dpi = 300)

### 9.2.b. Drawing and saving Figure 2 to the “File confirmations/Figures” subdirectory ----
figure2 <- (fig_panel_F1_timeline + fig_panel_F1_litter) /
  (fig_panel_F1_BW_f + fig_panel_F1_fBW_f + fig_panel_F1_fglucose_f + fig_panel_F1_tissues_f +
plot_layout(ncol = 4, axes = "collect_y", guides = "collect")) /
  (fig_panel_F1_BW_m + fig_panel_F1_fBW_m + fig_panel_F1_fglucose_m + fig_panel_F1_tissues_m +
plot_layout(ncol = 4, axes = "collect_y", guides = "collect")) /
  (fig_panel_F1_metabolites_f + fig_panel_PCA_F1_plasma_f_biplot) /
  (fig_panel_F1_metabolites_m + fig_panel_PCA_F1_plasma_m_biplot) +
  plot_layout(design = c(area(1, 2.5, 1, 5.5), area(2, 1, 2, 6), area(3, 1, 3, 6), area(4, 1,
4, 6), area(5, 1, 5, 6))) +
  plot_annotation(tag_levels = list(c("a", "b", "c", "e", "g", "i", "d", "f", "h", "j", "k",
"m", "l", "n"))) &
```

```
  theme(text = element_text(size = 12), plot.tag = element_text(size = 20, face = "bold"))

ggsave("Figure_2.png", plot = figure2, path = "./File confirmations/Figures/", width = 25,
height = 20, dpi = 300)

### 9.2.c. Drawing and saving Figure 3 to the “File confirmations/Figures” subdirectory3 ----
figure3 <- (as.ggplot(fig_panel_clustering) +
              labs(title = "Clustering of the top 10,000 more expressed genes") +
              theme(plot.title = element_text(size = 20, hjust = 0.5, vjust = 0.5))) +
  (fig_panel_fgsea +
     labs(title = "Gene Set Enrichment Analyses (GSEA) for Gene Ontology Terms") +
     theme(plot.title = element_text(size = 20, hjust = 0.5, vjust = 0.5))) +
  plot_annotation(tag_levels = "a") &
  theme(plot.tag = element_text(size = 20, face = "bold"))

ggsave("Figure_3.png", plot = figure3, path = "./File confirmations/Figures/", width = 30,
height = 20, dpi = 300)

### 9.2.d. Drawing and saving Figure 4 to the “File confirmations/Figures” subdirectory ----
figure4 <- (fig_panel_chr12_isochore + fig_panel_bMCW_whole + plot_layout(widths = c(4, 1))) /
  fig_panel_bMCW_isochore_chr /
  (fig_panel_isochore_chr + fig_panel_isochore_GO + plot_layout(axes = "collect_y", guides =
"collect")) +
  plot_annotation(tag_levels = "a") &
  theme(plot.tag = element_text(size = 20, face = "bold"))

ggsave("Figure_4.png", plot = figure4, path = "./File confirmations/Figures/", width = 25,
height = 25, dpi = 300)

### 9.2.e. Drawing and saving Figure 5 to the “File confirmations/Figures” subdirectory ----
figure5 <- (fig_panel_GWAT_gene_isochore_BIs / fig_panel_liver_gene_isochore_BIs) +
  plot_annotation(tag_levels = "a") &
  theme(plot.tag = element_text(size = 20, face = "bold"))

ggsave("Figure_5.png", plot = figure5, path = "./File confirmations/Figures/", width = 25,
height = 15, dpi = 300)

### 9.2.f. Drawing and saving Figure 6 to the “File confirmations/Figures” subdirectory ----
figure6 <- fig_panel_Lep_isochore /
  ((fig_panel_lep_corr +
      labs(title = "Leptin plasma levels and *Lep* gWAT expression correlation") +
      theme(plot.title = element_markdown(size = 20, face = "plain", hjust = 0.5, vjust =
0.5))) +
     (fig_panel_lep_lnclep_corr +
        labs(title = "*Lep* and *Lnclep* gWAT expression correlation") +
        theme(plot.title = element_markdown(size = 20, face = "plain", hjust = 0.5, vjust =
0.5)))) +
  plot_annotation(tag_levels = "a") &
  theme(plot.tag = element_text(size = 20, face = "bold"))

ggsave("Figure_6.png", plot = figure6, path = "./File confirmations/Figures/", width = 25,
height = 15, dpi = 300)

# Saving RData file
save.image(file = "2026_Diaz_Castillo_et_al.RData")

# 10. CLEAN UP ----
# This section will remove “temp” subdirectory
unlink("temp", recursive = TRUE)

# Saving RData file
save.image(file = "2026_Diaz_Castillo_et_al.RData")
```
