# Supplementary figures and images for: Preconception exposures of female mice to a panel of metabolic disruptors induce sexually dimorphic metabolic perturbations in their offspring
Source: Front Endocrinol (Lausanne). 2026 Apr 2;17:1787973. doi: 10.3389/fendo.2026.1787973 (PMC13082944; doi:10.3389/fendo.2026.1787973)

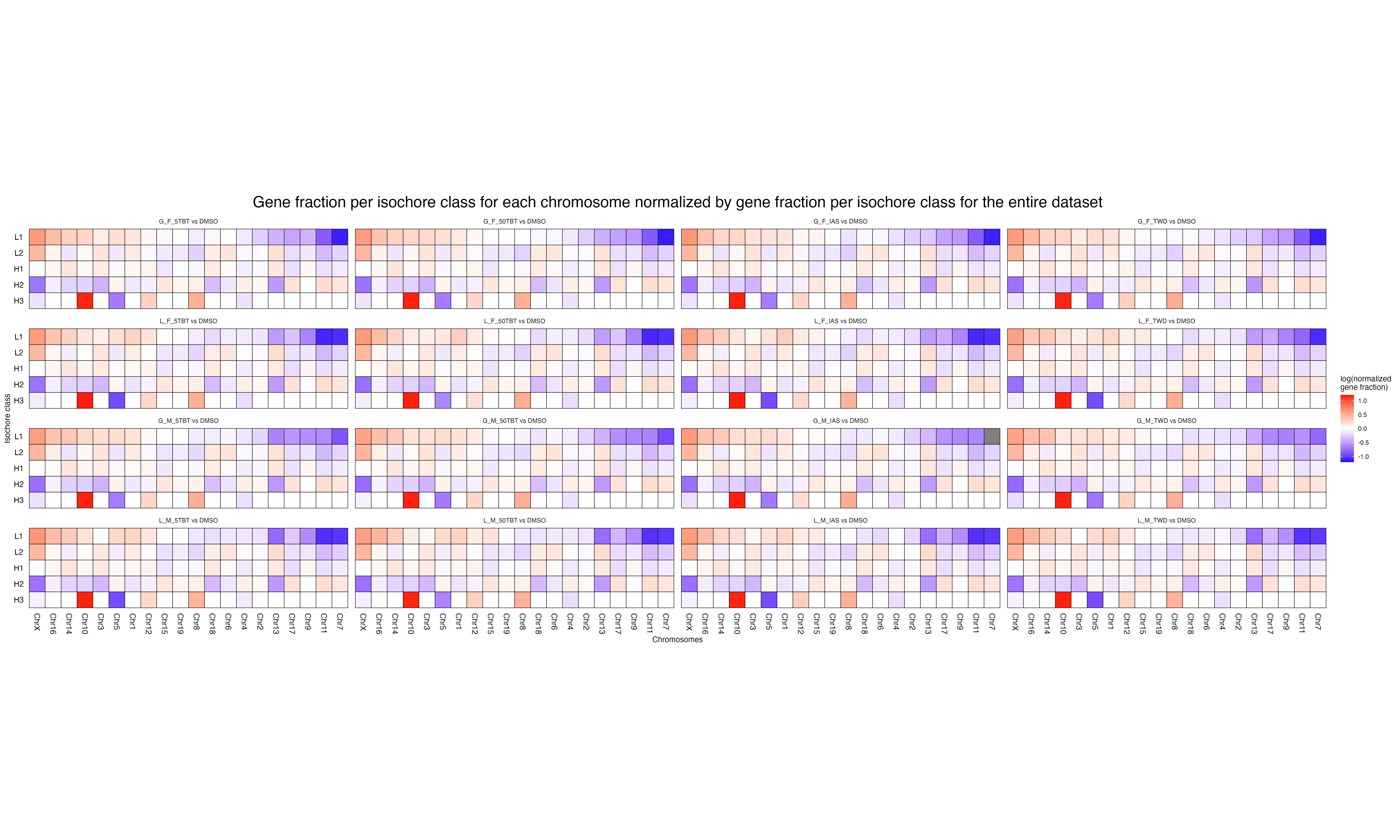

Supplement: Supplementary file 1 [file Supplementaryfile1.zip › Data Sheet 1_updated/Supplementary Figure 1.png]

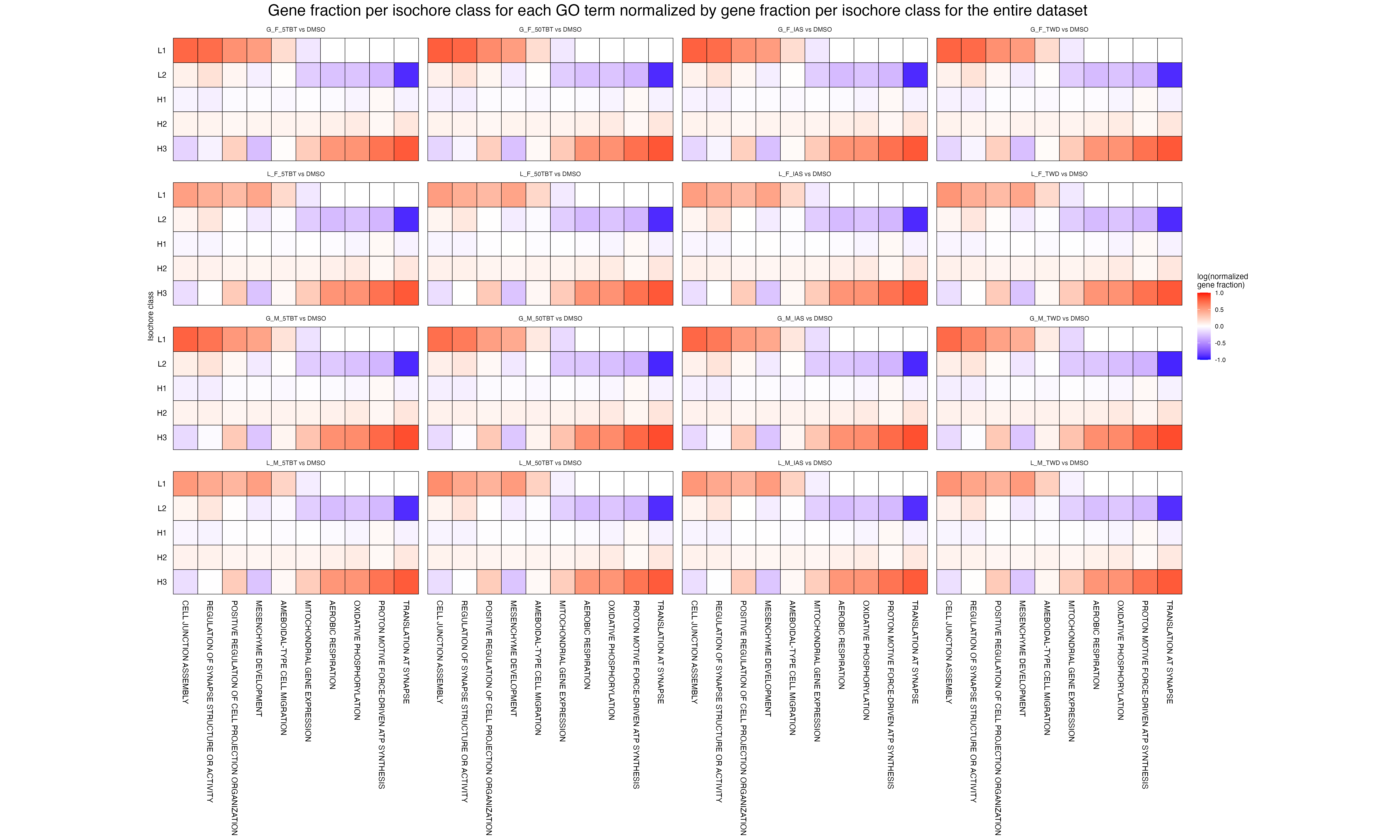

Supplement: Supplementary file 1 [file Supplementaryfile1.zip › Data Sheet 1_updated/Supplementary Figure 2.png]
